# Supplementary material for: Long-term whole blood DNA preservation by cost-efficient cryosilicification
Source: Nat Commun. 2022 Oct 21;13:6265. doi: 10.1038/s41467-022-33759-y (PMC9587218; doi:10.1038/s41467-022-33759-y)
Supplement: Supplementary file 1 — Supporting Information [file 41467_2022_33759_MOESM1_ESM.pdf]

Supplementary Materials

Long-Term Whole Blood DNA Preservation by Cost-Efficient Cryosilicification

Liang Zhou,[a,‡] Qi Lei,[a,‡] Jimin Guo,[b,‡] Yuanyuan Gao,[a] Jianjun Shi,[c] Hong Yu,[a] Wenxiang Yin,[a] Jiangfan Cao,[a] Botao Xiao,[a] Jacopo Andreo,[d] Romy Ettlinger,[e] C. Jeffrey Brinker,[b] Stefan Wuttke,[d,f,*] and Wei Zhu[a,*]

[a] MOE International Joint Research Laboratory on Synthetic Biology and Medicines, School of Biology and Biological Engineering, South China University of Technology, Guangzhou 510006, P. R. China.

[b] Center for Micro-Engineered Materials and the Department of Chemical and Biological Engineering, The University of New Mexico, Albuquerque, New Mexico 87131, USA.

[c] Science and Technology on Advanced Functional Composites Technology, Aerospace Research Institute of Materials & Processing Technology, Beijing 100076, P. R. China

[d] BCMaterials, Basque Center for Materials, UPV/EHU Science Park, 48940 Leioa, Spain.

[e] School of Chemistry, University of St. Andrews, St. Andrews, United Kingdom.

[f] Ikerbasque, Basque Foundation for Science, Bilbao, Spain.

[‡] These authors contributed equally to this work.

[*] Correspondence to: weizhu86@scut.edu.cn; stefan.wuttke@bcmaterials.net

Table of Contents

Supplementary figures 3
Supplementary tables 47

Supplementary figures

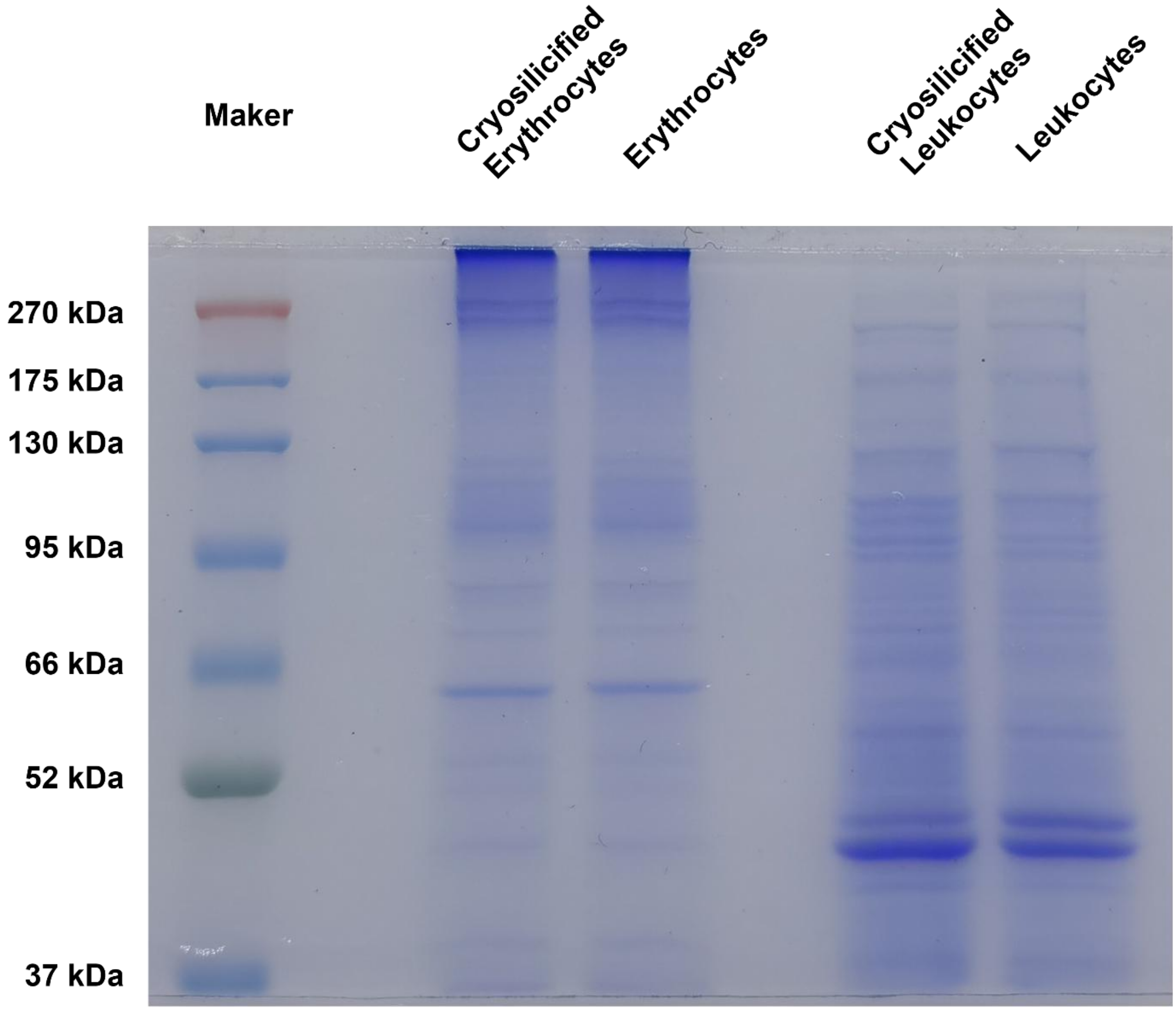

Supplementary Fig 1. SDS-PAGE analysis of the cell membrane protein of erythrocytes and the whole cell protein of leukocytes after cryosilicification. A representative image of three biological replicates is shown.

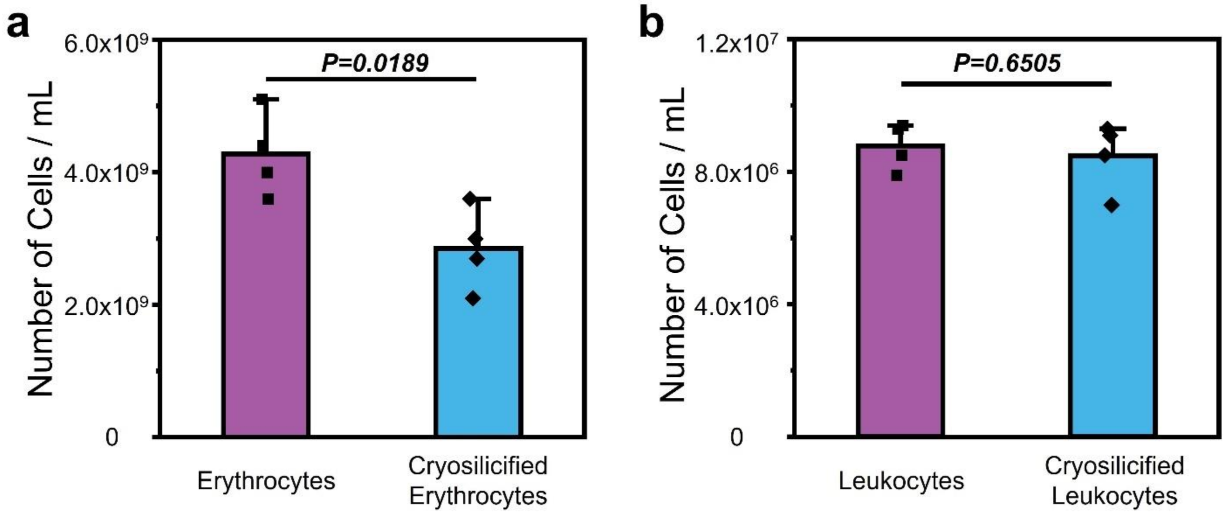

Supplementary Fig 2. Yield of erythrocytes (a) and leukocytes (b) after cryosilicification. (mean± standard deviation, n = 4 biologically independent samples, two-tailed t-test).

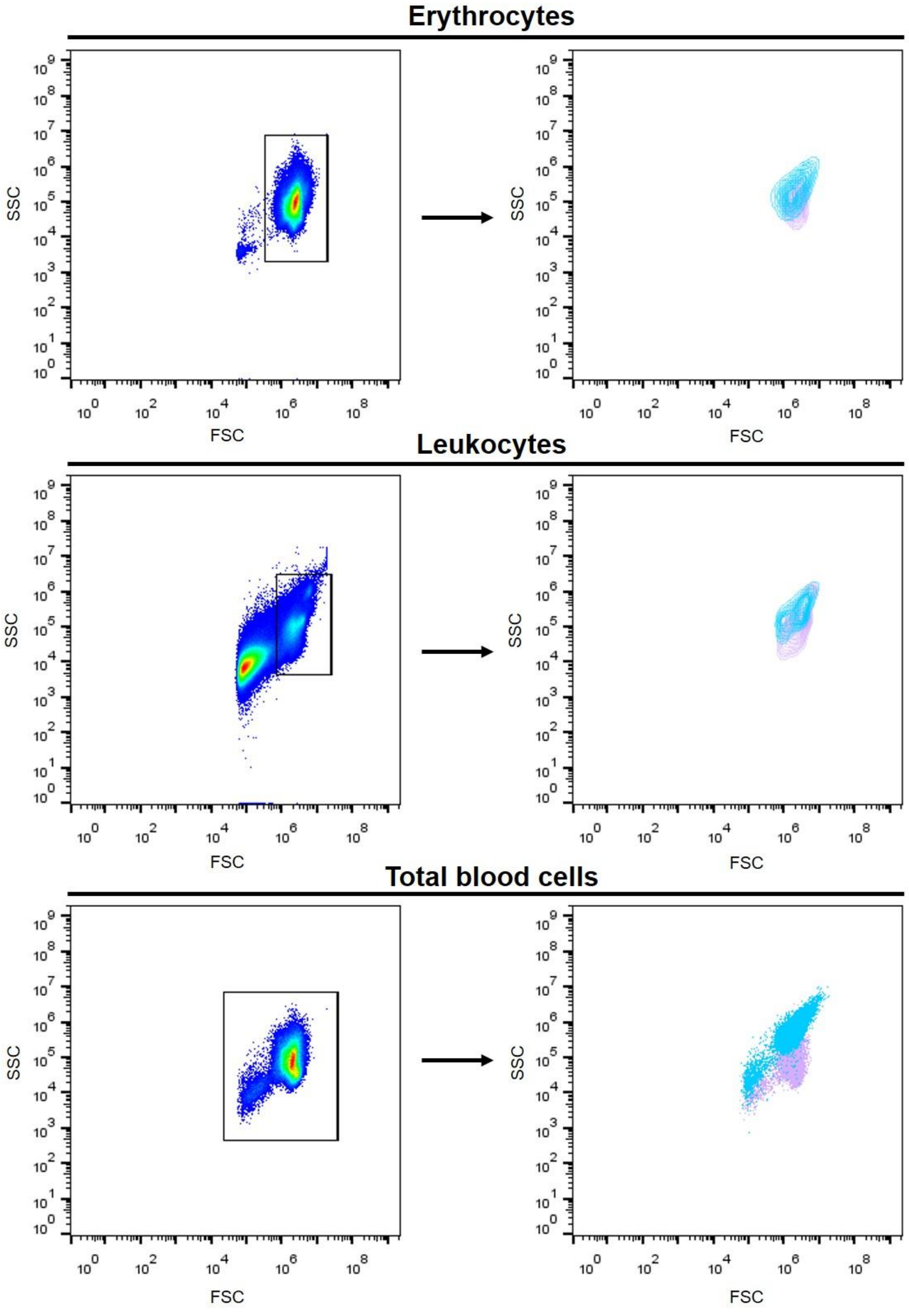

Supplementary Fig 3. Flow cytometry analysis of untreated (purple) and cryosilicified (blue) erythrocytes (a), leukocytes (b), and total blood cells (c) at the same voltages. FSC, forward scatter, SSC, side scatter.

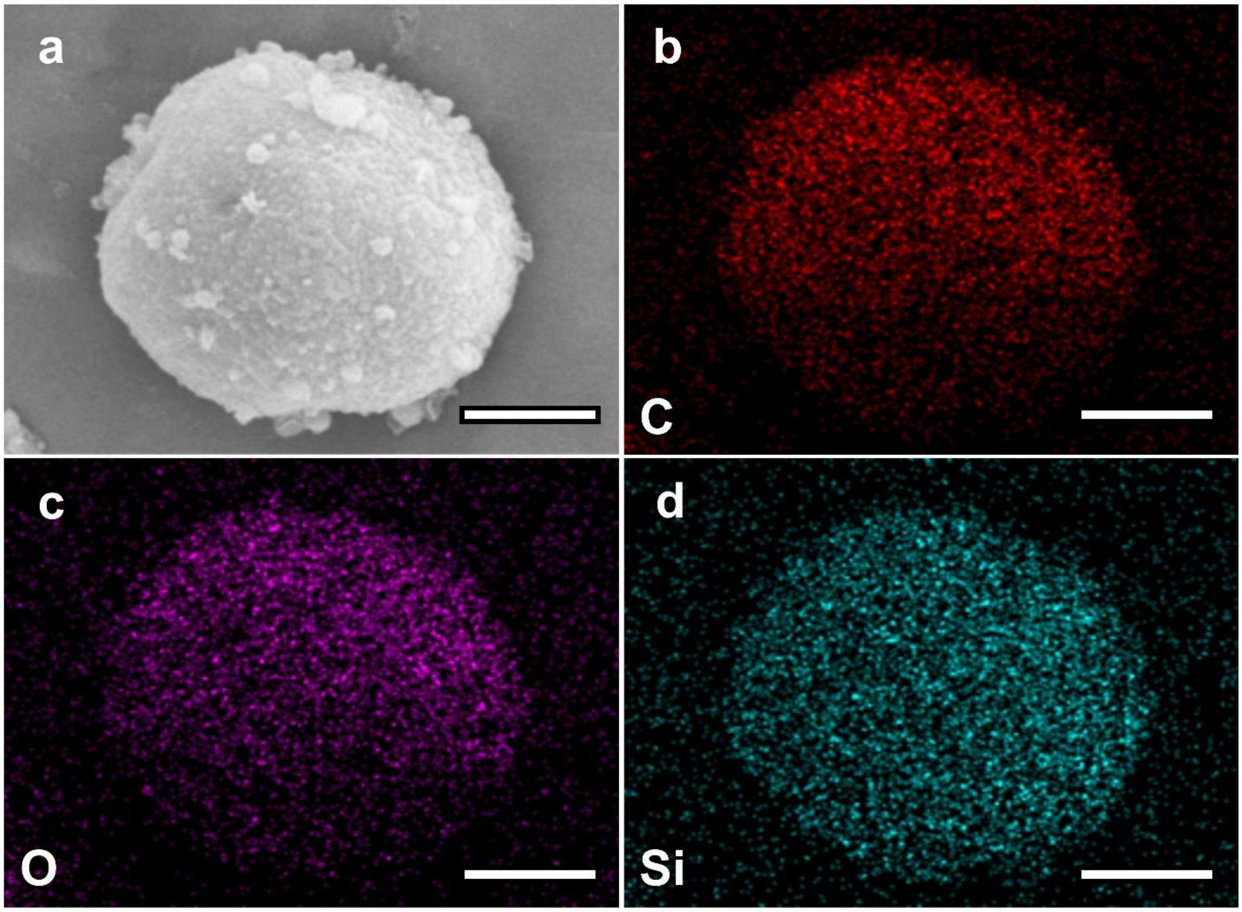

Supplementary Fig 4. SEM image (a) of cryosilicified leukocyte and corresponding EDS maps of C (b), O (c) and Si (d), Scale bar = 2 μm. A representative image of two biological replicates is shown.

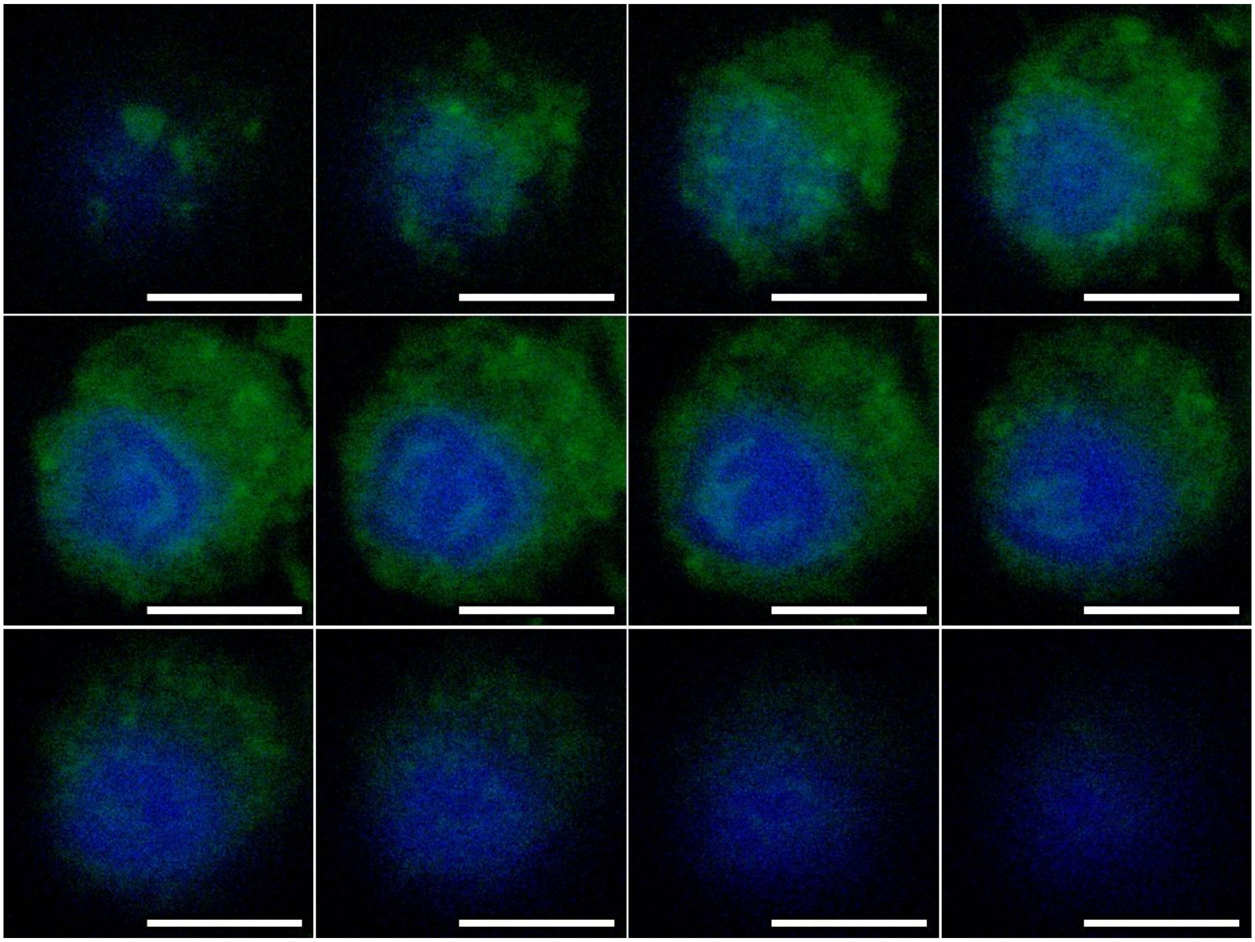

Supplementary Fig 5. Z-stack confocal images of 4 ^{o}C silicified leukocyte with green fluorescence of the silica signal. Scale bar = 5 μm. A representative image of three biological replicates is shown.

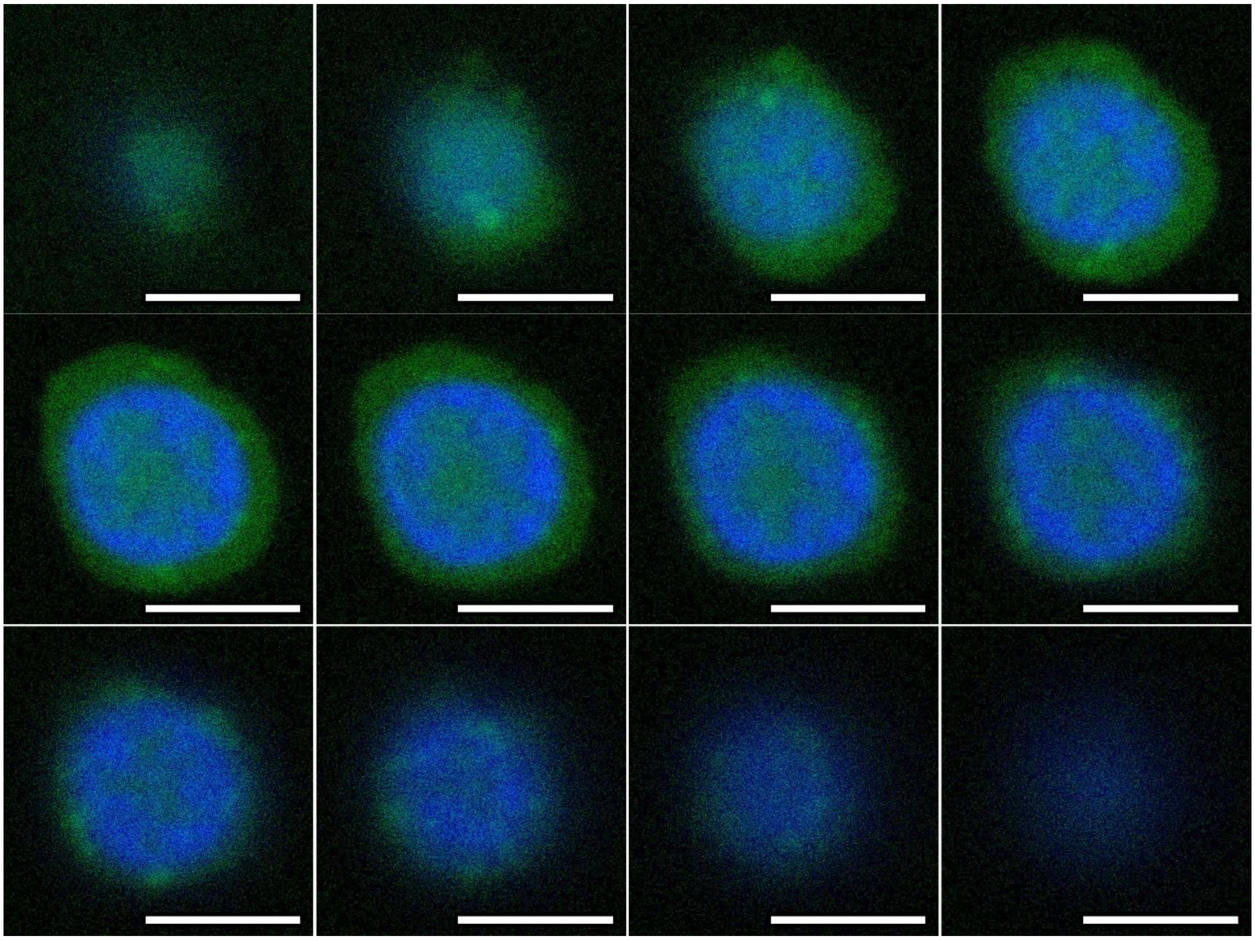

Supplementary Fig 6. Z-stack confocal images of cryosilicified leukocyte with green fluorescence of the silica signal as cytoskeleton and inside the nucleus; Scale bar = 5 μm. A representative image of three biological replicates is shown.

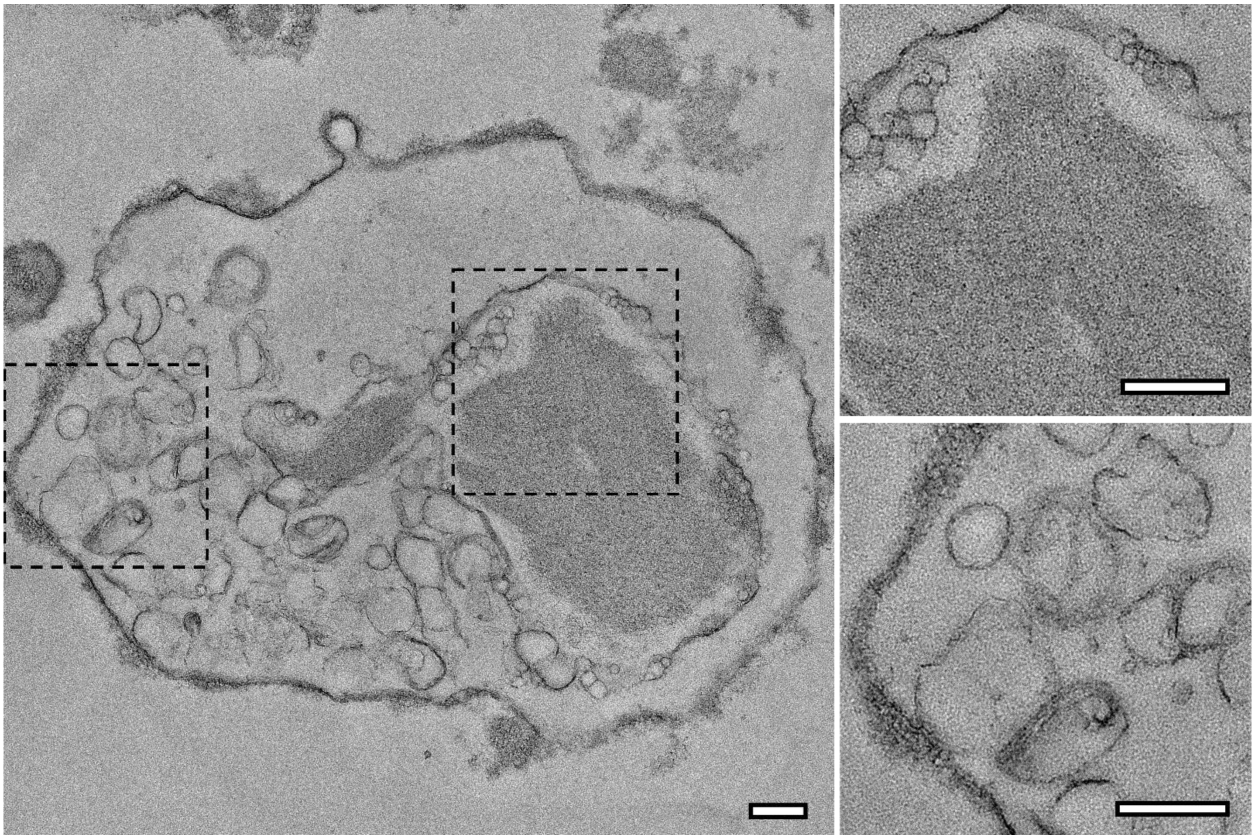

Supplementary Fig 7. Cross-sectional TEM images of microtome-sliced cryosilicified leukocytes. Scale bar = 200 nm. A representative image of three biological replicates is shown.

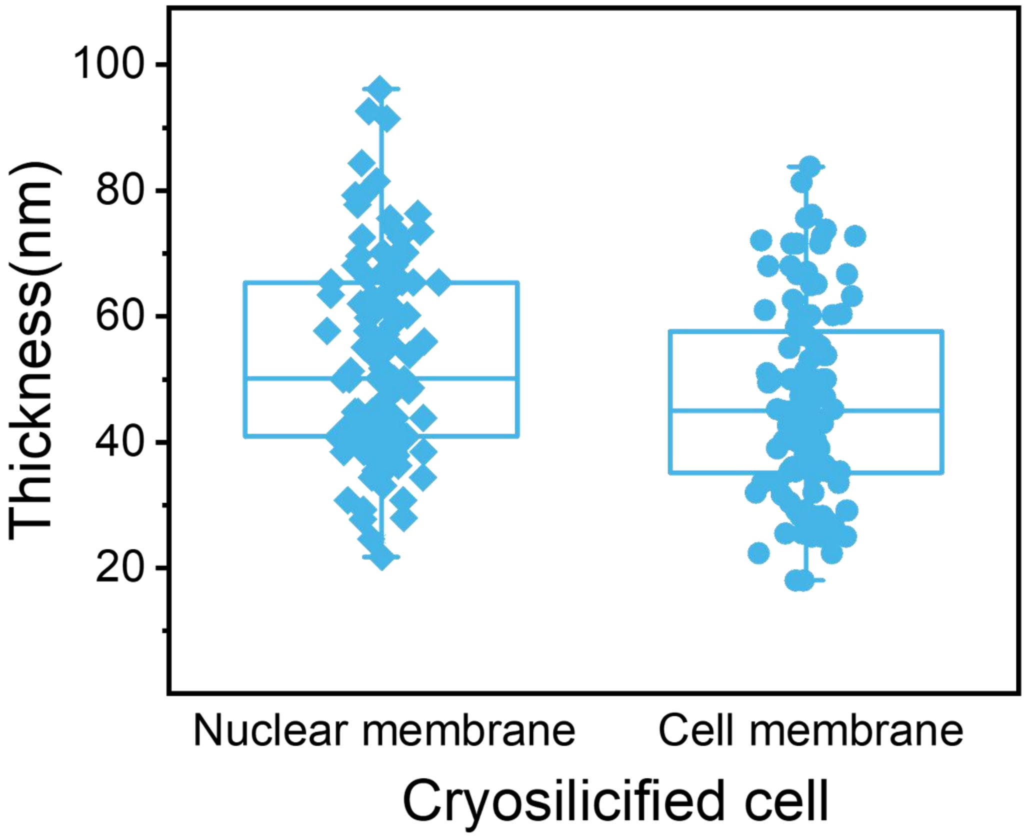

Supplementary Fig 8. Silica layer thickness of leukocyte cell membrane and nuclear membrane after cryosilicification at random region in Supplementary Fig 7 measuring by ImageJ. Box and whiskers represent mean ± 25-75 percentile. Centre values 50.177 and 45.071. (Nuclear membrane, n = 102 regions; Cell membrane, n = 108 regions)

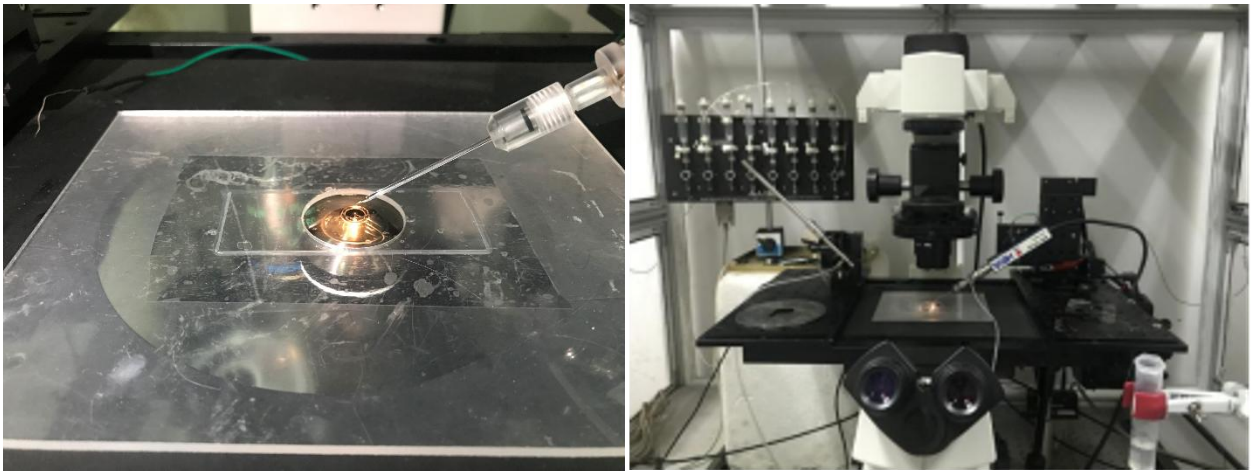

Supplementary Fig 9. Optical images of homemade micropipette aspiration instrument for Young’s modulus measurement.

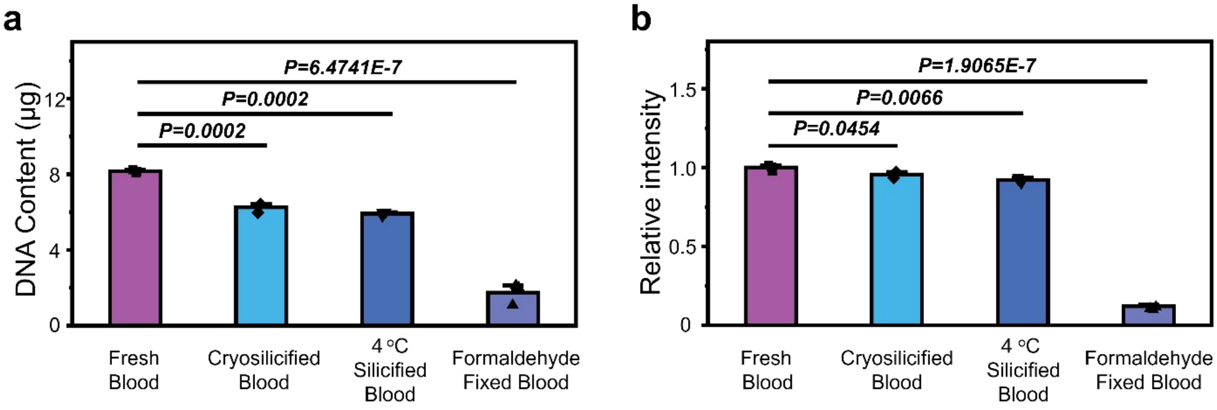

Supplementary Fig 10. (a) The amount of extracted DNA from 1mL of fresh blood, cryosilicified blood, 4 ºC silicified blood and formaldehyde fixed blood samples (mean ± standard deviation, n = 3 biologically independent samples, two-tailed t-test), and (b) their relative intensity on gel electrophoresis (mean ± standard deviation, n = 3 independent analyses, two-tailed t-test).

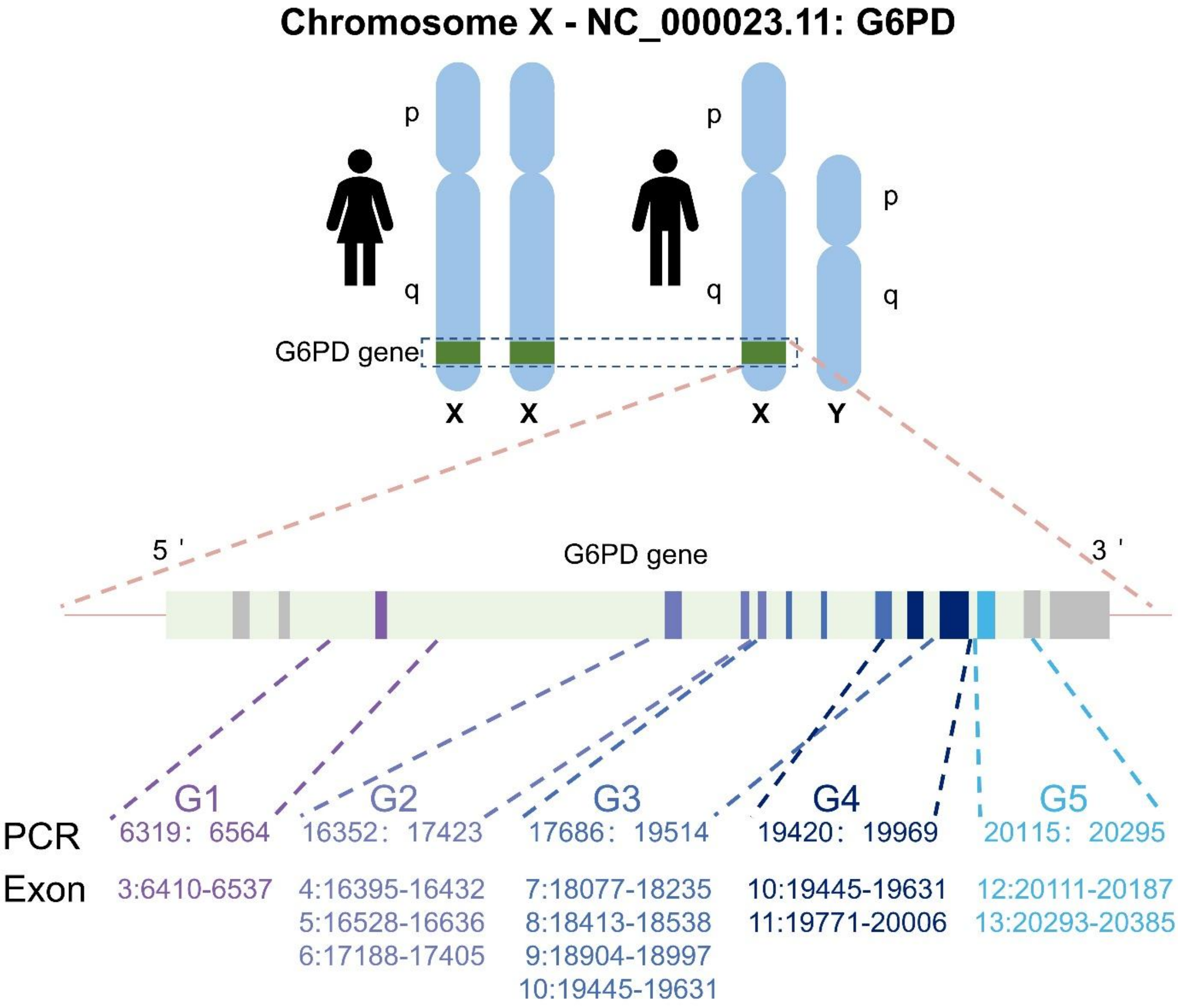

Supplementary Fig 11. Information of chromosomally-encoded housekeeping gene G6PD.

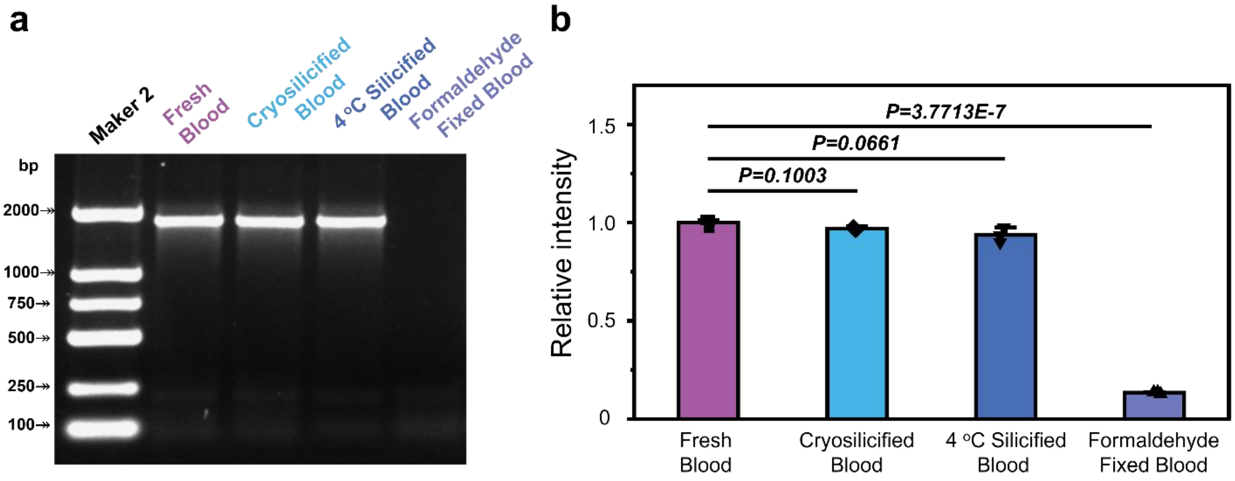

Supplementary Fig 12. (a) Gel electrophoresis of the amplified target genomic fragments (G3, 1730 bp) from fresh blood, cryosilicified blood, 4 °C silicified blood and formaldehyde fixed blood samples. A representative image of three biological replicates is shown. (b) Their relative intensity (mean ± standard deviation, n = 3 independent analyses, two-tailed t-test).

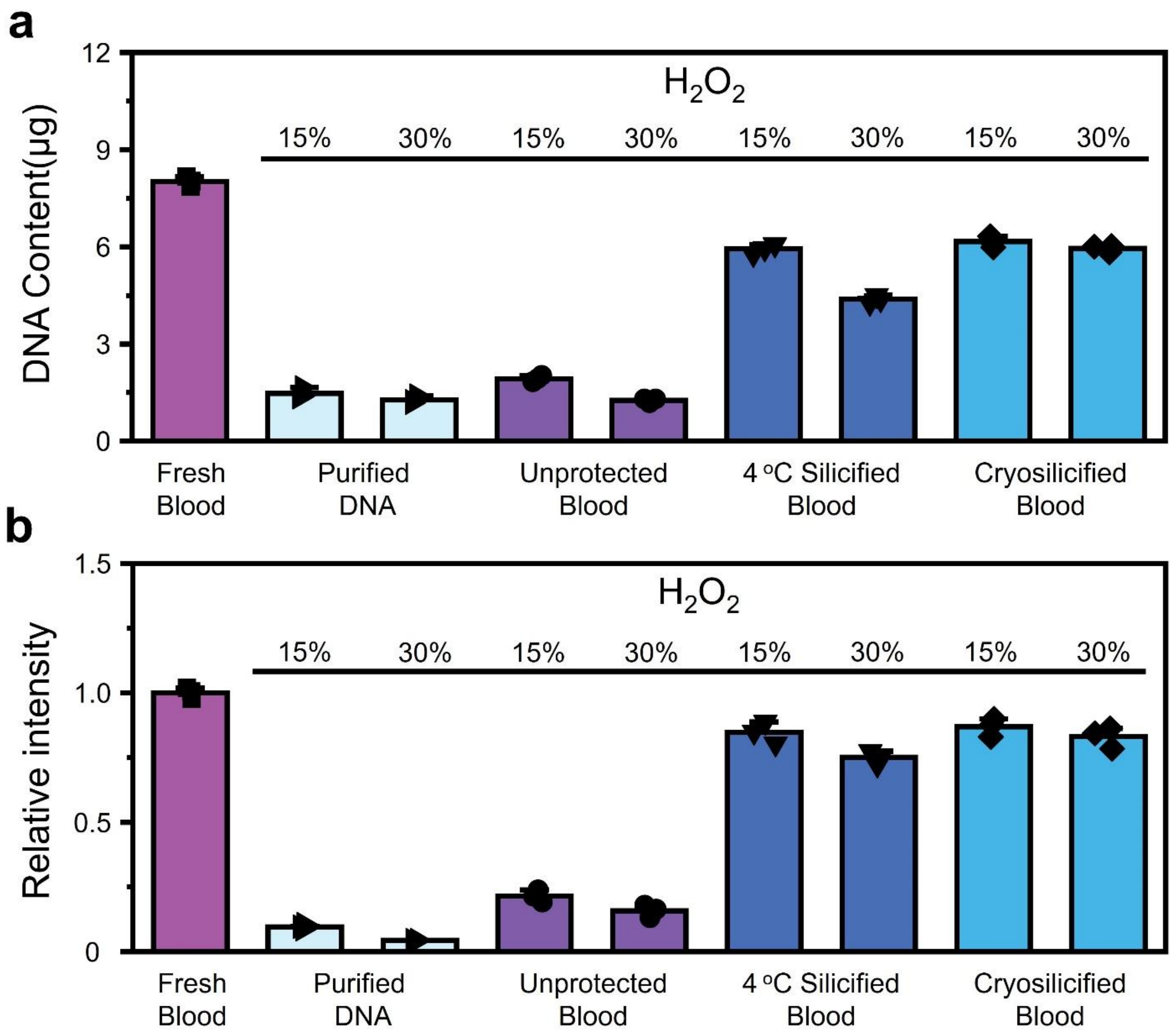

Supplementary Fig 13. (a) The amount of extracted DNA from fresh blood, purified DNA, unprotected blood, 4 °C silicified blood and cryosilicified blood samples after H_2O_2 treatment. (mean ± standard deviation, n = 3 biologically independent samples), and (b) their relative intensity on gel electrophoresis (mean ± standard deviation, n = 3 independent analyses).

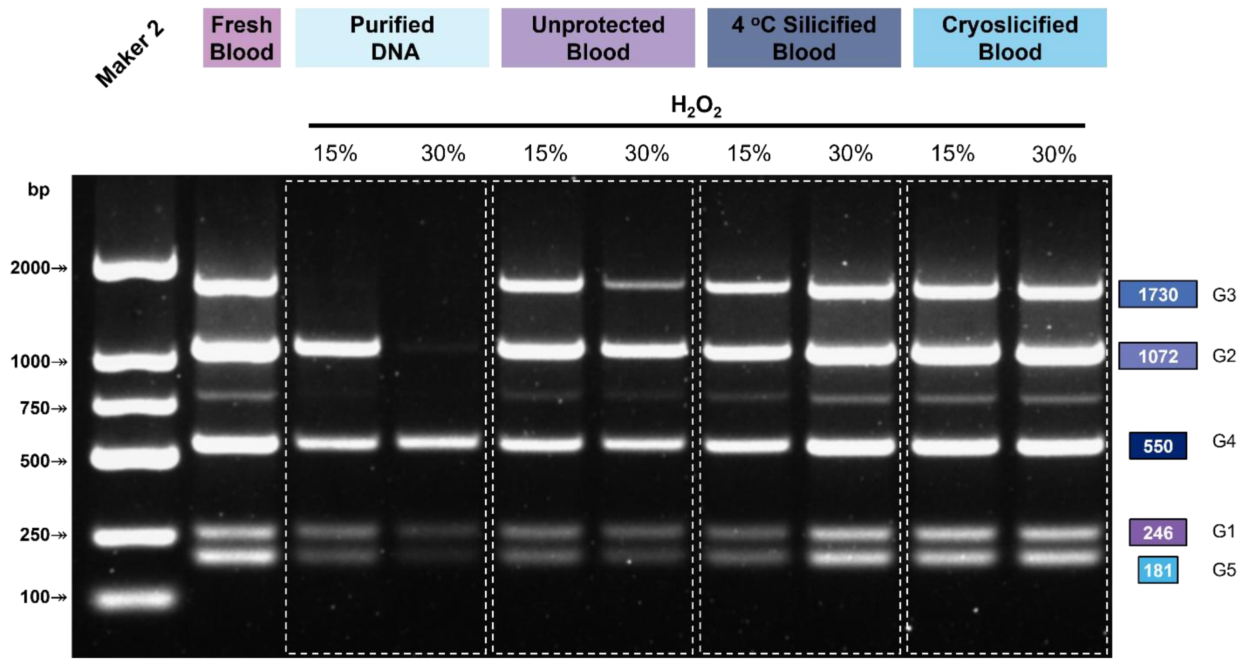

Supplementary Fig 14. Gel electrophoresis of the amplified target genomic fragments (G1-G5) from fresh blood, purified DNA, unprotected blood, 4 °C silicified blood and cryosilicified blood samples after H_2O_2 treatment. A representative image of three biological replicates is shown.

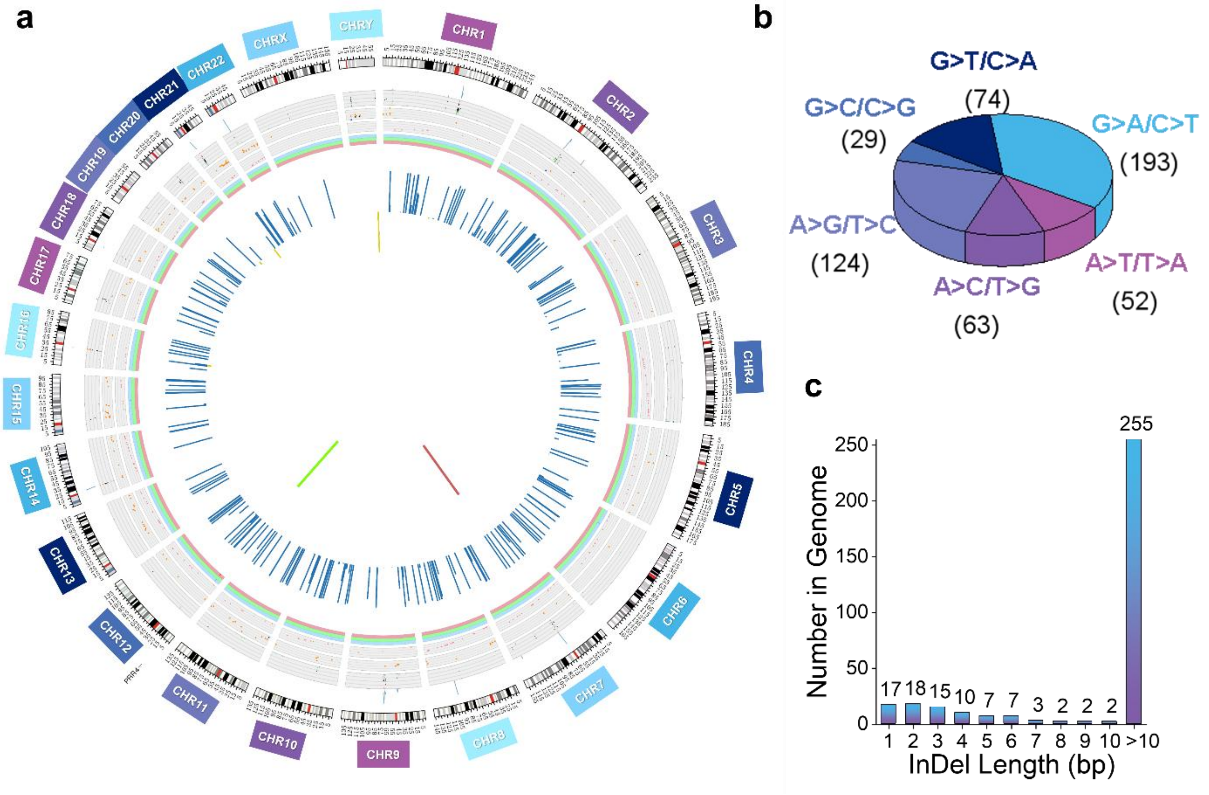

Supplementary Fig 15. (a) Circos plot summary of mutations between fresh blood samples and cryosilicified blood samples with H_2O_2 treatment (30% H_2O_2, 4 h) in all human chromosomes (1-22+XY); (b) SNP mutations between fresh blood samples and cryosilicified blood samples with H_2O_2 treatment. (c) InDel mutations between fresh blood samples and cryosilicified blood samples with H_2O_2 treatment.

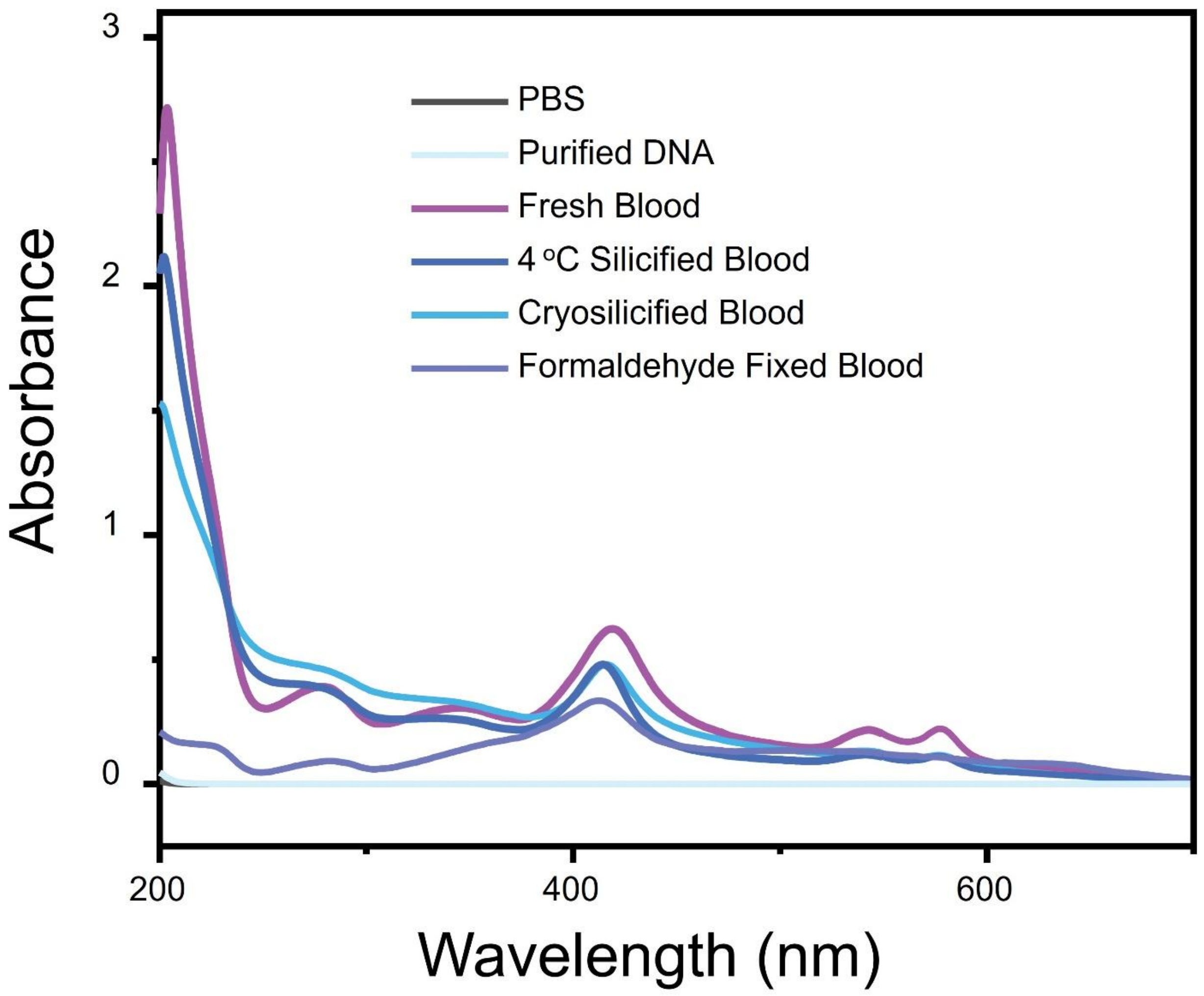

Supplementary Fig 16. UV–Vis absorption spectrum of fresh blood, purified DNA, unprotected blood, 4 °C silicified blood, cryosilicified blood samples, and formaldehyde fixed blood samples.

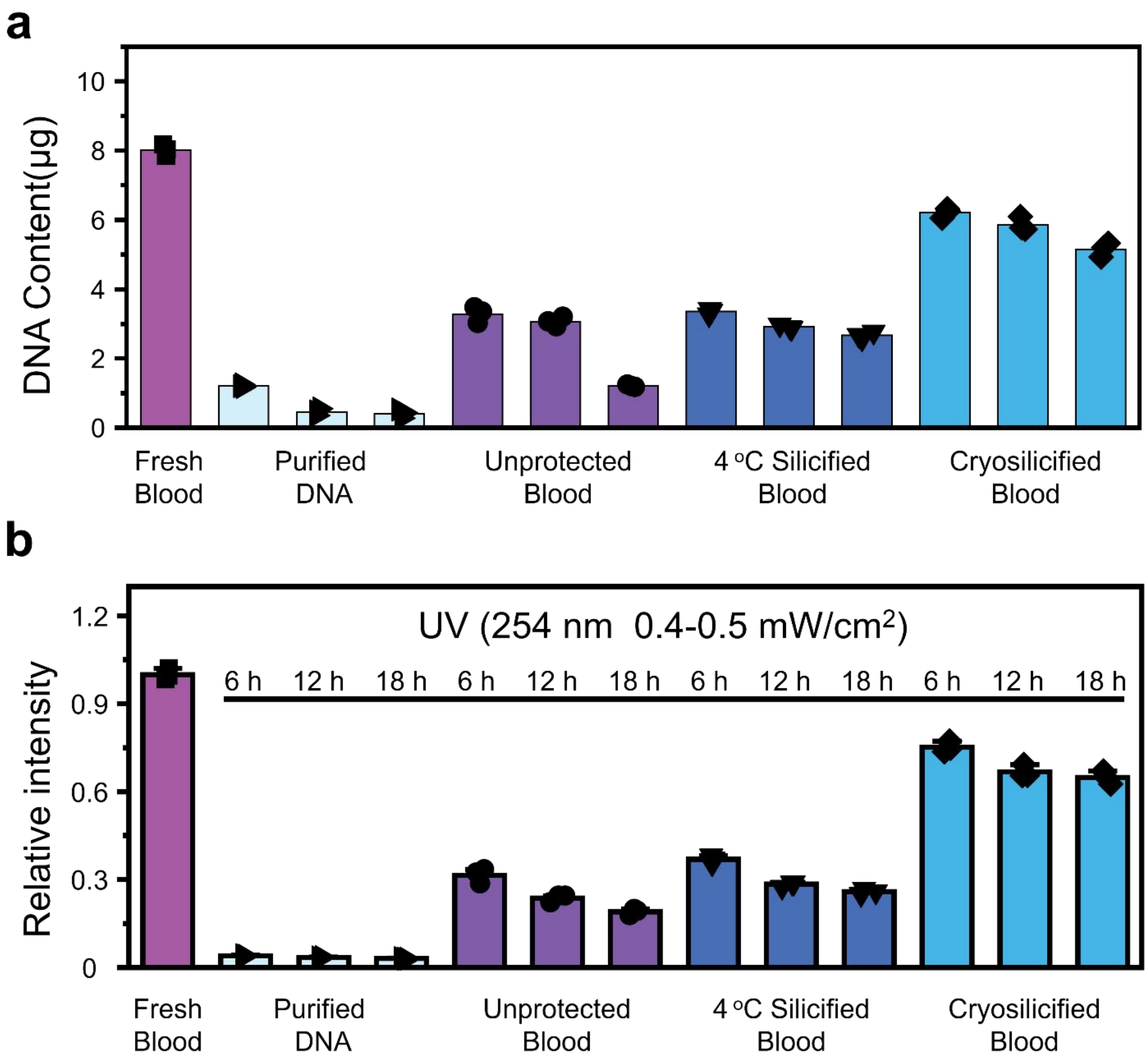

Supplementary Fig 17. (a) The amount of extracted DNA from fresh blood, purified DNA, unprotected blood, 4 °C silicified blood and cryosilicified blood samples after UV treatment (mean ± standard deviation, n = 3 biologically independent samples), and (b) their relative intensity on gel electrophoresis (mean ± standard deviation, n = 3 independent analyses).

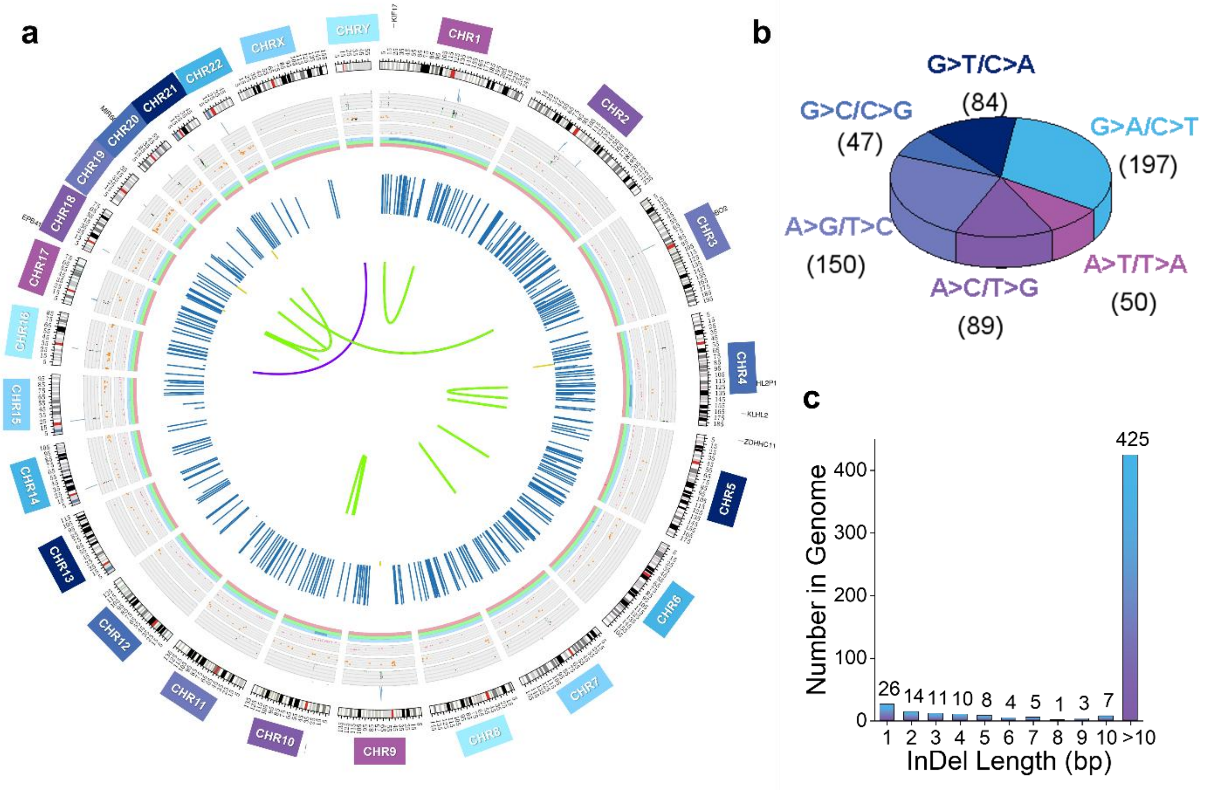

Supplementary Fig 18. (a) Circos plot summary of mutations between fresh blood samples and cryosilicified blood samples with UV irradiation (254 nm, 0.4-0.5 mW cm–2, 16 h) in all human chromosomes (1-22+XY); (b) SNP mutations between fresh blood samples and cryosilicified blood samples with UV irradiation. (c) InDel mutations between fresh blood samples and cryosilicified blood samples with UV irradiation.

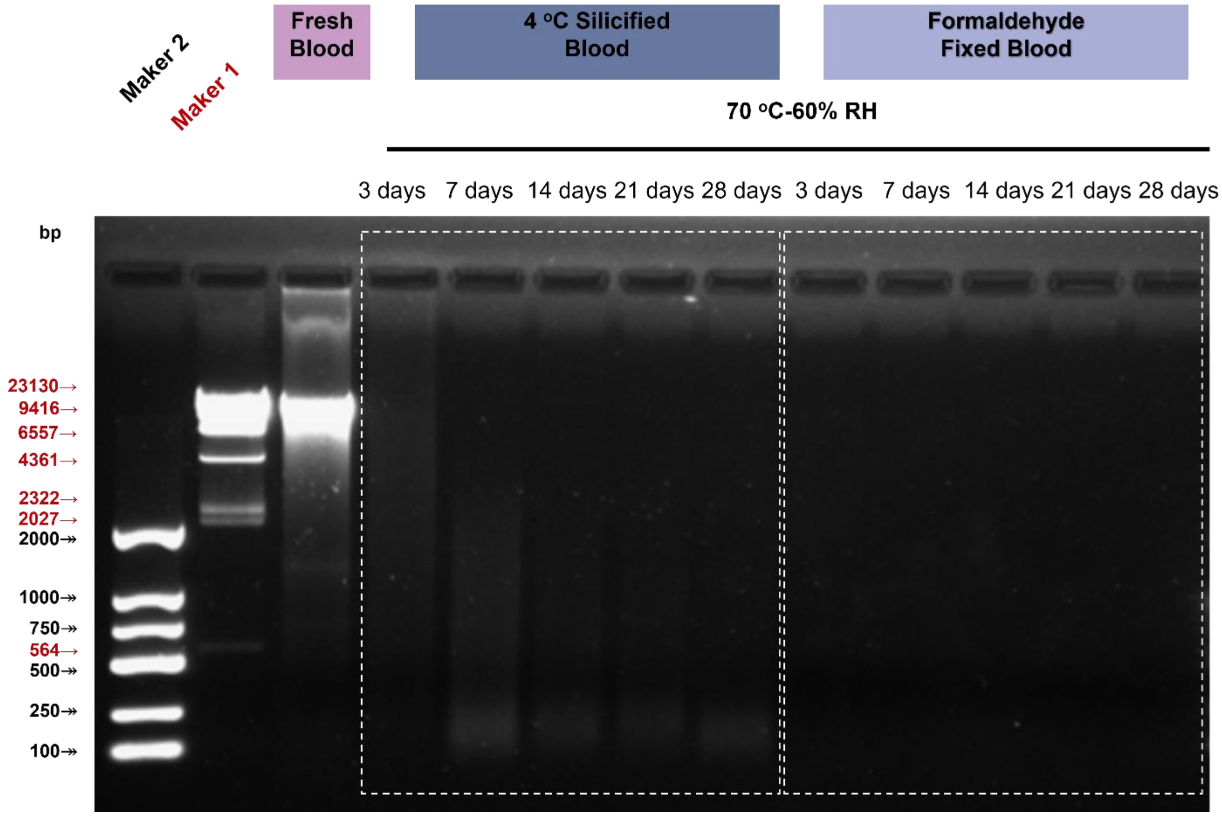

Supplementary Fig 19. Gel electrophoresis of DNA extracted from fresh blood, 4 °C silicified blood, and formaldehyde fixed blood samples aging at 70 °C and 60% RH. A representative image of three biological replicates is shown.

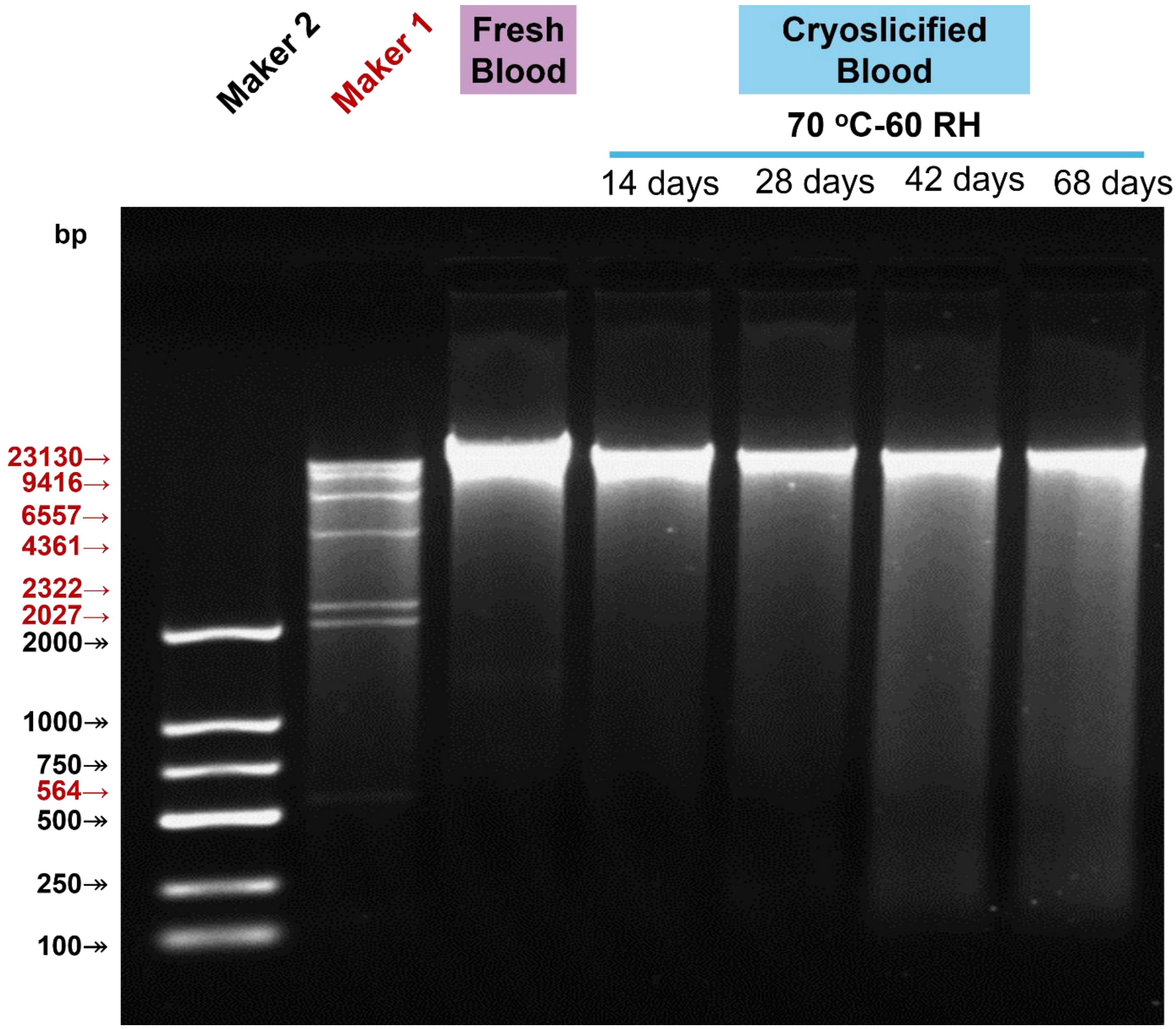

Supplementary Fig 20. Gel-electrophoresis of the DNA from cryosilicified blood samples during the storage at room temperature. A representative image of three biological replicates is shown.

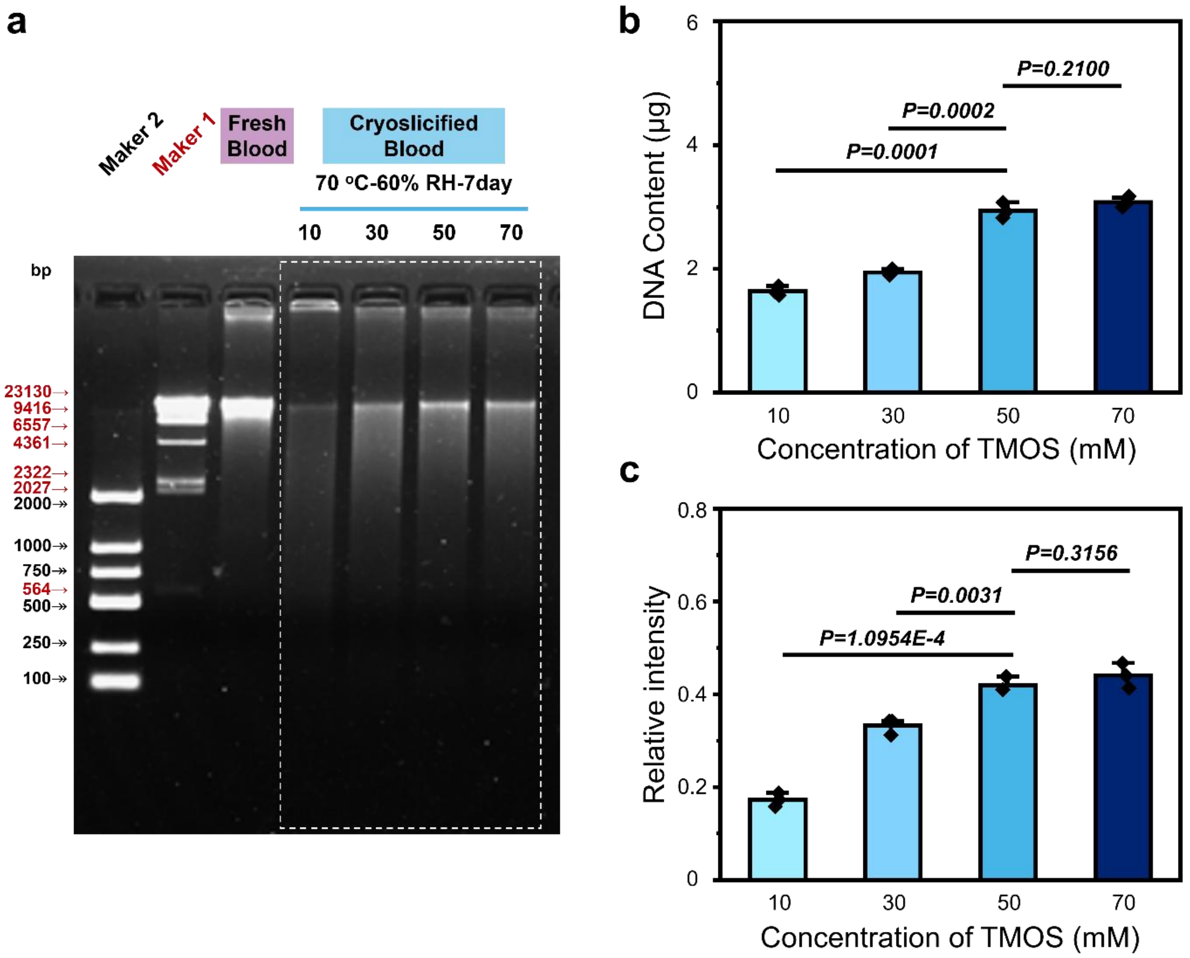

Supplementary Fig 21. (a) Gel electrophoresis of the DNA extracted from cryosilicified blood that was silicified with 10 mM, 30 mM, 50 mM and 70 mM silicic acid and then aging at 70 °C and 60% RH for 7 days. A representative image of three biological replicates is shown. (b) The amount of their extracted DNA (mean ± standard deviation, n = 3 biologically independent samples, two-tailed t-test), and (c) their relative intensity on gel electrophoresis (mean ± standard deviation, n = 3 independent analyses, two-tailed t-test). 50 mM silicic acid was the condition used as standard silicification condition for cryosilicified blood.

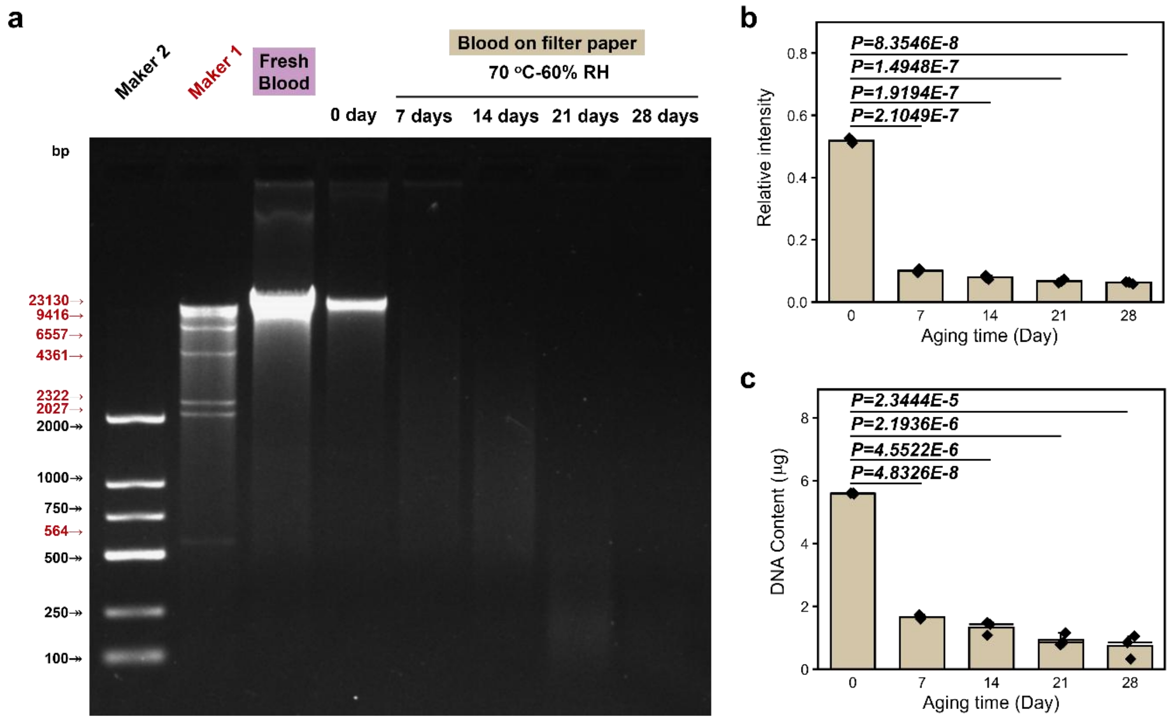

Supplementary Fig 22. (a) Gel-electrophoresis of the DNA extracted from fresh blood sample, and blood sample on filter paper aging at 70 ºC and 60% RH condition. A representative image of three biological replicates is shown. (b) Relative intensity of fresh blood sample (mean ± standard deviation, n = 3 independent analyses, two-tailed t-test), and blood sample on filter paper aging at 70 ºC and 60% RH condition. (c) Extracted DNA amount of the DNA extracted from fresh blood sample (mean ± standard deviation, n = 3 biologically independent samples, two-tailed t-test), and blood sample on filter paper aging at 70 ºC and 60% RH condition.

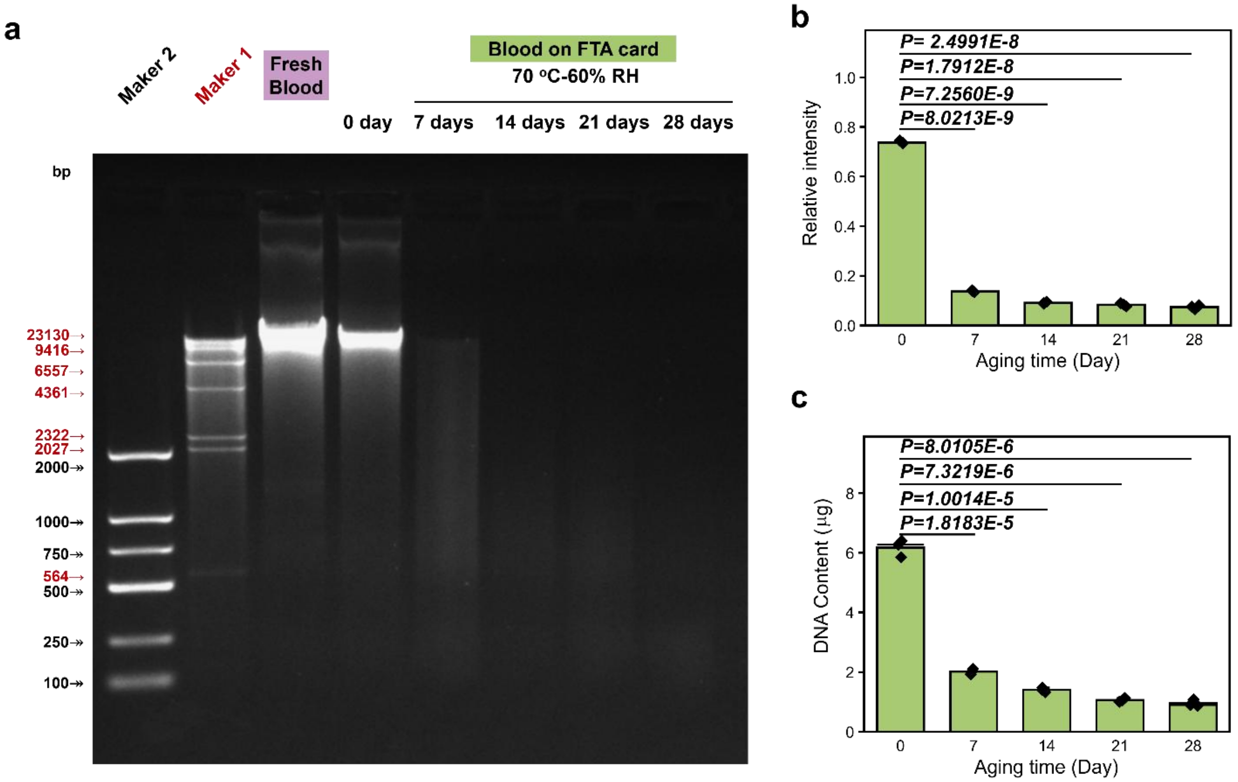

Supplementary Fig 23. (a) Gel-electrophoresis of the DNA extracted from fresh blood sample, and blood sample on FTA card aging at 70 °C and 60% RH condition. A representative image of three biological replicates is shown. (b) Relative intensity of fresh blood sample (mean ± standard deviation, n = 3 independent analyses, two-tailed t-test), and blood sample on FTA card aging at 70 °C and 60% RH condition. (c) Extracted DNA amount of the DNA extracted from fresh blood sample (mean ± standard deviation, n = 3 biologically independent samples, two-tailed t-test), and blood sample on FTA card aging at 70 °C and 60% RH condition.

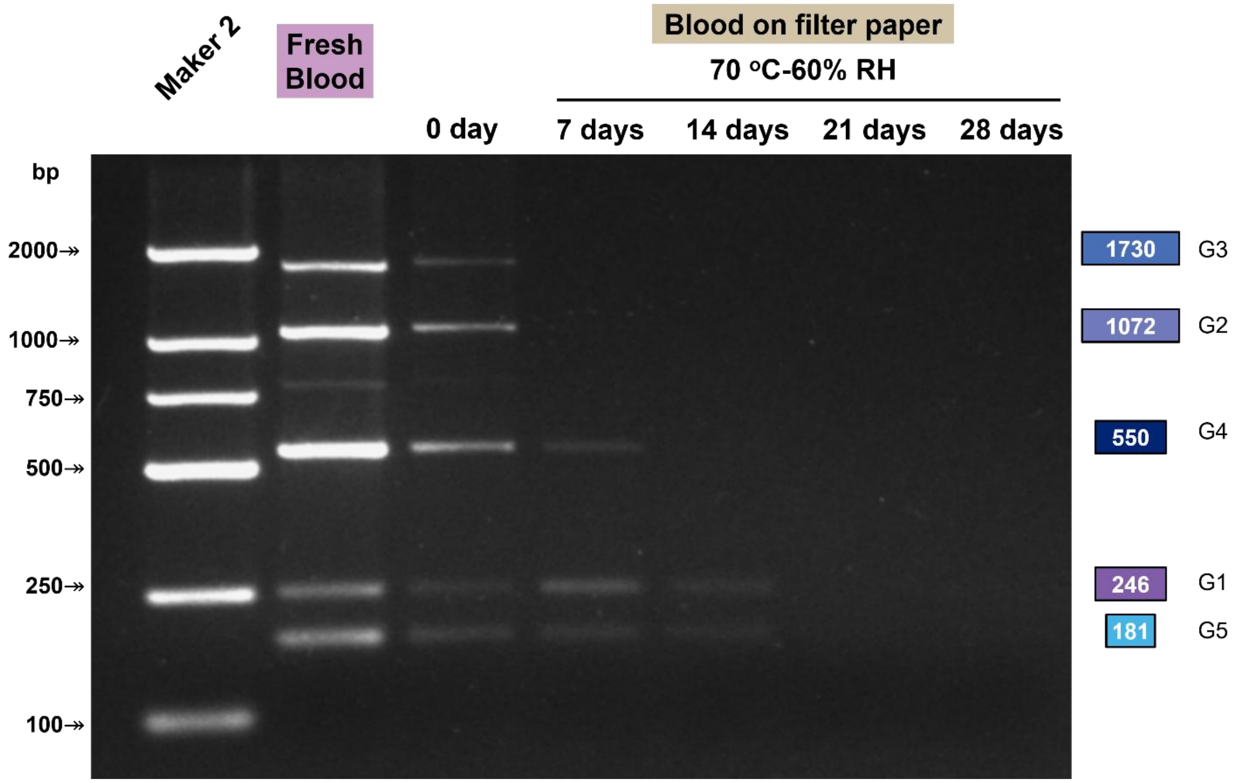

Supplementary Fig 24. Gel-electrophoresis of the amplified target genomic fragments (G1-5) from fresh blood sample and blood sample on filter paper aging at 70 ºC and 60% RH condition. A representative image of three biological replicates is shown.

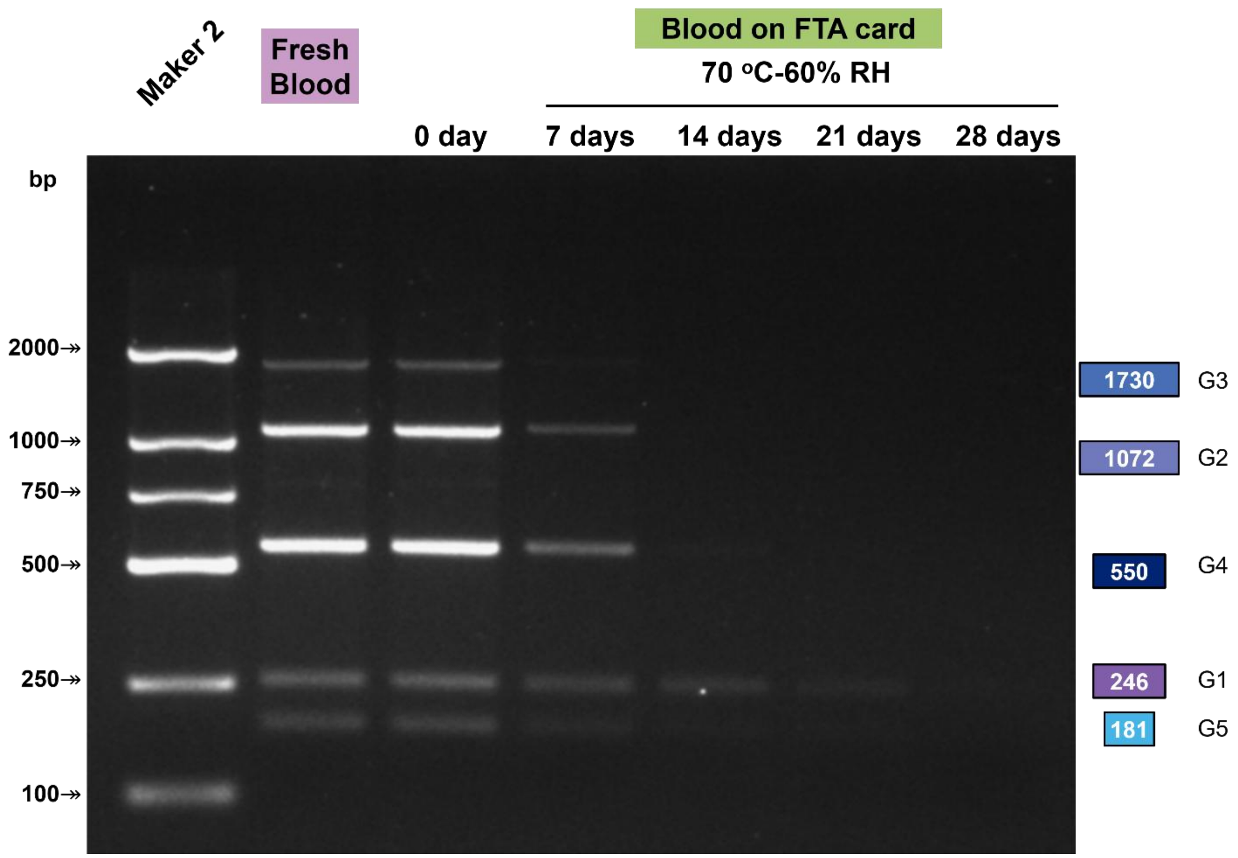

Supplementary Fig 25. Gel-electrophoresis of the amplified target genomic fragments (G1-5) from fresh blood sample and blood sample on FTA card aging at 70 °C and 60% RH condition. A representative image of three biological replicates is shown.

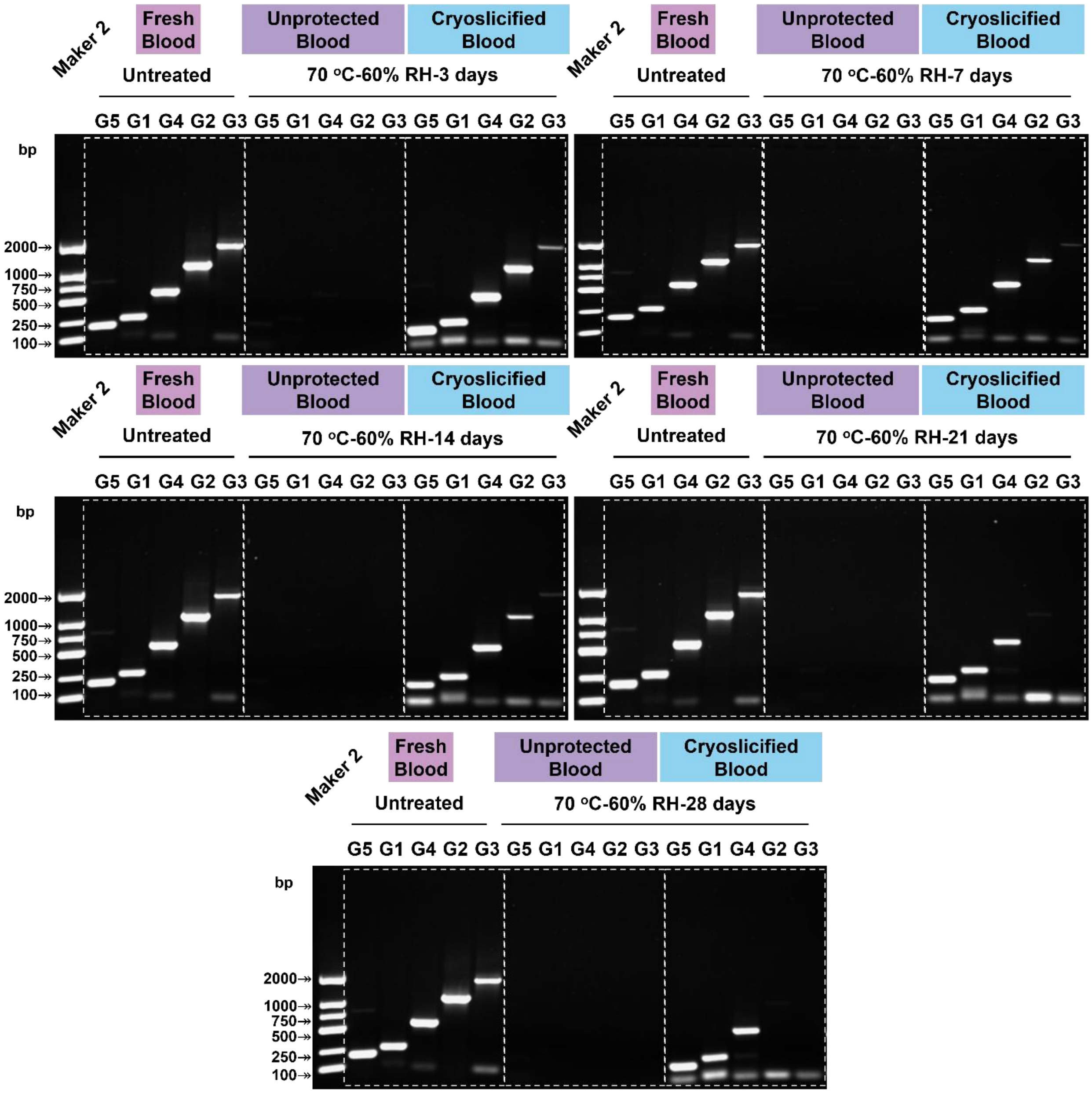

Supplementary Fig 26. Gel electrophoresis of the amplified target genomic fragments (G1-5) from fresh blood, unprotected blood, and cryosilicified blood samples after aging at 70 ºC and 60% RH. A representative image of two biological replicates is shown.

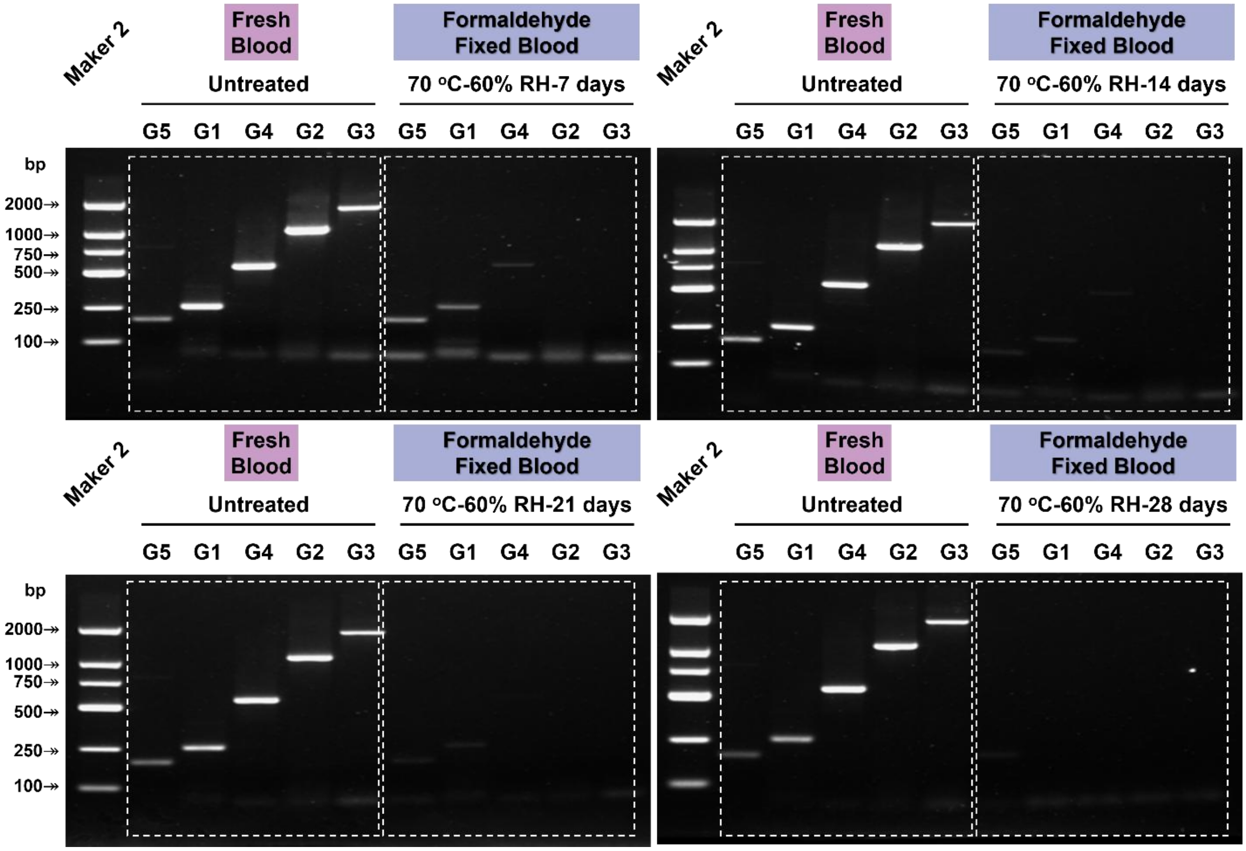

Supplementary Fig 27. Gel electrophoresis of the amplified target genomic fragments (G1-5) from fresh blood sample and formaldehyde fixed blood samples after aging at 70 °C and 60% RH. A representative image of two biological replicates is shown.

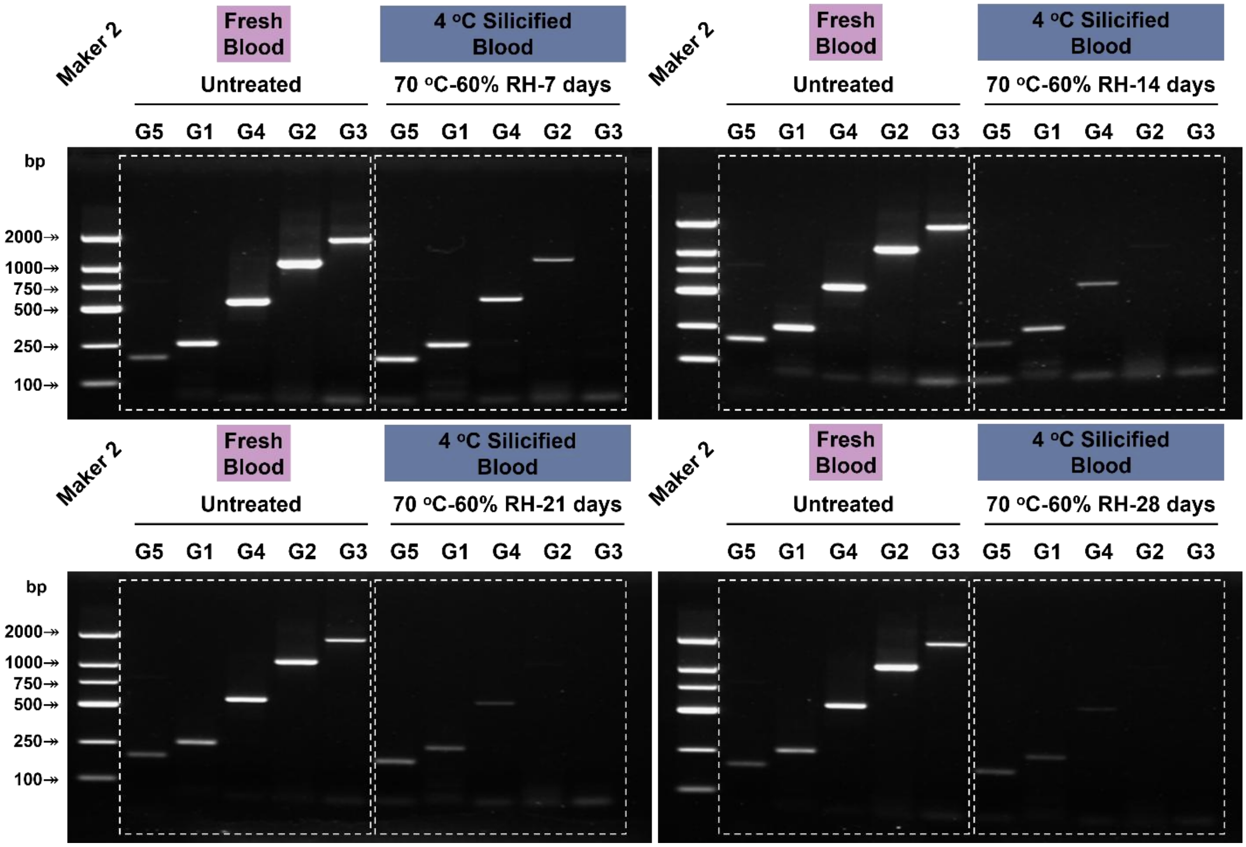

Supplementary Fig 28. Gel electrophoresis of the amplified target genomic fragments (G1-5) from fresh blood sample and 4 °C silicified blood samples after aging at 70 °C and 60% RH. A representative image of two biological replicates is shown.

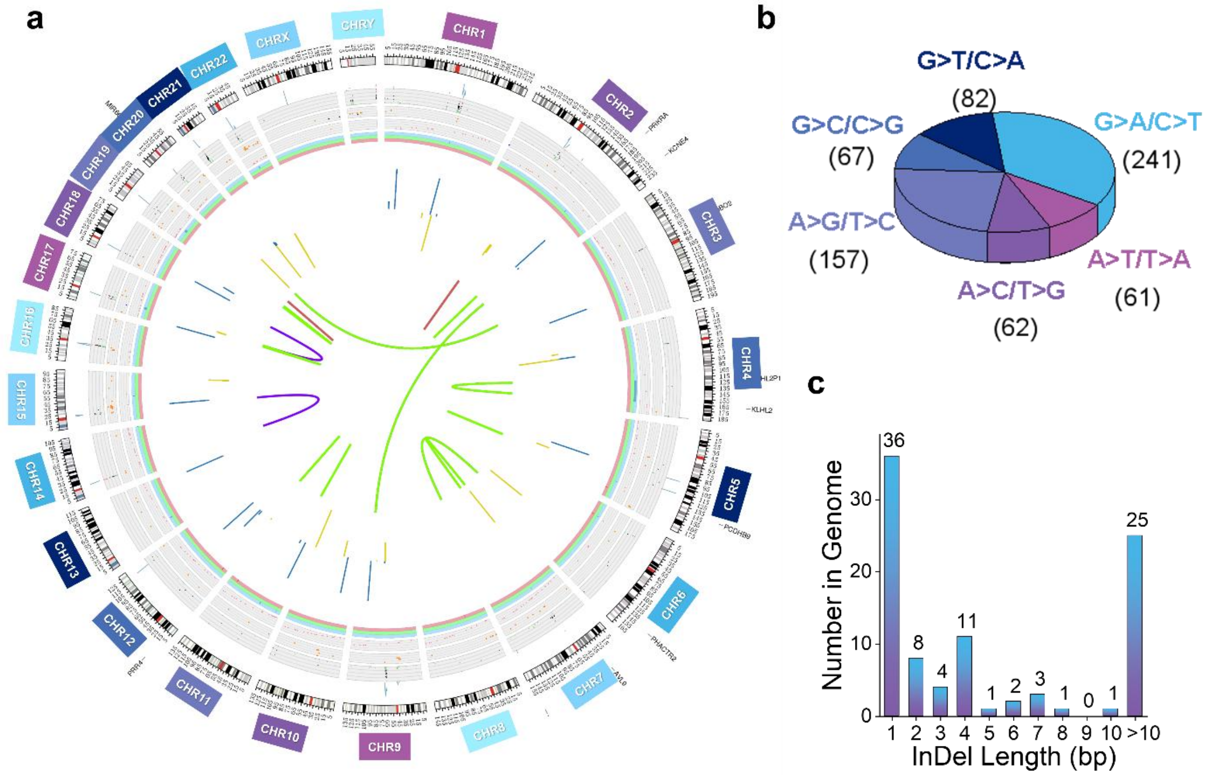

Supplementary Fig 29. (a) Circos plot summary of mutations between fresh blood samples and cryosilicified blood samples with 7-days storage (at 70 ^{o}C and 60% RH condition) in all human chromosomes (1-22+XY); (b) SNP mutations between fresh blood samples and cryosilicified blood samples with 7-days storage. (c) InDel mutations between fresh blood samples and cryosilicified blood samples with 7-days storage.

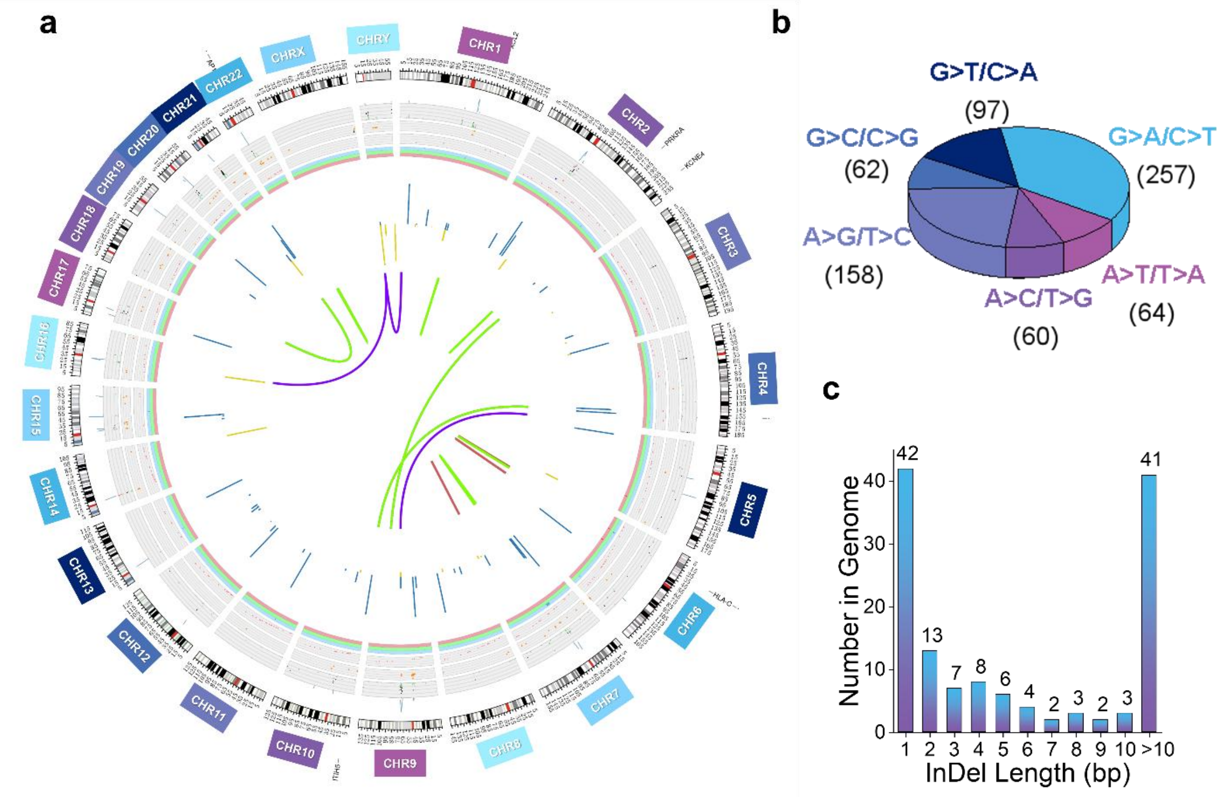

Supplementary Fig 30. (a) Circos plot summary of mutations between fresh blood samples and cryosilicified blood samples with 14-days storage (at 70 ^{o}C and 60% RH condition) in all human chromosomes (1-22+XY); (b) SNP mutations between fresh blood samples and cryosilicified blood samples with 14-days storage. (c) InDel mutations between fresh blood samples and cryosilicified blood samples with 14-days storage.

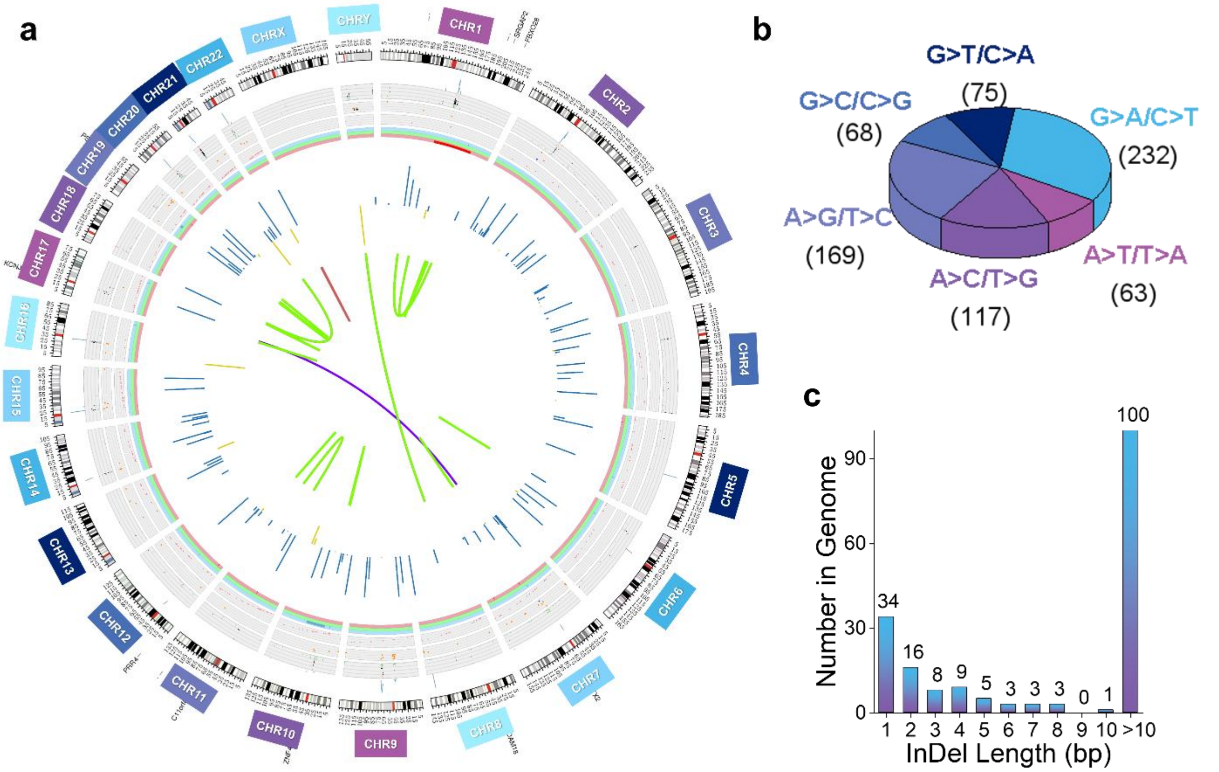

Supplementary Fig 31. (a) Circos plot summary of mutations between fresh blood samples and cryosilicified blood samples with 28-days storage (at 70 °C and 60% RH condition) in all human chromosomes (1-22+XY); (b) SNP mutations between fresh blood samples and cryosilicified blood samples with 28-days storage. (c) InDel mutations between fresh blood samples and cryosilicified blood samples with 28-days storage.

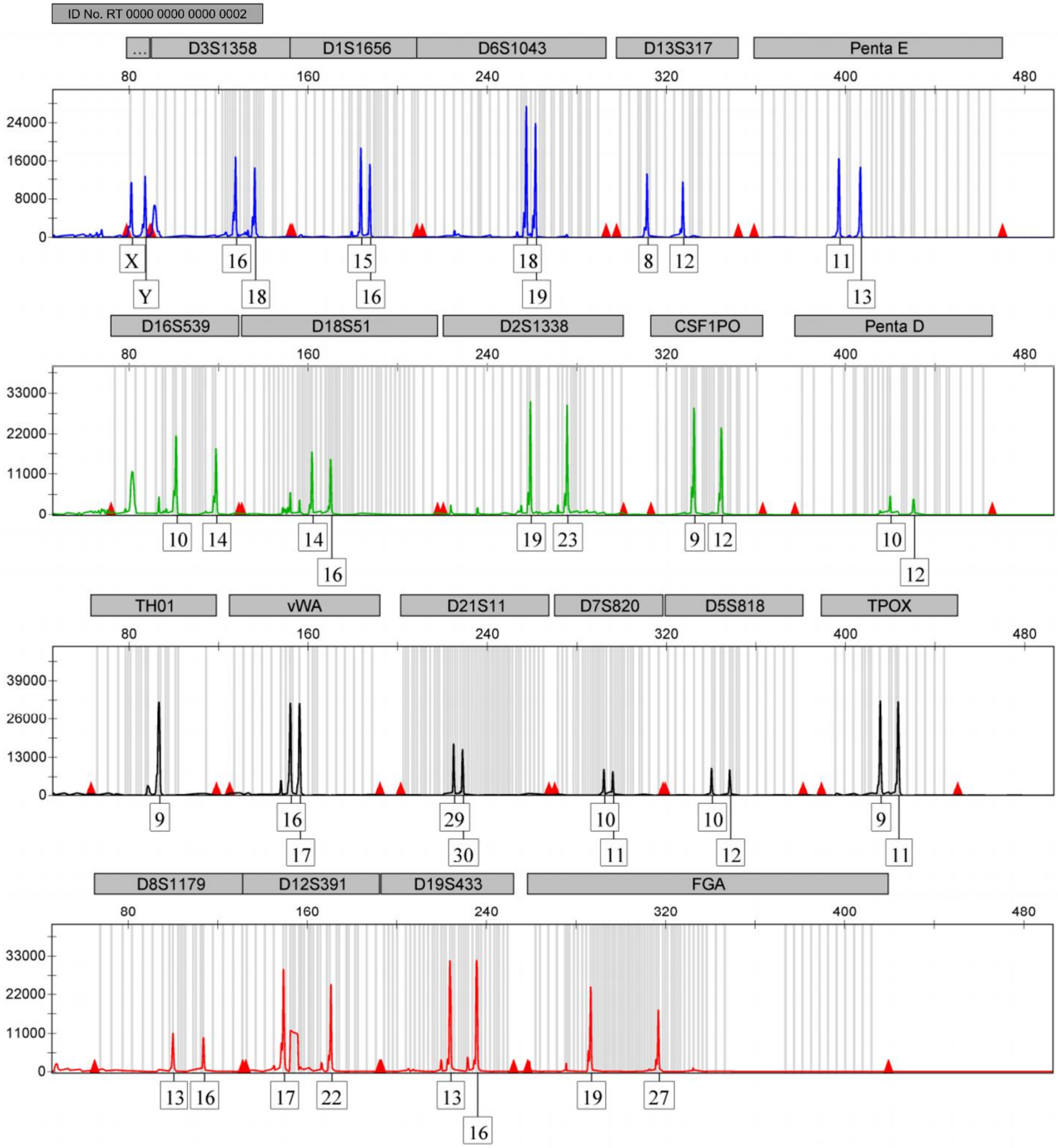

Supplementary Fig 32. STR analysis of extracted DNA from fresh blood sample.

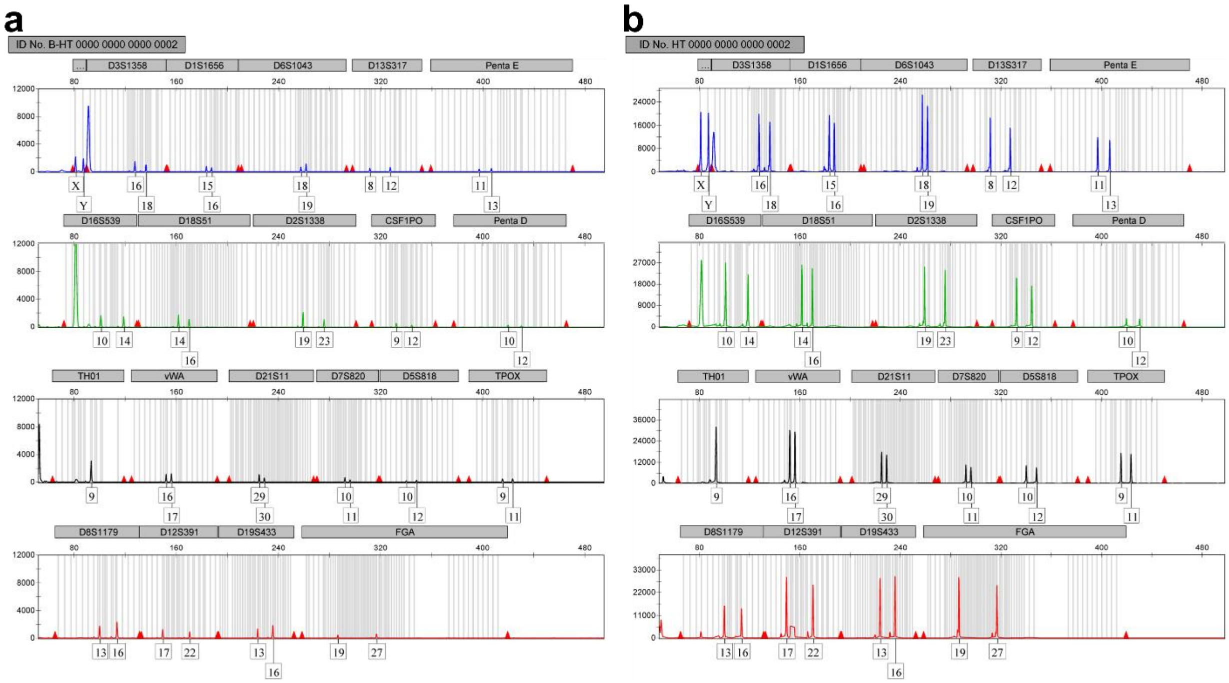

Supplementary Fig 33. STR analysis of extracted DNA from unprotected blood (a) and cryosilicified blood (b) samples after aging for 21 days at 70 ^{o}C and 60% RH.

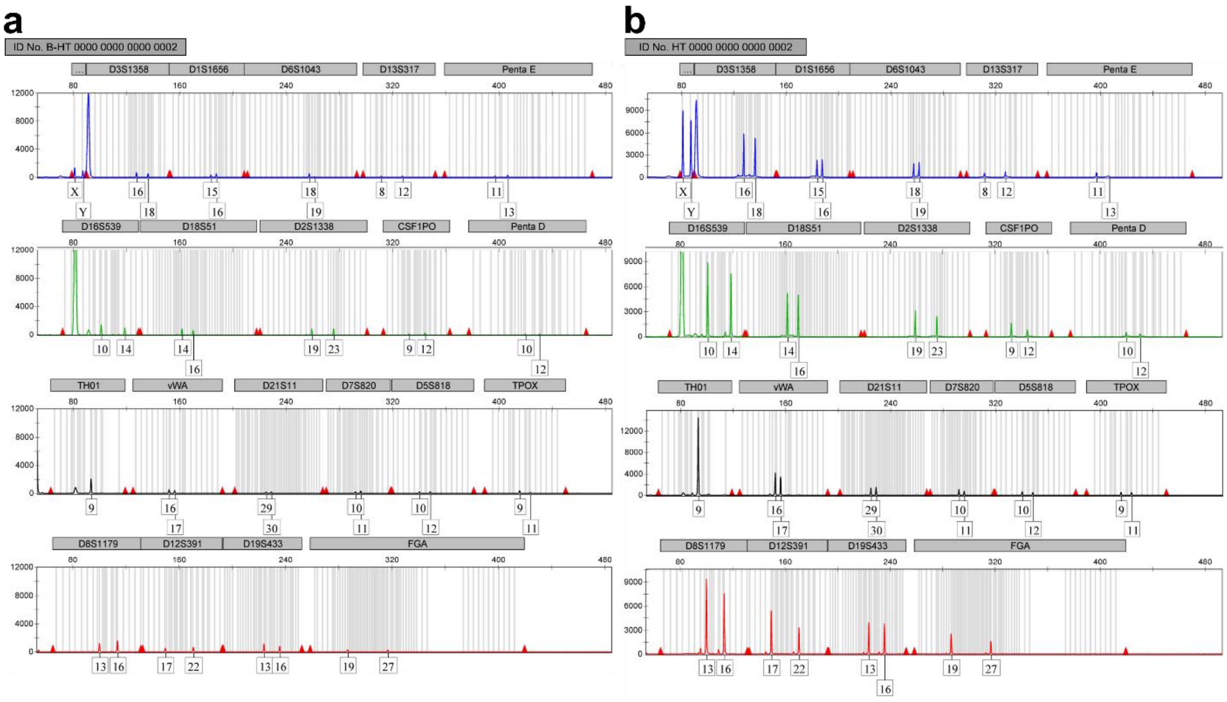

Supplementary Fig 34. STR analysis of extracted DNA from unprotected blood (a) and cryosilicified blood (b) samples after aging for 28 days at 70 ^{o}C and 60% RH.

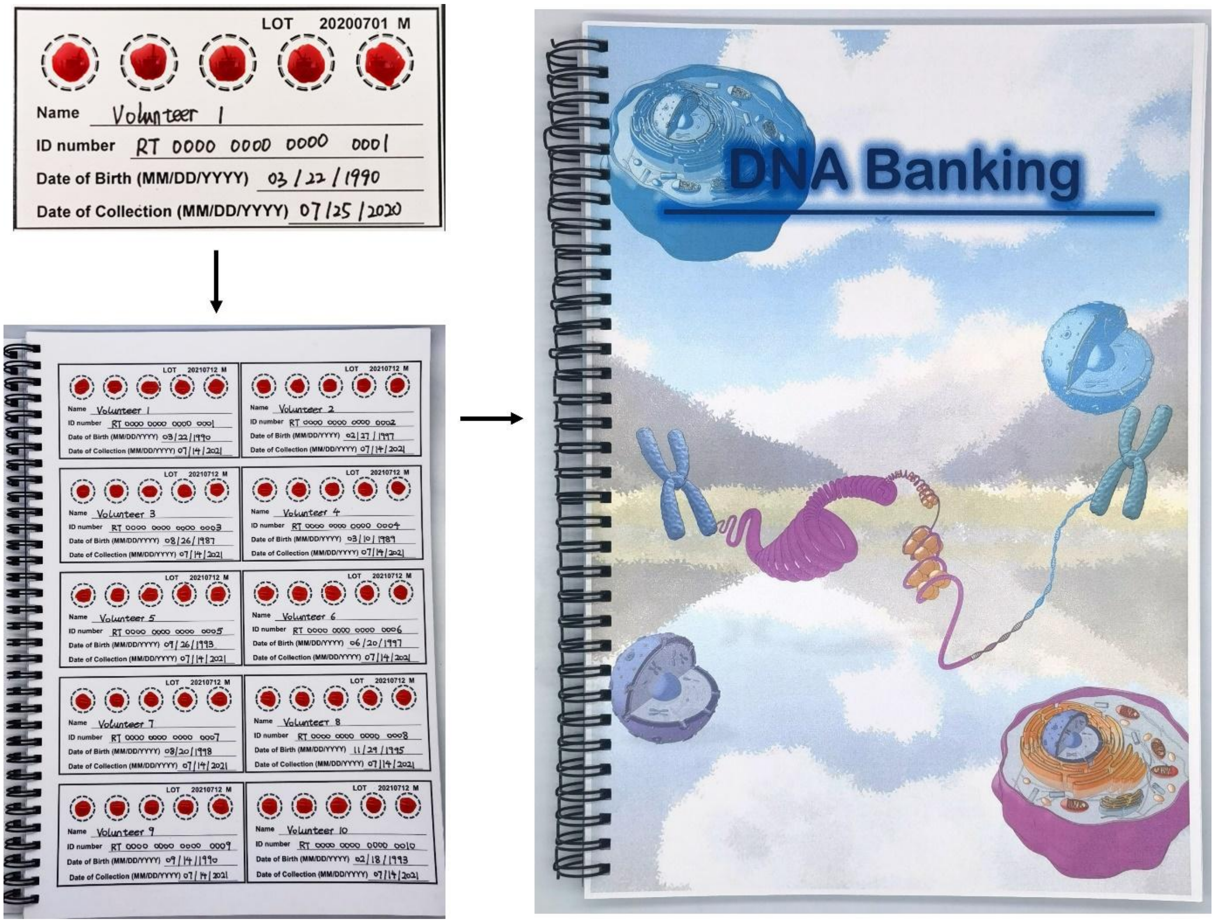

Supplementary Fig 35. Paper-based whole blood genomic DNA banking.

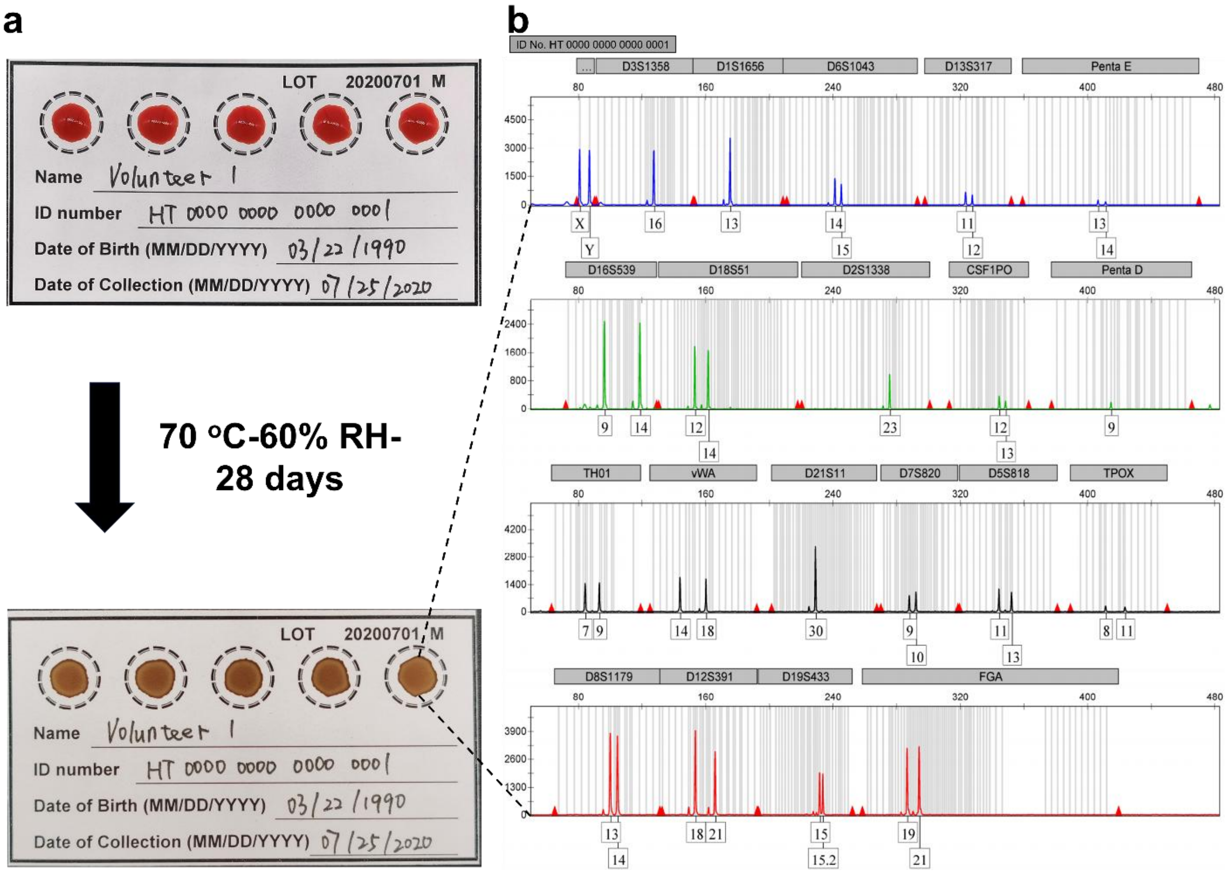

Supplementary Fig 36. (a) Blood card of Volunteer I, and (b) corresponding STR analysis of extracted DNA from the blood card after aging for 28 days at 70 °C and 60% RH.

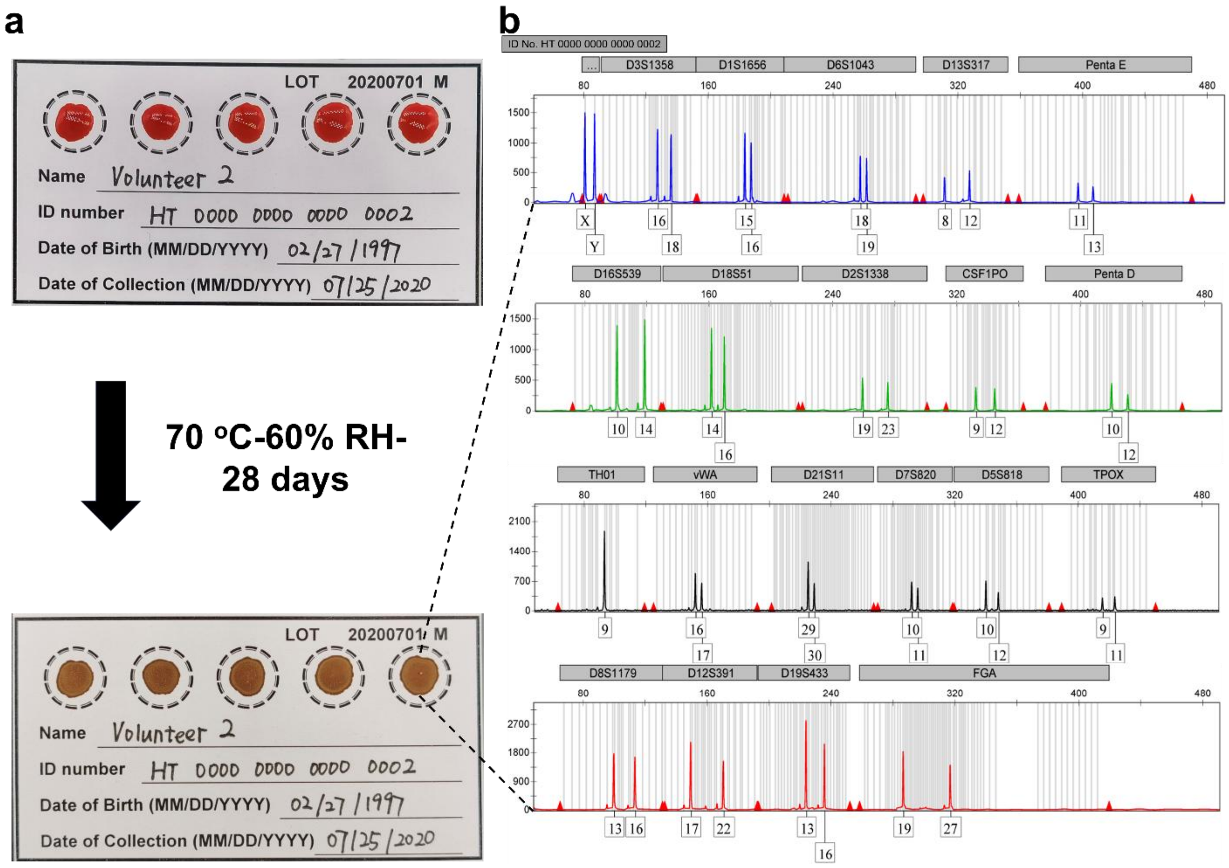

Supplementary Fig 37. (a) Blood card of Volunteer II, and (b) corresponding STR analysis of extracted DNA from the blood card after aging for 28 days at 70 °C and 60% RH.

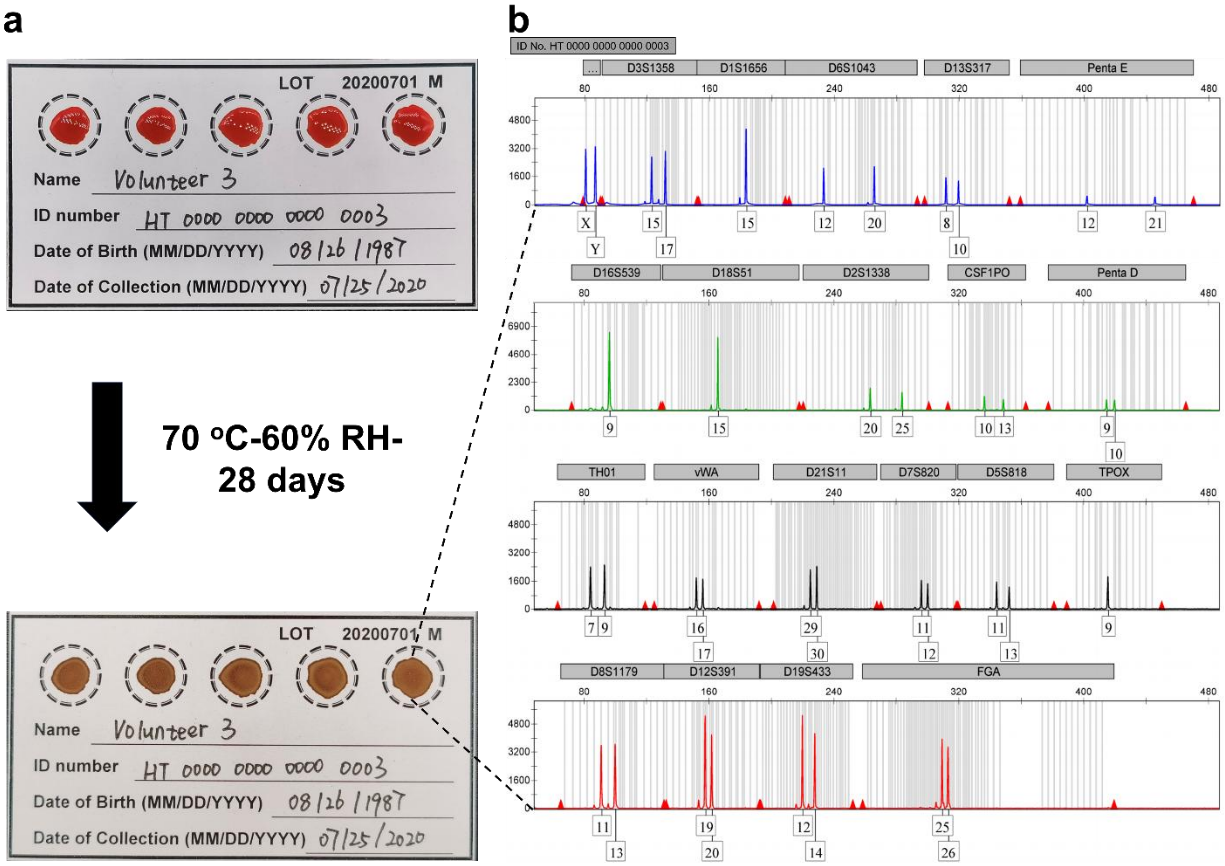

Supplementary Fig 38. (a) Blood card of Volunteer III, and (b) corresponding STR analysis of extracted DNA from the blood card after aging for 28 days at 70 °C and 60% RH.

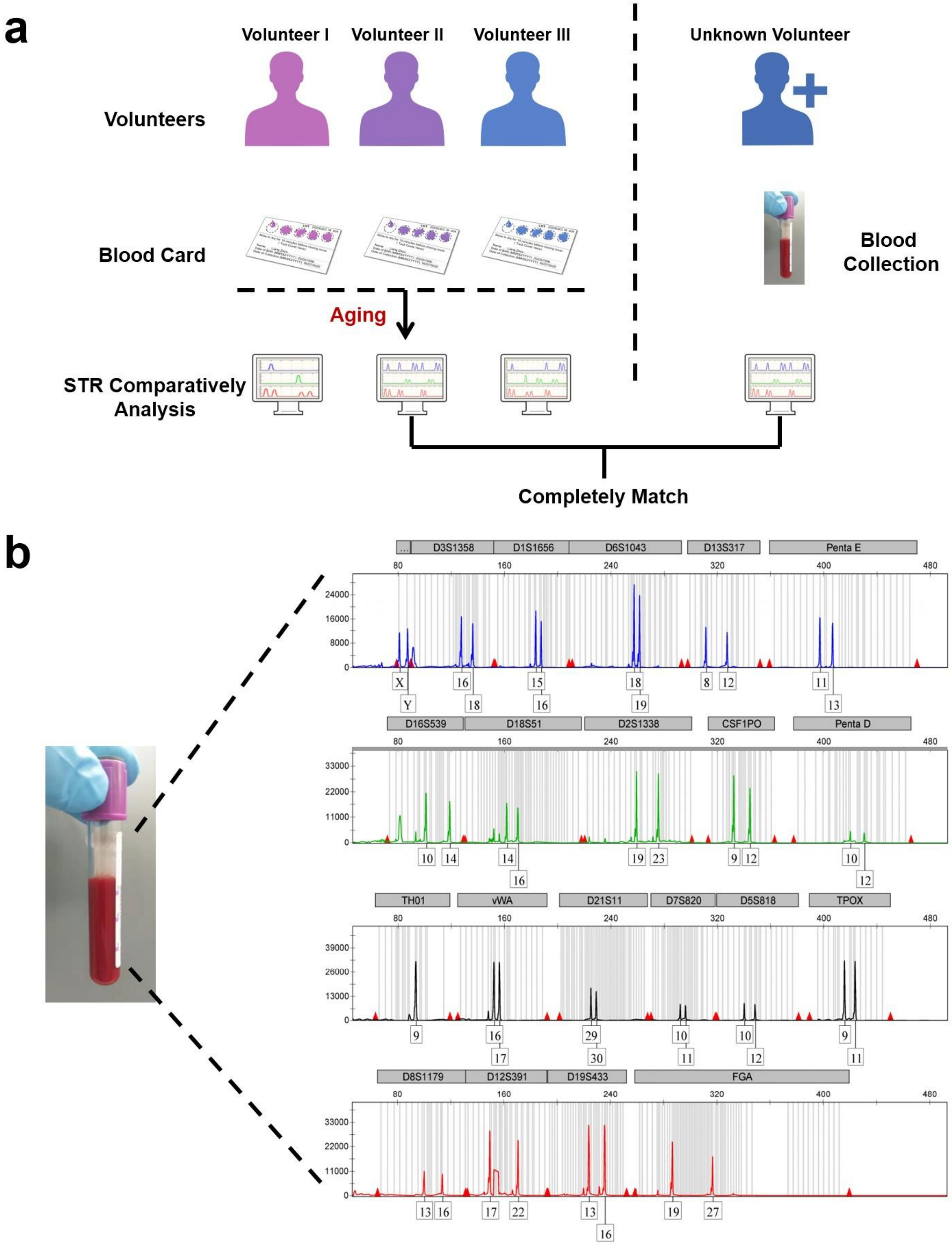

Supplementary Fig 39. (a) Schematic illustration of human identification process via the blood card. (b) STR analysis of extracted DNA from fresh blood sample from unidentified volunteer.

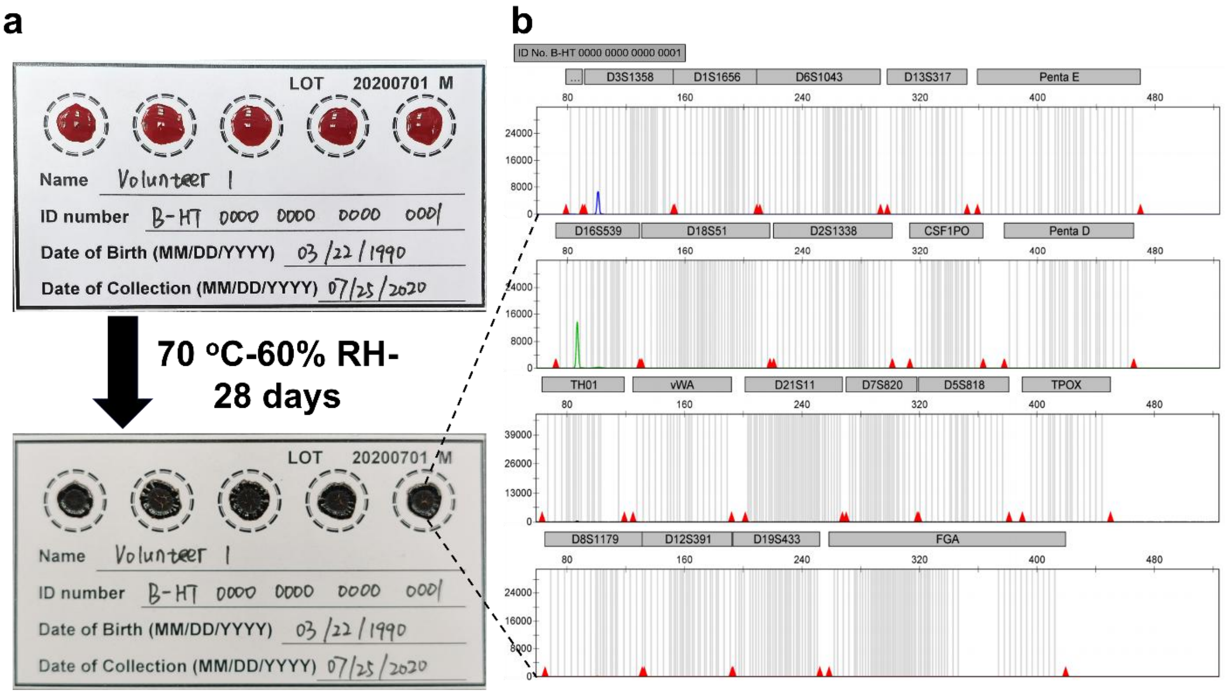

Supplementary Fig 40. (a) The blood card of Volunteer I constructed with unprotected blood sample, and (b) corresponding STR analysis of extracted DNA from the blood card after aging for 28 days at 70 °C and 60% RH.

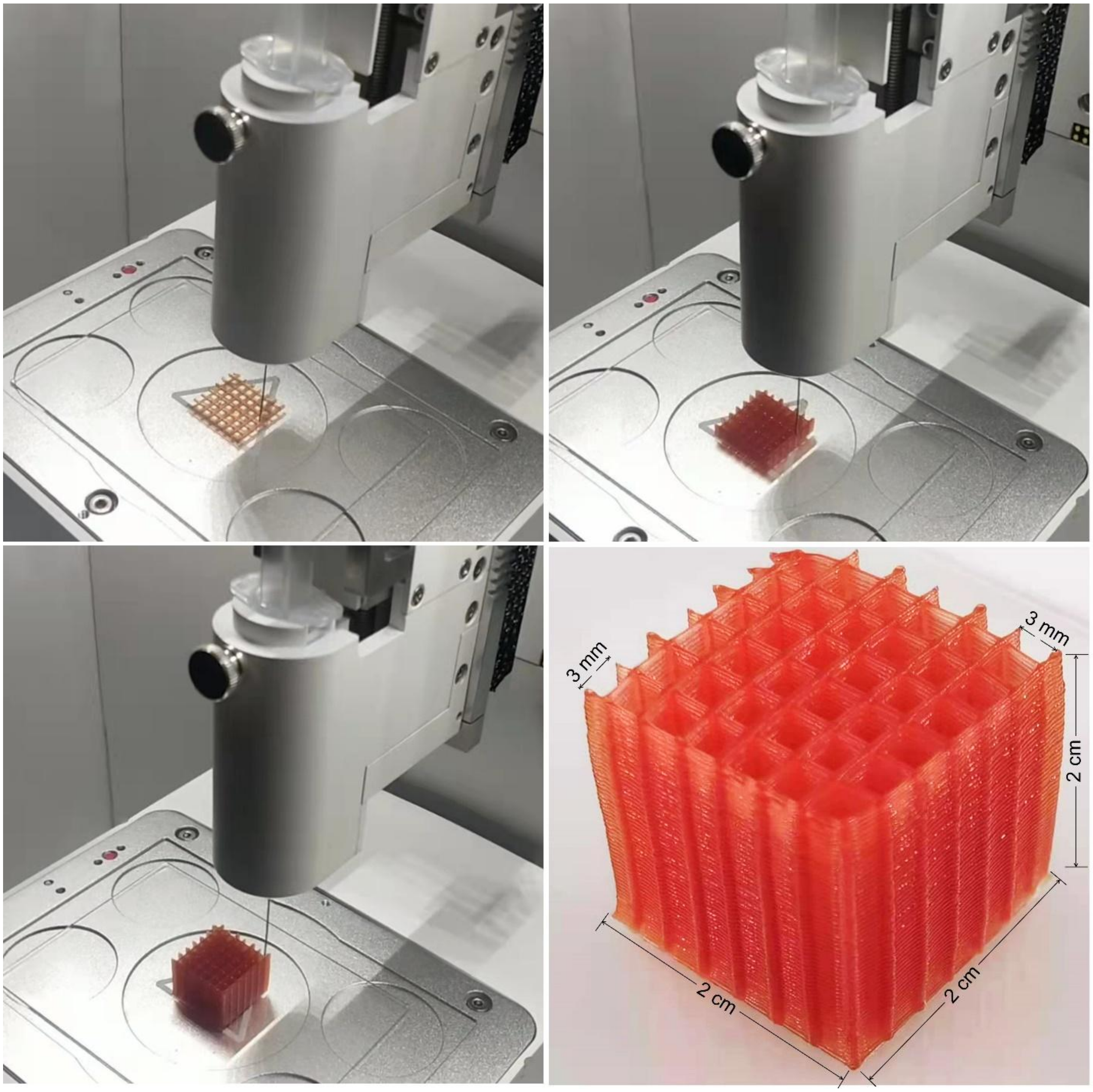

Supplementary Fig 41. 3D porous DNA banking scaffolds.

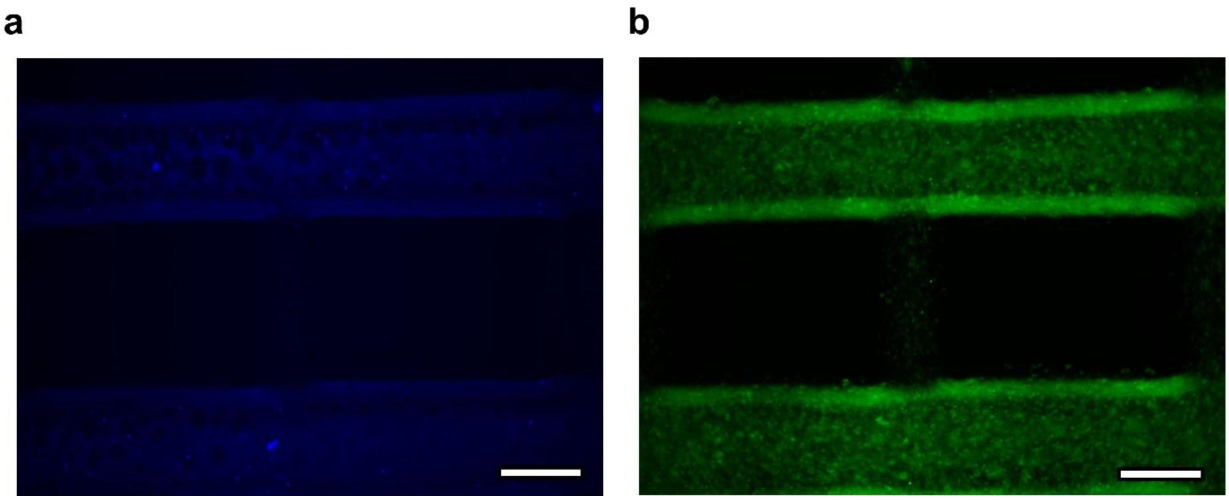

Supplementary Fig 42. Fluorescent microscope image of cryosilicified blood samples in 3D printing gel. (a) Cell nucleus was stained by Hoechst 33342 (blue), and (b) cell membrane was stained by DiO (green), Scale bar=200 μm. A representative image of three biological replicates is shown.

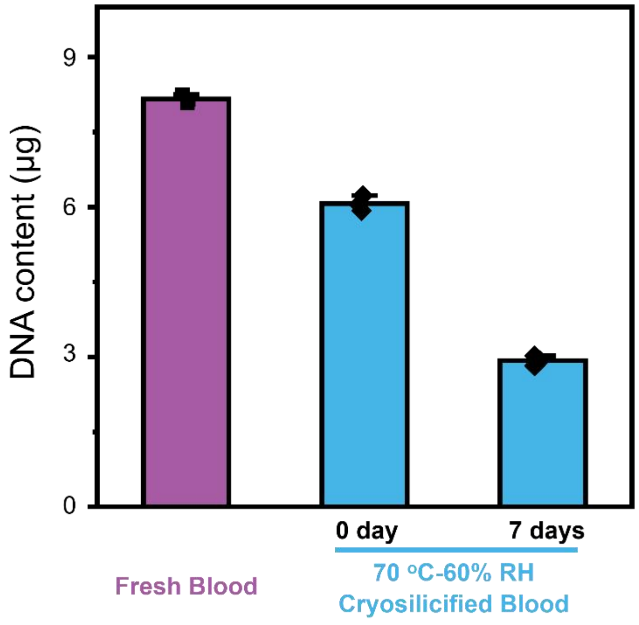

Supplementary Fig 43. The amount of extracted DNA from 3D porous DNA banking scaffolds (mean ± standard deviation, n = 3 biologically independent samples, two-tailed t-test)

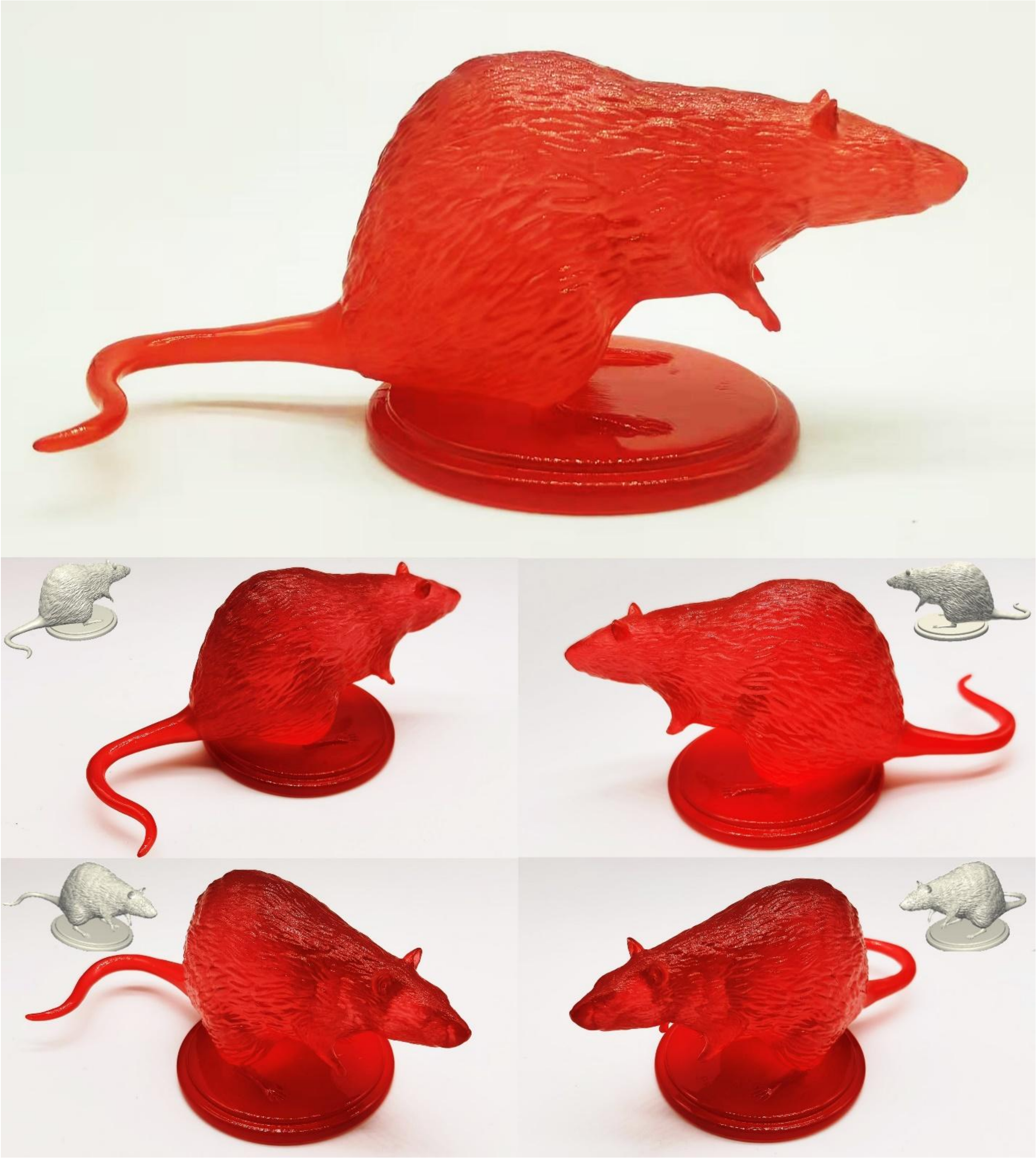

Supplementary Fig 44. 3D mouse figurine that stores its own 'genetic blueprint'.

Supplementary tables

Supplementary Table 1. Primer sequences information of PCR

Name	Forward primer (5′-3′)	Reverse primer (5′-3′)	Product length (bp)	Amplified region
G5	TGAAGCTCCCTGACGCCTAT	TCGCTGAGGGGACATGGTAT	181	Exon 12
G1	TGTGAGACCCCAGAGGAACT	TAAGATTGGGGCCTGGGAGAT	246	Exon 3
G4	ATTCTCTCCCTTGGCTTTCTCTC	TCCTCGGGGTTGAAGAACA	550	Exon 10 and most of Exon 11
G2	TCCCCAGCCACTTCTAACCA	GGCAACGGCAAGCCTTACAT	1072	Exon 4, 5 and 6
G3	TCTGGCTGGTTTTGAATGCG	TCCTTGGGTGTTGAGTTGGG	1730	Exon 7, 8, 9 and 10

Supplementary Table 2. Cycling parameters of PCR program

Step	Temperature	Time	Cycles
Predenaturation	95 °C	3 min	——
Denaturation	95 °C	30 s	
Annealing	61 °C	30 s	35cycles (G5, G1, G4)
Elongation	72 °C	60 s (G5, G1, G4)<br>120 s (G2, G3)	40cycles (G2, G3)
Final elongation	72 °C	10 min	——

Supplementary Table 3. STR loci information

Number	Marker	Chromosomal location	Repeat Structure
1	Amelogenin(Y/X)	(X;Y)(**p22.33;p11.32**)	
2	D3S1358	Chr 3; **3p21.31**	[AGAT], [TCTA]
3	D1S1656	Chr 1; **1q42**	$[TAGA]_n[TGA]_{0-1}[TAGA]_n[TAGG]_{0-1}[TG]_5$
4	D6S1043	Chr 6; **6q15**	$[AGAT]_{9-25}$
5	D13S317	Chr 13; **13q31.1**	[TATC]
6	Penta E	Chr 15; **15q26.2**	[AAAGA]
7	D16S539	Chr 16; **16q24.1**	[GATA]
8	D18S51	Chr 18; **18q21.33**	[GAAA]
9	D2S1338	Chr 2; **2q35**	$[TGCC]_n[TTCC]_n$
10	CSF1PO	Chr 5; **5q33.1**	[AGAT]
11	Penta D	Chr 21; **21q22.3**	[AAAGA]
12	TH01	Chr 11; **11p15.5**	[AATG]
13	vWA	Chr 12; **12p13.31**	[TCTA], [TCTG], [TCCA]
14	D21S11	Chr 21; **21q21.1**	[TCTA], [TCTG]
15	D7S820	Chr 7; **7q21.11**	[GATA]
16	D5S818	Chr 5; **5q23.2**	[AGAT]
17	TPOX	Chr 2; **2p25.3**	[AATG]
18	D8S1179	Chr 8; **8q24.13**	[TATC]
19	D12S391	Chr 12;	$[AGAT]_{8-17}[AGAC]_{6-10}[AGAT]_{0-1}$
20	D19S433	Chr 19; **19q12**	$(AAGG)(AAAG)(AAGG)(TAGG)[AAGG]_n$
21	FGA	Chr 4; **4q28**	$[TTTC]_3TTTTTTCT[CTTT]_nCTCC[TTCC]_2$

Supplementary Table 4. Linear fit of concentration data to first order decay rate expression

Samples	70 °C		75 °C		80 °C	
	k (s^{-1})	R^2	k (s^{-1})	R^2	k (s^{-1})	R^2
Purified DNA	$2.34 \cdot 10^{-6}$	0.97(3)	$3.45 \cdot 10^{-6}$	0.97(3)	$6.57 \cdot 10^{-6}$	1(2)
Unprotected Blood	$1.54 \cdot 10^{-6}$	0.88(4)	$2.39 \cdot 10^{-6}$	0.97(3)	$4.33 \cdot 10^{-6}$	1(2)
4 °C Silicified Blood	$8.03 \cdot 10^{-7}$	0.78(5)	$1.29 \cdot 10^{-6}$	0.88(4)	$2.30 \cdot 10^{-6}$	0.88(3)
Cryosilicified Blood	$2.13 \cdot 10^{-7}$	0.94(5)	$8.27 \cdot 10^{-7}$	0.99(5)	$1.04 \cdot 10^{-6}$	0.99(5)

Supplementary Table 5. Linear fit of rate data to yield Arrhenius activation energies ($\ln(k_T)$ vs. $1/T$).

Samples	E_A (KJ/mol)	k_0 (s^{-1})	R^2
Purified DNA	103.79	$1.43 \cdot 10^{10}$	0.95
Unprotected Blood	103.98	$1.03 \cdot 10^{10}$	0.98
4 °C Silicified Blood	105.87	$1.05 \cdot 10^{10}$	0.99
Cryosilicified Blood	160.16	$6.28 \cdot 10^{17}$	0.99

Supplementary Table 6. WGS information of untreated fresh blood and cryosilicified blood without storage.

Name	Coverage Rate (%) (>=1X)	Coverage Rate (%) (>=4X)	SNP （bp）	InDel （num）
	**WGS information**			
**Fresh Blood**	99.21	95.56		
**Cryosilicified Blood-0 day**	99.33	96.15	831	95

*Reference sequence: The hG19 assembly version of the human genome (download from UCSC database)

Supplementary Table 7. WGS information of untreated fresh blood and cryosilicified blood with UV irradiation or 30% H_2O_2 treatment.

WGS information

Name	Coverage Rate (%) (>=1X)	Coverage Rate (%) (>=4X)	SNP （bp）	InDel （num）
**Fresh Blood**	99.78	97.86		
**Cryosilicified blood-UV-16 h**	99.72	96.77	617	514
**Cryosilicified blood-H_2O_2 -30%-4 h**	99.75	97.01	535	338

*Reference sequence: The hg19 assembly version of the human genome (download from UCSC database)

Supplementary Table 8. WGS information of untreated fresh blood and cryosilicified blood with 7-, 14-, 28-days storage at 70 ^{o}C and 60% RH condition.

WGS information				
**Name**	**Coverage Rate (%) (>=1X)**	**Coverage Rate (%) (>=4X)**	**SNP （bp）**	**InDel （num）**
**Fresh Blood**	99.78	97.86		
**Cryosilicified Blood-7 days**	99.79	98.13	670	92
**Cryosilicified Blood-14 days**	99.81	98.35	698	131
**Cryosilicified Blood-28 days**	99.79	98.06	724	182

*Reference sequence: The hg19 assembly version of the human genome (download from UCSC database)

Supplementary Table 9. Simulate the half-life of different cities

City	Country	Annual average temperature (°C)*	Half life (years)
New Delhi	The Republic of India	25.1	393
Phoenix, AZ	United States of America	23.4	569
Guangzhou	The People's Republic of China	21.8	809
Cairo	The Arab Republic of Egypt	21.4	884
Brasilia	The Federative Republic of Brazil	21.2	924
Lima	The Republic of Peru	18.7	1619
Antananarivo	The Republic of Madagascar	18.5	1694
Sydney	Commonwealth of Australia	18.2	1813
Los Angeles, CA	United States of America	17.2	2277
Cape Town	The Republic of South Africa	16.0	2999
ShangHai	The People's Republic of China	15.8	3140
Tokyo	Japan	15.6	3289
Bilbao	The Kingdom of Spain	15.5	3366
Washington D.C.	United States of America	14.5	4244
Albuquerque, NM	United States of America	13.4	5489
Canberra	Commonwealth of Australia	13.0	6029
Wellington	New Zealand	12.8	6320
Moscow	Russian Federation	12.5	6783
Santiago de Chile	Republic of Chile	12.5	6783
Beijing	The People's Republic of China	11.8	8005
Paris	French Republic	10.0	12304
London	The United Kingdom of Great Britain and Northern Ireland	9.5	13877
Berlin	Federal Republic of Germany	8.9	16042
Guben	Federal Republic of Germany	8.7	16839
Copenhagen	The Kingdom of Denmark	8.0	19963
Stockholm	The Kingdom of Sweden	6.6	28128
Ottawa	Canada	5.8	34269
Oslo	The Kingdom of Norway	5.7	35128
Helsinki	The Republic of Finland	4.5	47350
Reykjavík	The Republic of Iceland	4.3	49778
Harbin	The People's Republic of China	3.5	60846
Amundsen-Scott South Pole Station	United States of America	-49.3	8×10^{11}

*Annual average temperature data is from China Meteorological Data Service Centre http://data.cma.cn/analysis/globalClimate.html

Supplementary Table 10. STR loci information of Volunteer I, Volunteer II, Volunteer III and the unknown Volunteer.

Number	Marker	Loci							
		Volunteer I		Volunteer II		Volunteer III		Volunteer ?	
1	Amelogenin (Y/X)	X	Y	X	Y	X	Y	X	Y
2	D3S1358	16	16	16	18	15	17	16	18
3	D1S1656	9	9	15	16	15	15	15	16
4	D6S1043	12	13	18	19	12	20	18	19
5	D13S317	13	13	8	12	8	10	8	12
6	Penta E	14	15	11	13	12	21	11	13
7	D16S539	11	12	10	14	9	9	10	14
8	D18S51	13	14	14	16	15	15	14	16
9	D2S1338	8	11	19	23	20	25	19	23
10	CSF1PO	11	13	9	12	10	13	9	12
11	Penta D	9	10	10	12	9	10	10	12
12	TH01	30	30	9	9	7	9	9	9
13	vWA	14	18	16	17	16	17	16	17
14	D21S11	7	9	29	30	29	30	29	30
15	D7S820	9	14	10	11	11	12	10	11
16	D5S818	12	14	10	12	11	13	10	12
17	TPOX	23	23	9	11	9	9	9	11
18	D8S1179	13	14	13	16	11	13	13	16
19	D12S391	18	21	17	22	19	20	17	22
20	D19S433	15	15.2	13	16	12	14	13	16
21	FGA	19	21	19	27	25	26	19	27
