# Supplementary material for: Long-term whole blood DNA preservation by cost-efficient cryosilicification
Source: Nat Commun. 2022 Oct 21;13:6265. doi: 10.1038/s41467-022-33759-y (PMC9587218; doi:10.1038/s41467-022-33759-y)
Supplement: Supplementary file 3 — Reporting Summary [file 41467_2022_33759_MOESM3_ESM.pdf]

nature portfolio

Corresponding author(s): Stefan Wuttke and Wei Zhu

Last updated by author(s): Sep 29, 2022

Reporting Summary

Nature Portfolio wishes to improve the reproducibility of the work that we publish. This form provides structure for consistency and transparency in reporting. For further information on Nature Portfolio policies, see our Editorial Policies and the Editorial Policy Checklist.

Statistics

For all statistical analyses, confirm that the following items are present in the figure legend, table legend, main text, or Methods section.

n/a	Confirmed	
☐	☒	The exact sample size (*n*) for each experimental group/condition, given as a discrete number and unit of measurement
☐	☒	A statement on whether measurements were taken from distinct samples or whether the same sample was measured repeatedly
☐	☒	The statistical test(s) used AND whether they are one- or two-sided<br>*Only common tests should be described solely by name; describe more complex techniques in the Methods section.*
☐	☒	A description of all covariates tested
☐	☒	A description of any assumptions or corrections, such as tests of normality and adjustment for multiple comparisons
☐	☒	A full description of the statistical parameters including central tendency (e.g. means) or other basic estimates (e.g. regression coefficient) AND variation (e.g. standard deviation) or associated estimates of uncertainty (e.g. confidence intervals)
☐	☒	For null hypothesis testing, the test statistic (e.g. *F*, *t*, *r*) with confidence intervals, effect sizes, degrees of freedom and *P* value noted<br>*Give P values as exact values whenever suitable.*
☒	☐	For Bayesian analysis, information on the choice of priors and Markov chain Monte Carlo settings
☒	☐	For hierarchical and complex designs, identification of the appropriate level for tests and full reporting of outcomes
☒	☐	Estimates of effect sizes (e.g. Cohen's *d*, Pearson's *r*), indicating how they were calculated

Our web collection on statistics for biologists contains articles on many of the points above.

Software and code

Policy information about availability of computer code

Data collection	No software was written for this project.
Data analysis	No software was written for this project.

For manuscripts utilizing custom algorithms or software that are central to the research but not yet described in published literature, software must be made available to editors and reviewers. We strongly encourage code deposition in a community repository (e.g. GitHub). See the Nature Portfolio guidelines for submitting code & software for further information.

Data

Policy information about availability of data

All manuscripts must include a data availability statement. This statement should provide the following information, where applicable:

- Accession codes, unique identifiers, or web links for publicly available datasets
- A description of any restrictions on data availability
- For clinical datasets or third party data, please ensure that the statement adheres to our policy

The source data underlying Figs. 2f, g, i, l, m, 3a, b, d, e, 4a-c, d, 5a-c, 6g and Supplementary Figs. 1, 2, 8, 10, 12-31, 32-34, 36-38, 40 and 43 are provided as a Source Data file. The Whole genomic sequencing data are available at NCBI under Project PRJNA878673 [http://www.ncbi.nlm.nih.gov/bioproject/878673].

Human research participants

Policy information about studies involving human research participants and Sex and Gender in Research.

Reporting on sex and gender	The data of sex and gender was not collected. This information will not affect the preservation of gene information in this project.
Population characteristics	This study included 10 participants (mean age 27.8±3.37 years), which are 6 males (mean age 28.5±3.73 years) and 4 females (mean age 26.75±2.38 years).
Recruitment	10 participants were recruited between Oct 2019 and May 2022. All the participants understand and accept in detail of the inspections and information to be provided. All participants' written consents were obtained. All participants met the following inclusion criteria: (1) age 18- 40 years, Han Chinese; (2) at least 50kg; (3) generally healthy by self-report. The exclusion criteria were as follows: (1) pregnancy or lactation; (2) cold and flu symptoms the day of collection; (3) infections within two weeks prior to collection; (4) symptoms of a heart condition within the six months prior to collection.
Ethics oversight	The study protocol was approved by the ethics committee, Guangzhou Women and Children's Medical Center, Guangzhou, China (Protocol 201928300).

Note that full information on the approval of the study protocol must also be provided in the manuscript.

Field-specific reporting

Please select the one below that is the best fit for your research. If you are not sure, read the appropriate sections before making your selection.

- [x] Life sciences
- [ ] Behavioural & social sciences
- [ ] Ecological, evolutionary & environmental sciences

For a reference copy of the document with all sections, see nature.com/documents/nr-reporting-summary-flat.pdf

Life sciences study design

All studies must disclose on these points even when the disclosure is negative.

Sample size	All experiment was preformed with samples size more than 3 except whole genomic sequencing experiment. The preservation outcomes were distinct with a sample size of 3.
Data exclusions	No data was excluded.
Replication	All studies were replicated as needed to validate significance and novel findings.
Randomization	The volunteers have been randomly chosen to participate in the study.
Blinding	All the samples were labeled from "A" to "D" for the aging study.

Reporting for specific materials, systems and methods

We require information from authors about some types of materials, experimental systems and methods used in many studies. Here, indicate whether each material, system or method listed is relevant to your study. If you are not sure if a list item applies to your research, read the appropriate section before selecting a response.

Materials & experimental systems

n/a	Involved in the study	
x		Antibodies
x		Eukaryotic cell lines
x		Palaeontology and archaeology
	x	Animals and other organisms
x		Clinical data
x		Dual use research of concern

Methods

n/a	Involved in the study	
x		ChIP-seq
	x	Flow cytometry
x		MRI-based neuroimaging

Animals and other research organisms

Policy information about studies involving animals; ARRIVE guidelines recommended for reporting animal research, and Sex and Gender in Research

Laboratory animals	Male BALB/C mice aged 5-6 weeks were used in this study.
Wild animals	No field animals were used in this study
Reporting on sex	*Indicate if findings apply to only one sex; describe whether sex was considered in study design, methods used for assigning sex. Provide data disaggregated for sex where this information has been collected in the source data as appropriate; provide overall numbers in this Reporting Summary. Please state if this information has not been collected. Report sex-based analyses where performed, justify reasons for lack of sex-based analysis.*
Field-collected samples	*For laboratory work with field-collected samples, describe all relevant parameters such as housing, maintenance, temperature, photoperiod and end-of-experiment protocol OR state that the study did not involve samples collected from the field.*
Ethics oversight	All animal protocols were approved by South China University of Technology Institutional Animal Care and Use Committee.

Note that full information on the approval of the study protocol must also be provided in the manuscript.

Flow Cytometry

Plots

Confirm that:

- [ ] The axis labels state the marker and fluorochrome used (e.g. CD4-FITC).
- [x] The axis scales are clearly visible. Include numbers along axes only for bottom left plot of group (a 'group' is an analysis of identical markers).
- [x] All plots are contour plots with outliers or pseudocolor plots.
- [ ] A numerical value for number of cells or percentage (with statistics) is provided.

Methodology

Sample preparation	All blood samples were collected by experienced phlebotomists and stored in KWS vacutainer blood collection tubes (KWSMedical, Shijiazhuang, China) containing 1.5 mg of EDTA per mL of blood for anticoagulation purposes.
Instrument	BD Accuri C6 plus flow cytometer
Software	Flowjo v10
Cell population abundance	The purification of whole blood was carried out using Ficoll density gradient centrifugation procedure. Then, collection of erythrocytes and leukocytes and these cells were diluted by 1 x PBS, and 1×10^5 cells were analyzed. Total blood cells were diluted directly by 1 x PBS, and 1×10^5 cells were analyzed.
Gating strategy	All blood cells are first gated on the basis of their scattering properties using forward(FSC)and side scatter (SSC).

- [x] Tick this box to confirm that a figure exemplifying the gating strategy is provided in the Supplementary Information.
