# Supplementary figures and images for: Long-term whole blood DNA preservation by cost-efficient cryosilicification
Source: Nat Commun. 2022 Oct 21;13:6265. doi: 10.1038/s41467-022-33759-y (PMC9587218; doi:10.1038/s41467-022-33759-y)

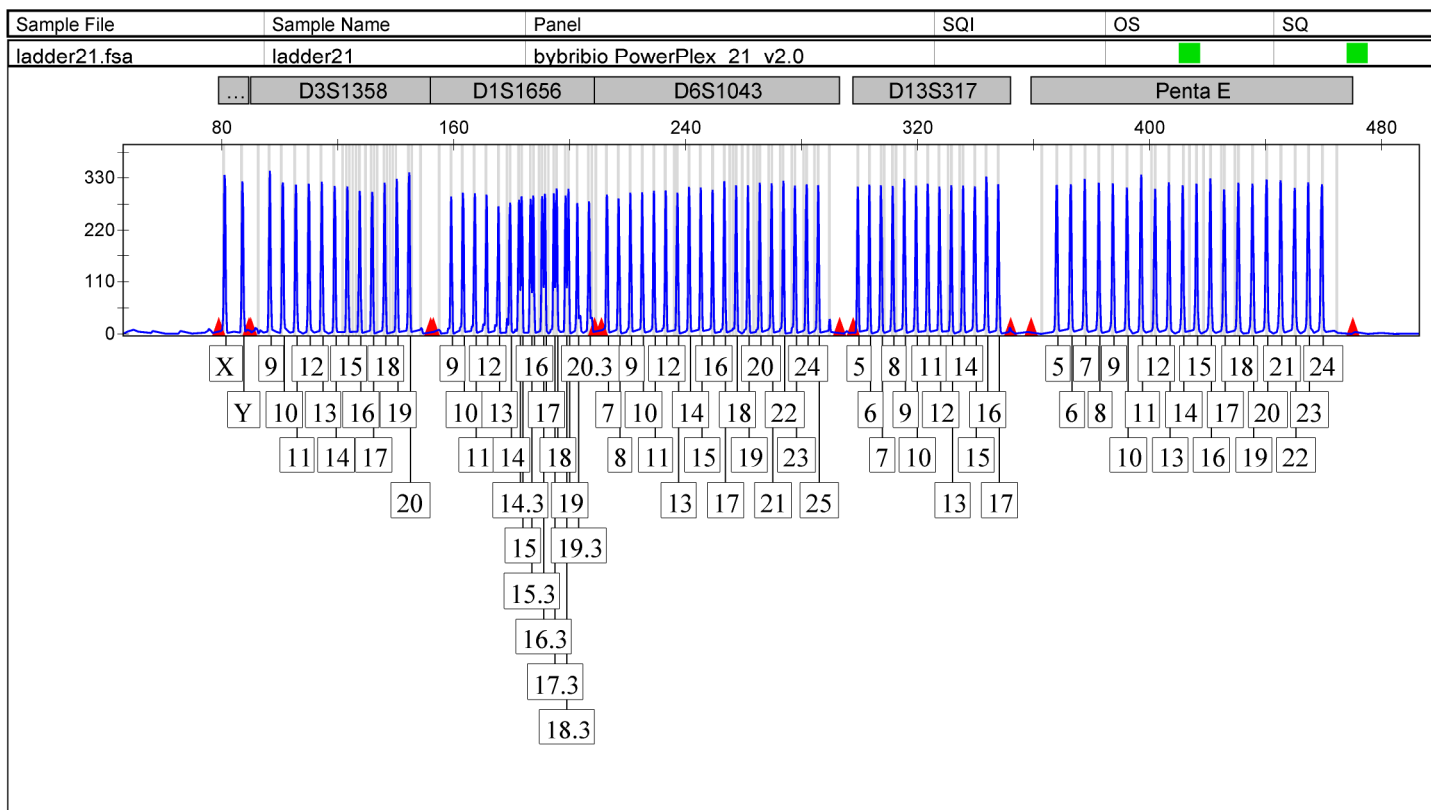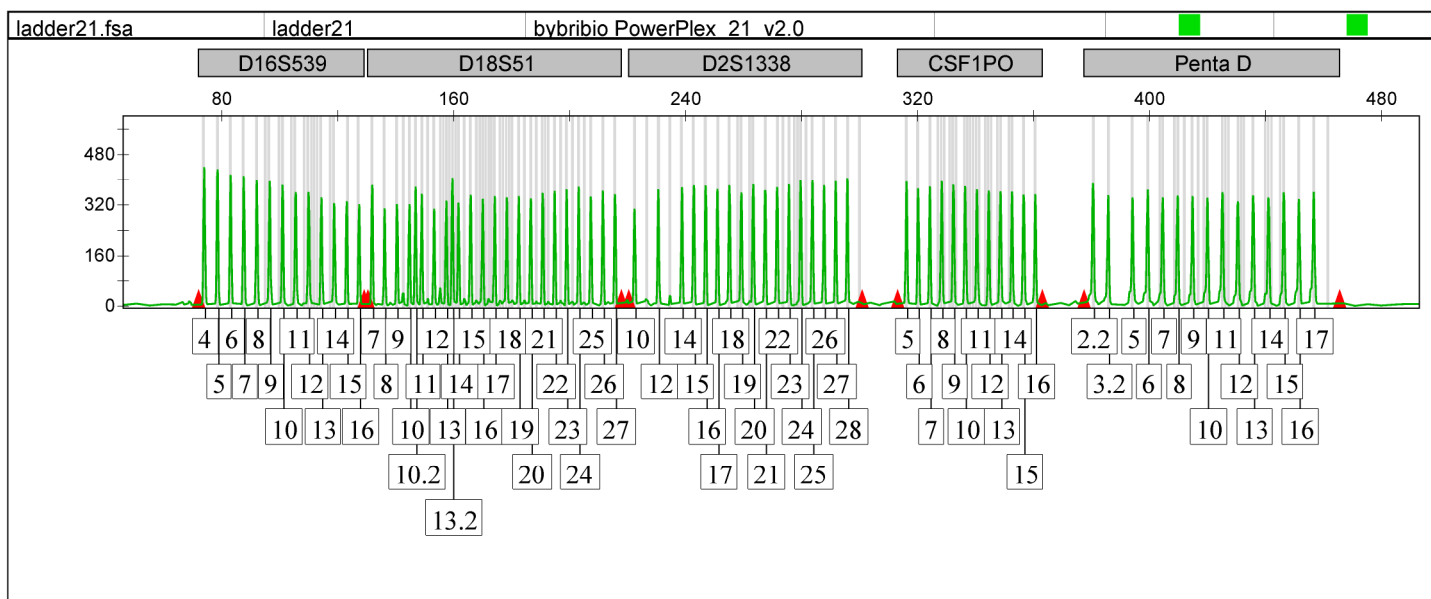

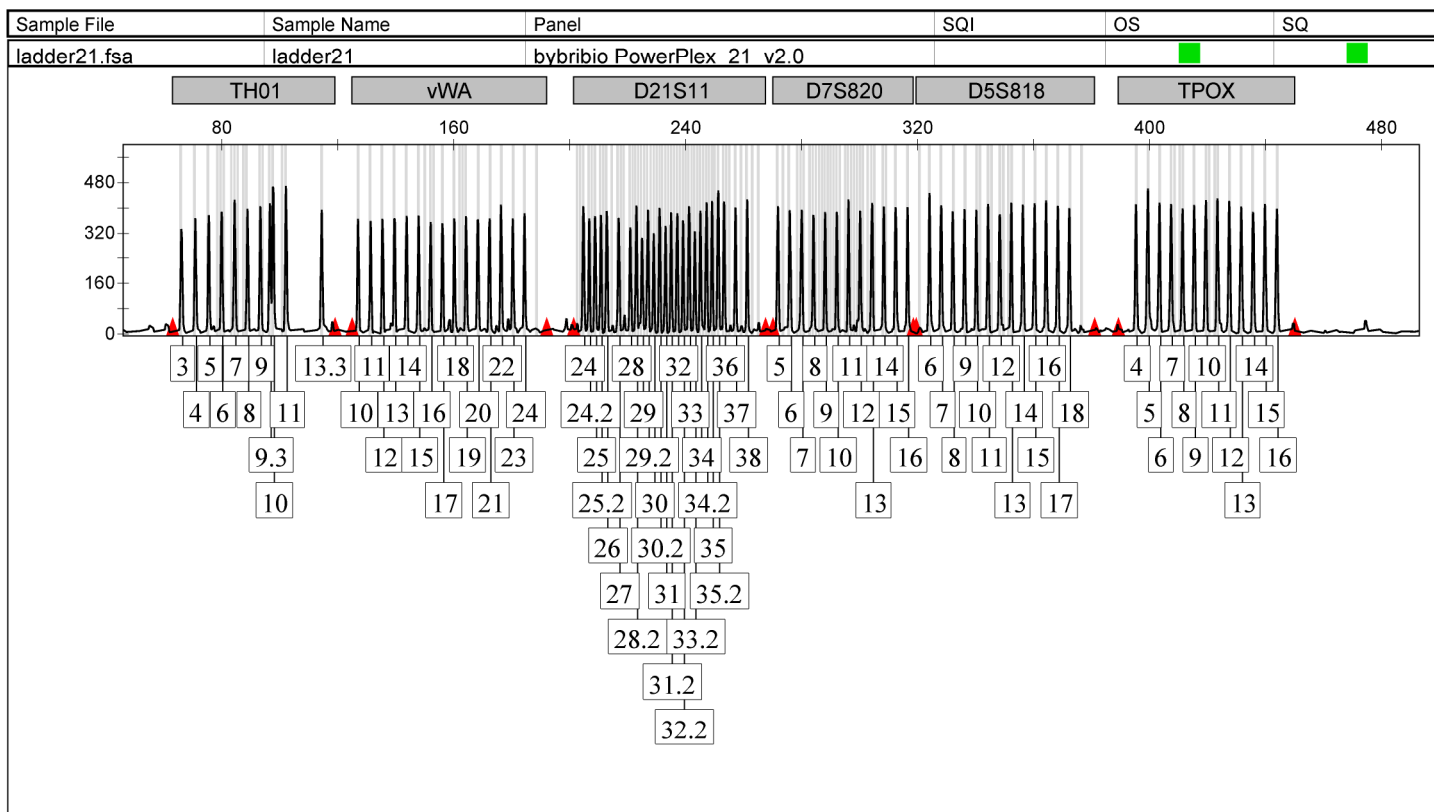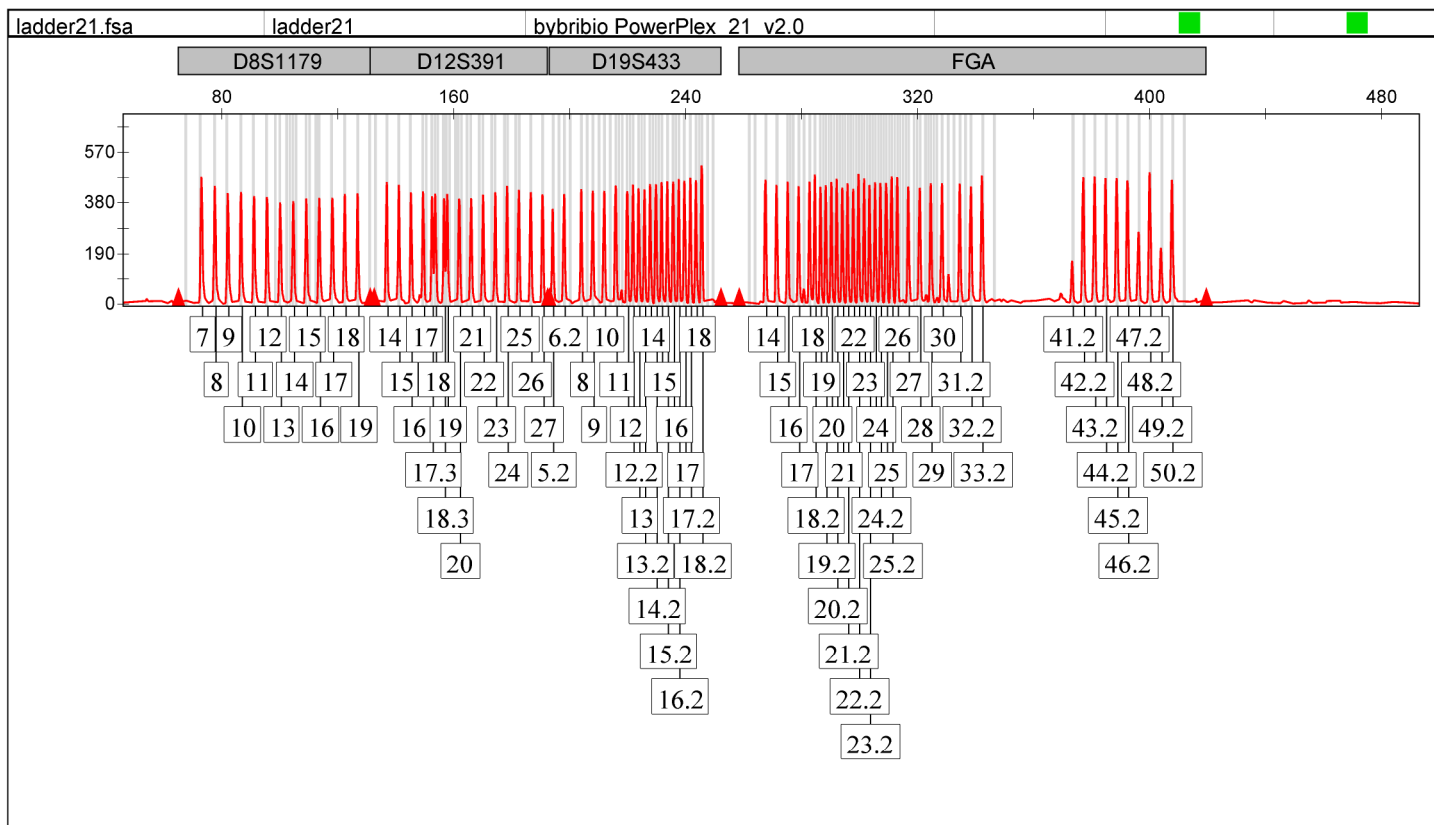

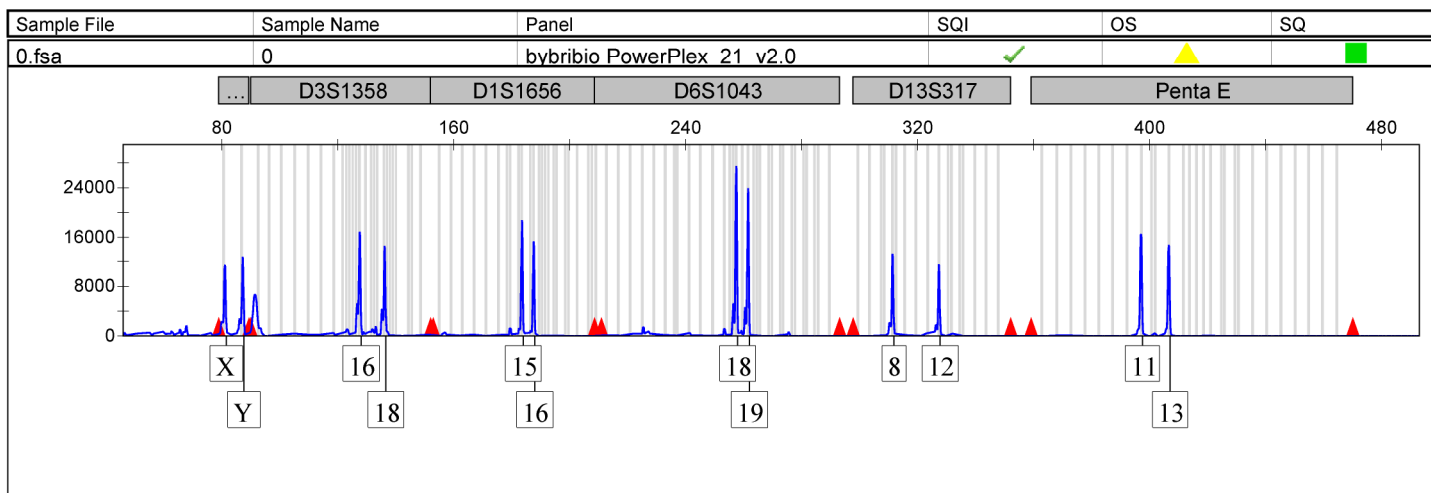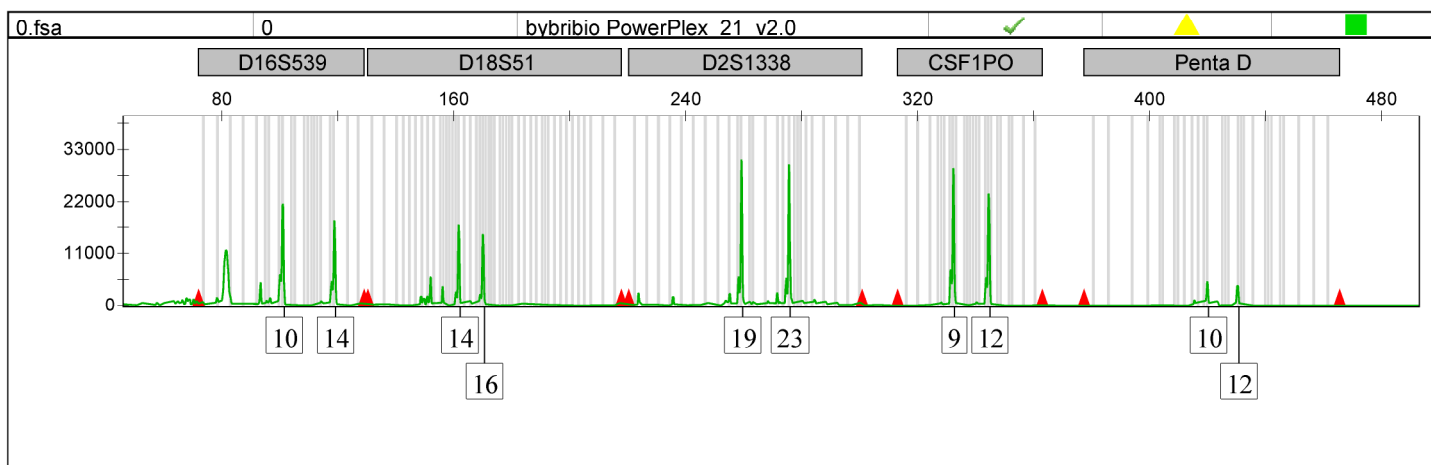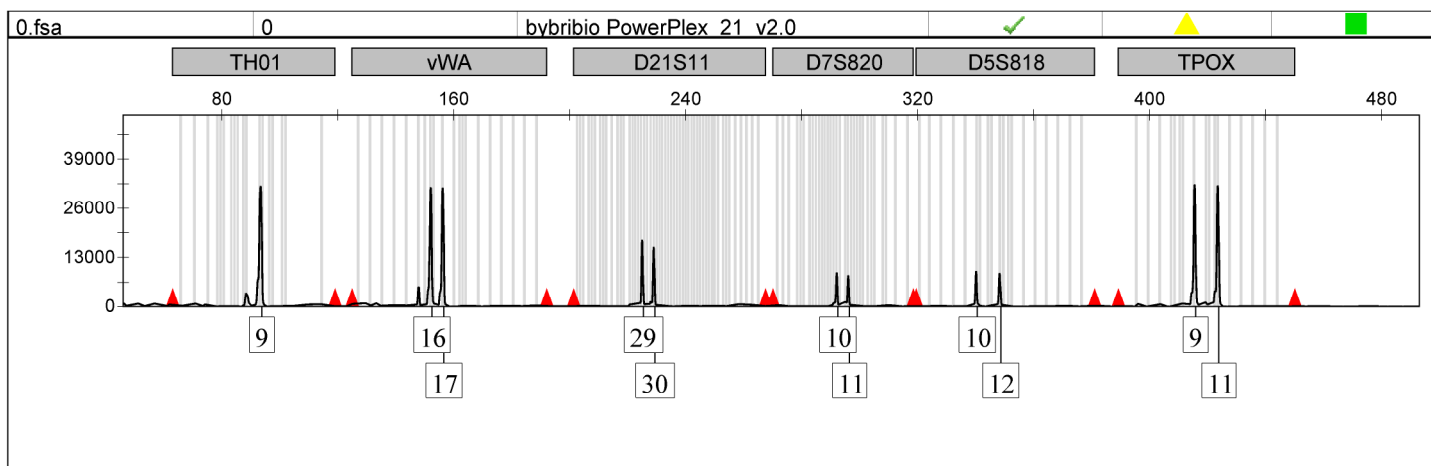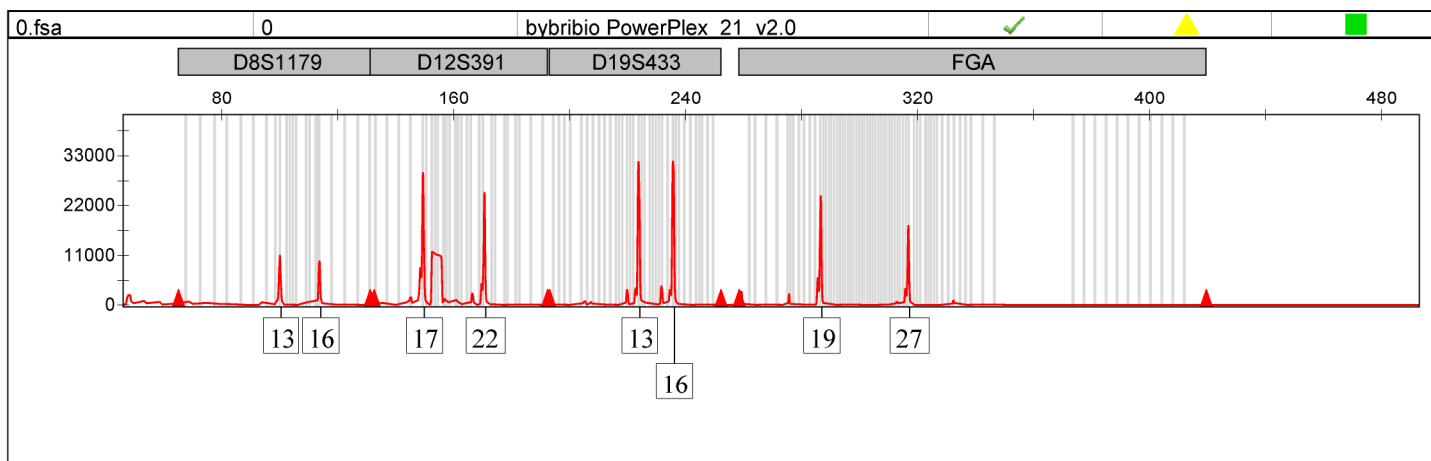

Supplement: Supplementary file 4 — Source Data [file 41467_2022_33759_MOESM4_ESM.zip › Source data/Supplementary fig 32-40/Supplementary Fig 32.pdf]

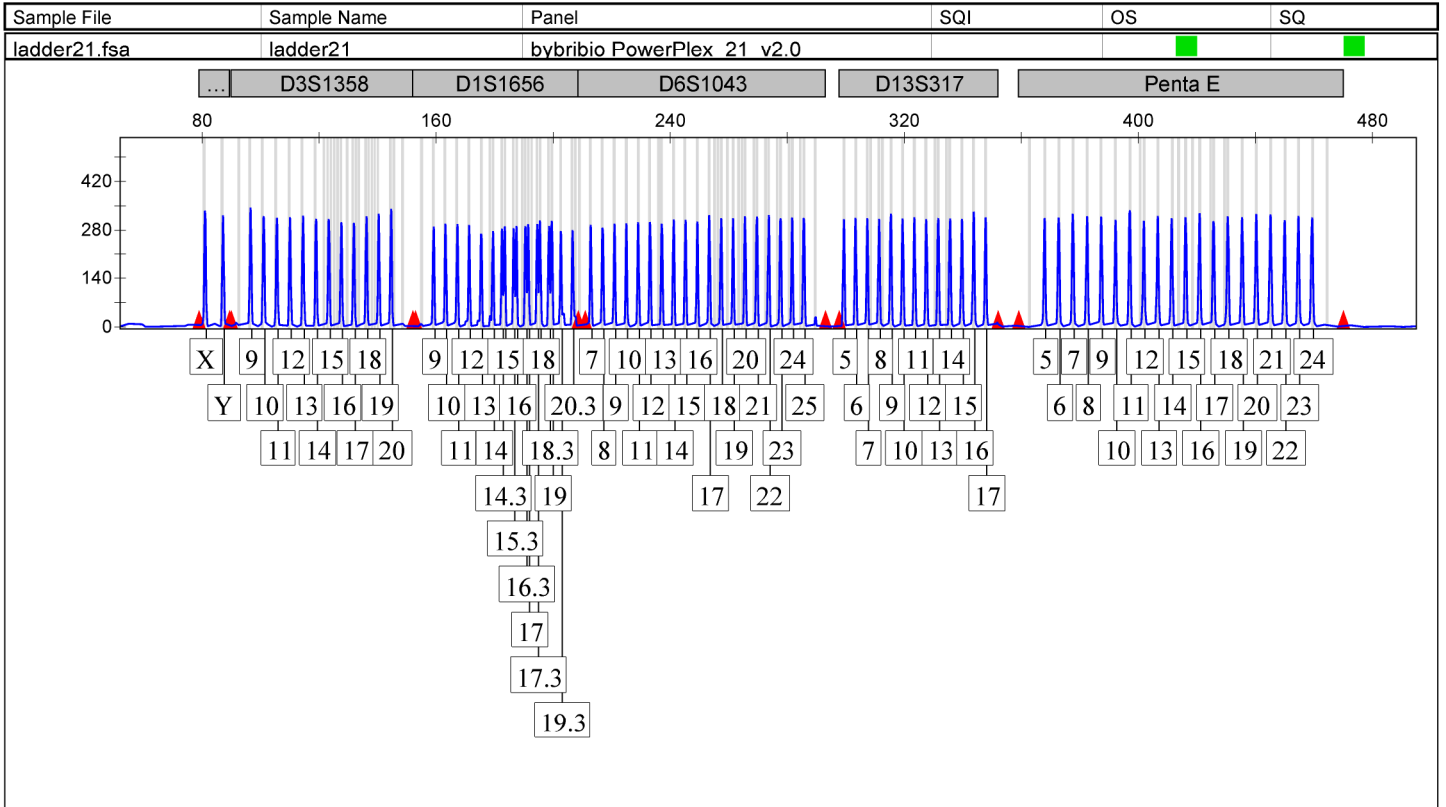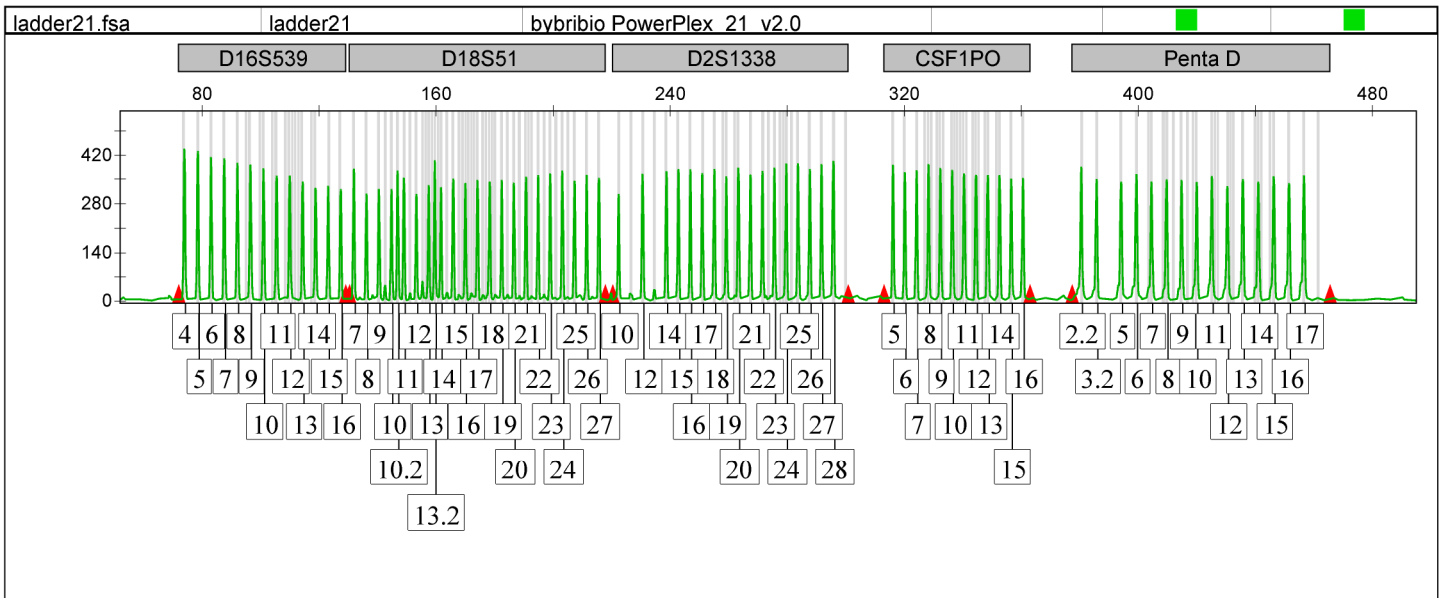

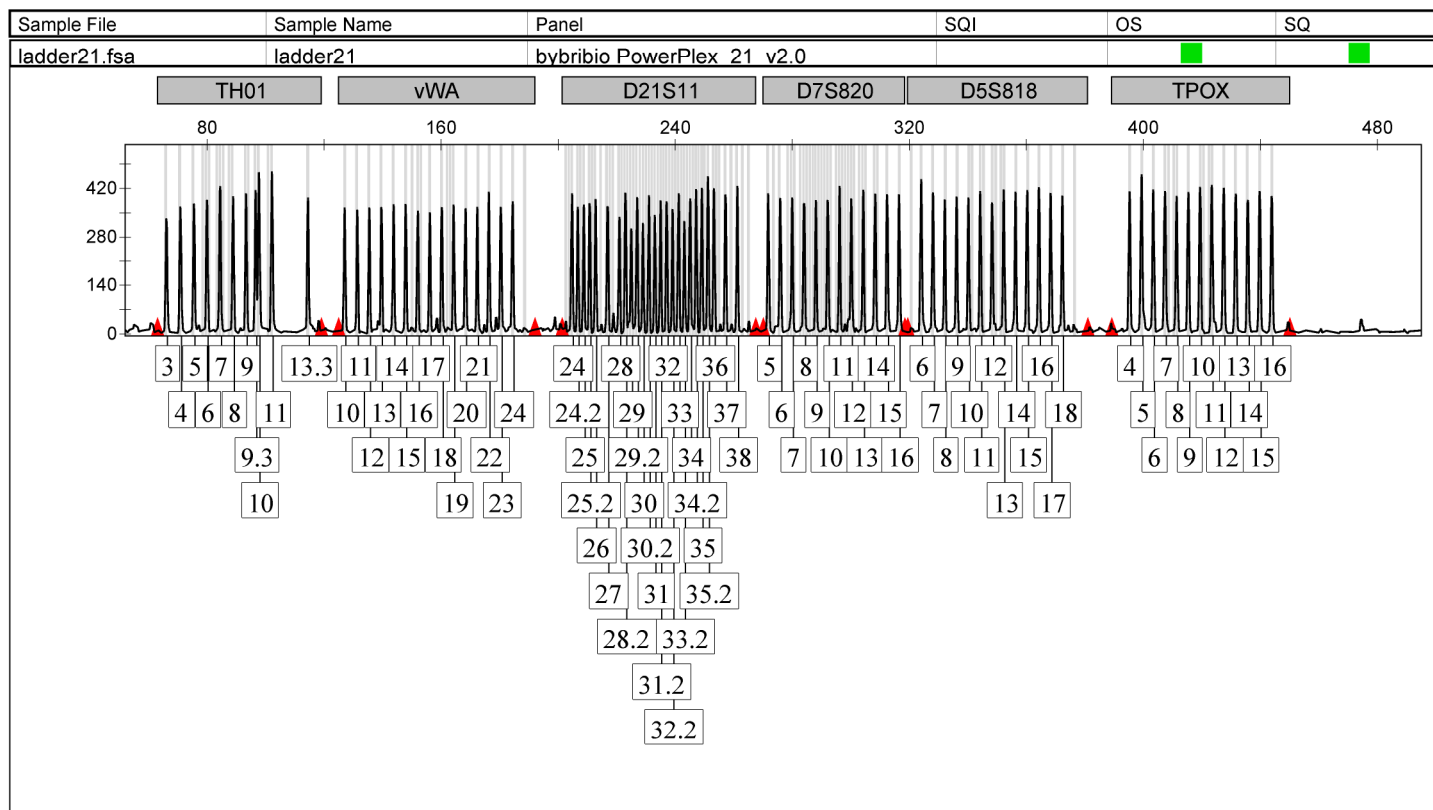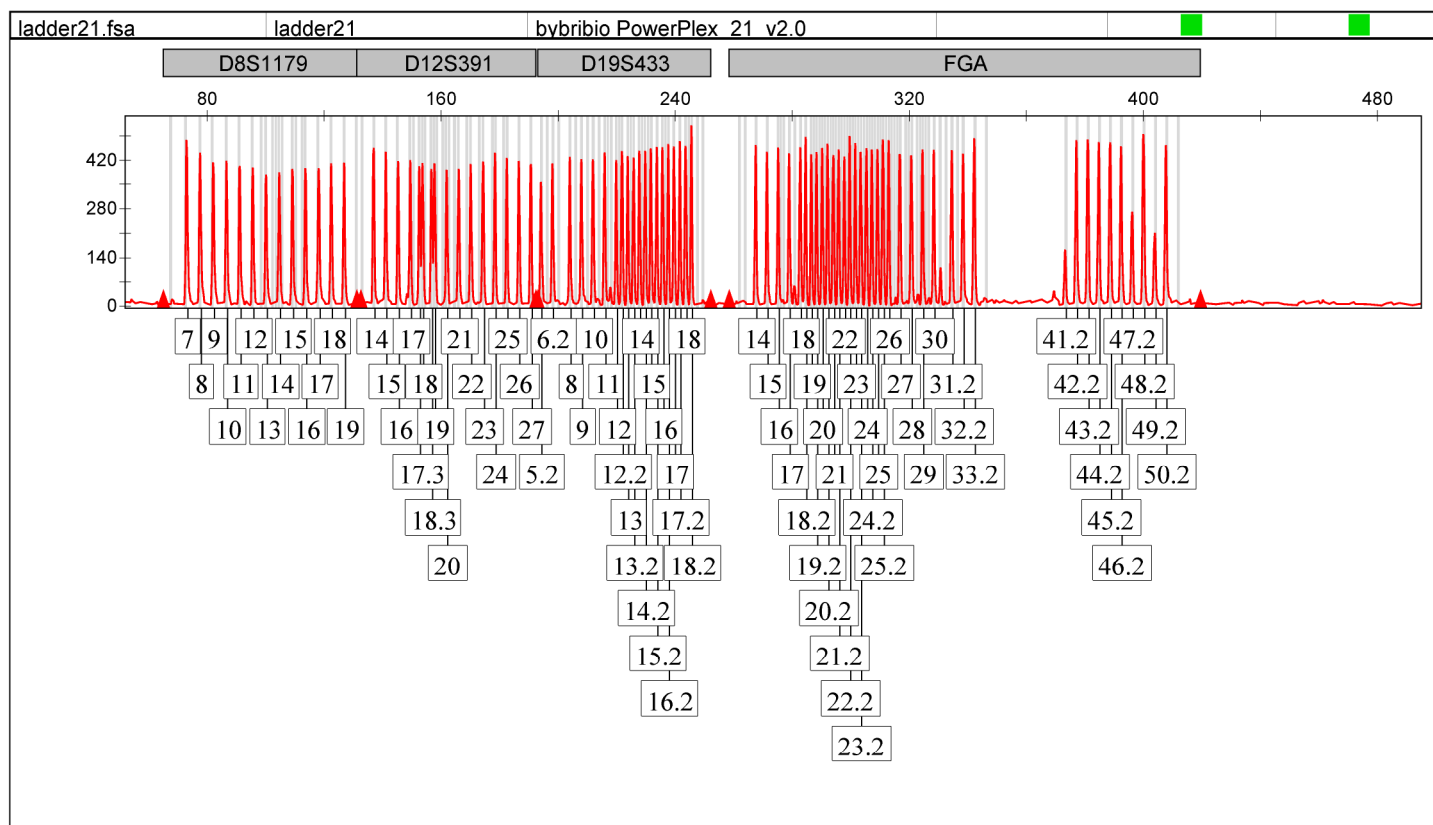

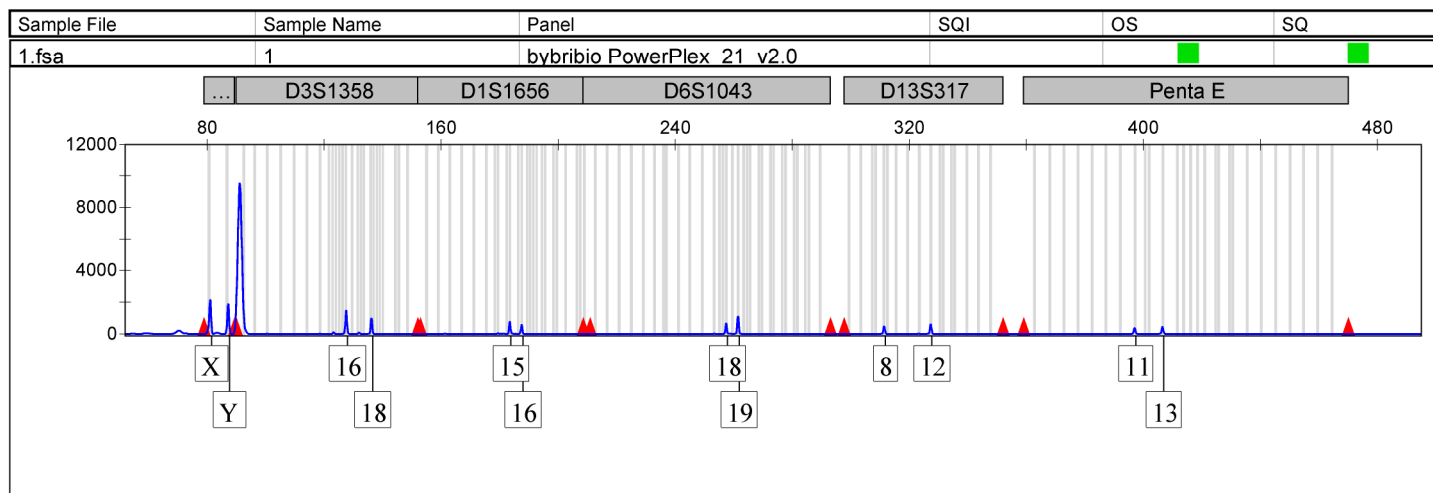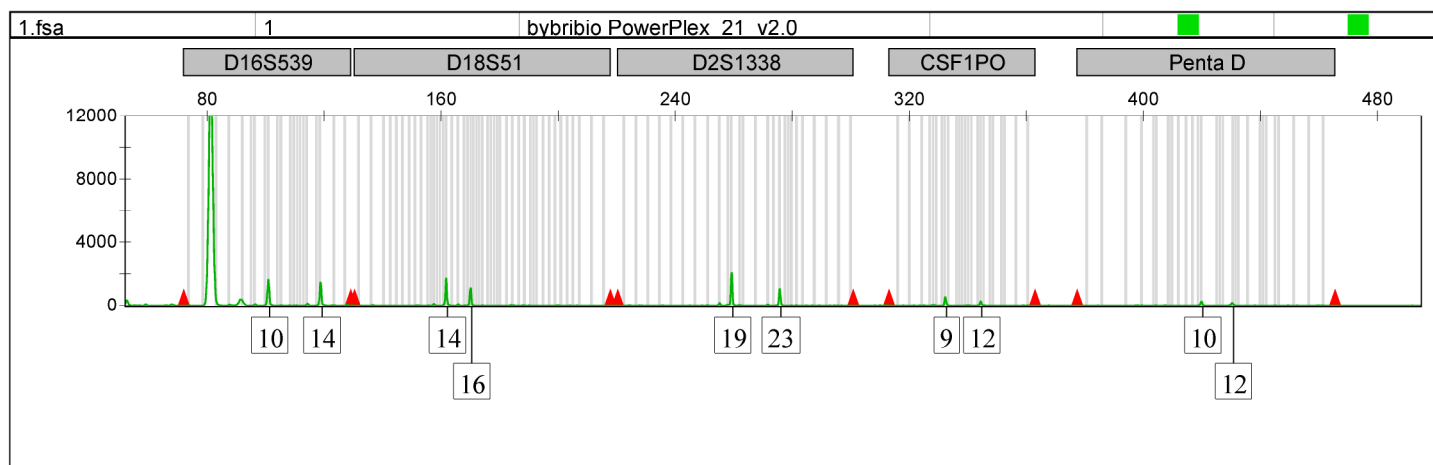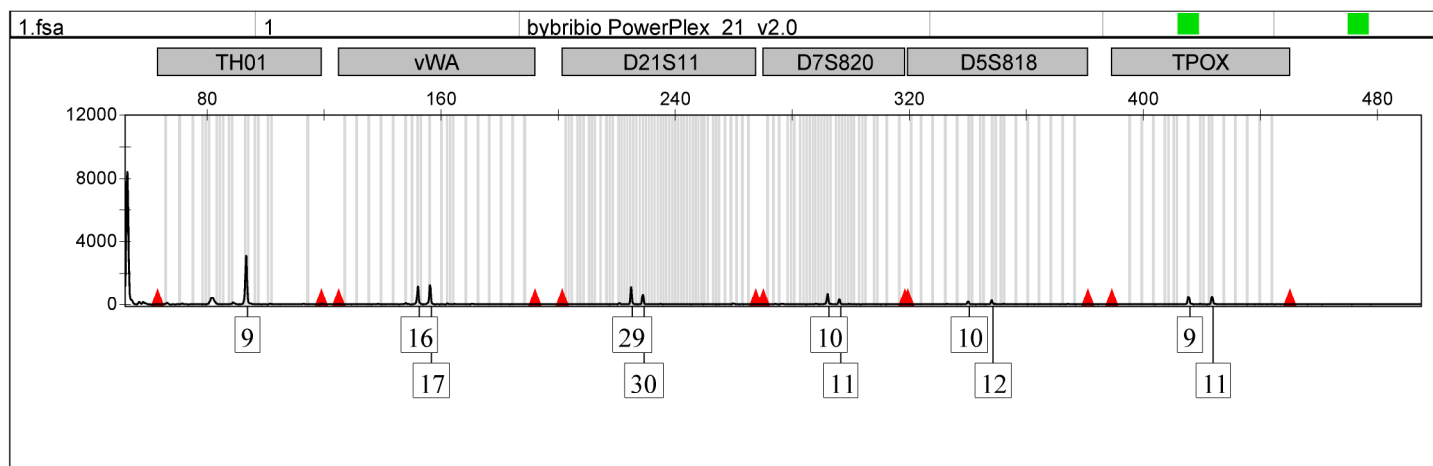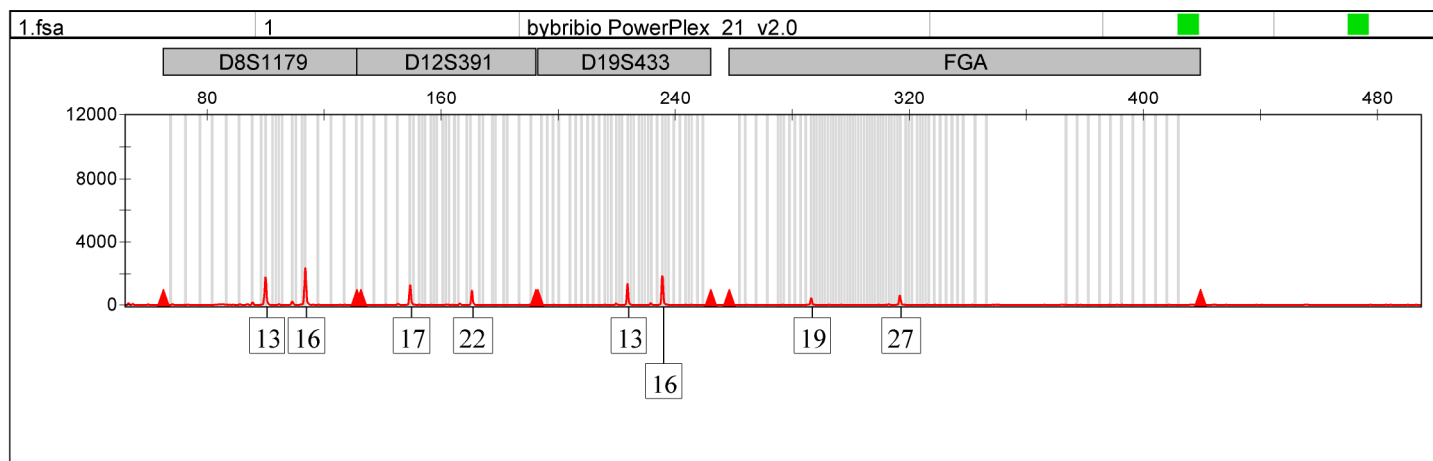

Supplement: Supplementary file 4 — Source Data [file 41467_2022_33759_MOESM4_ESM.zip › Source data/Supplementary fig 32-40/Supplementary Fig 33a.pdf]

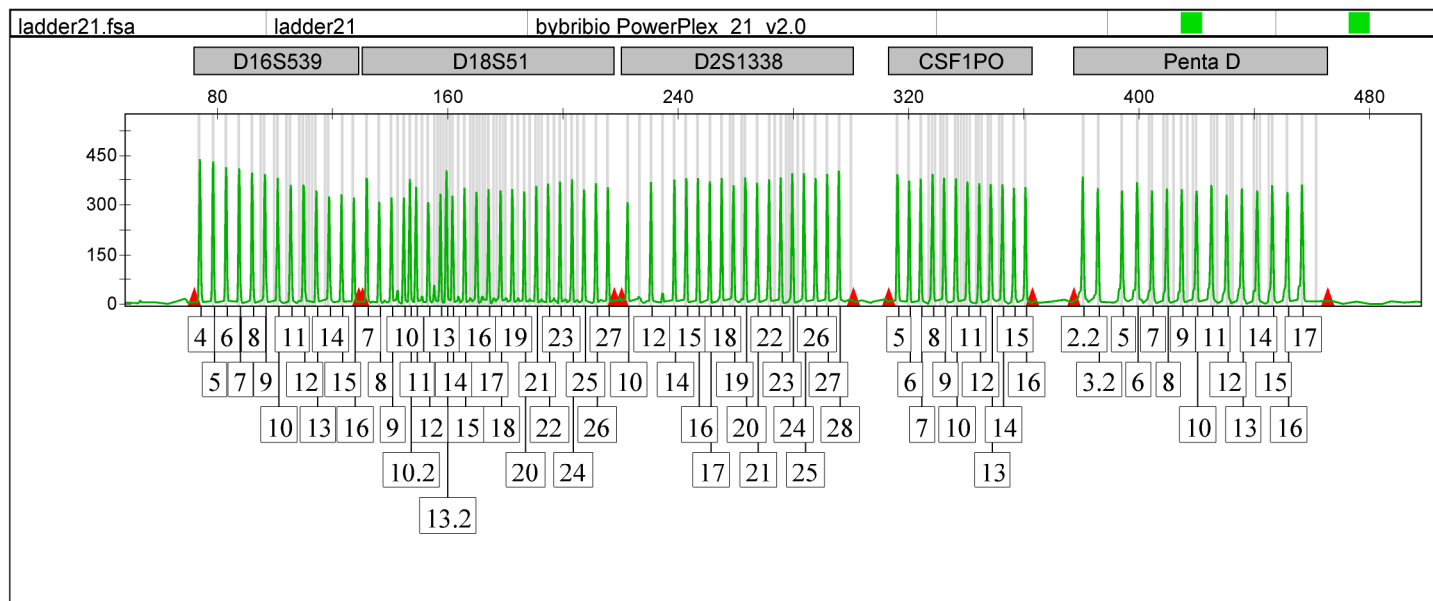

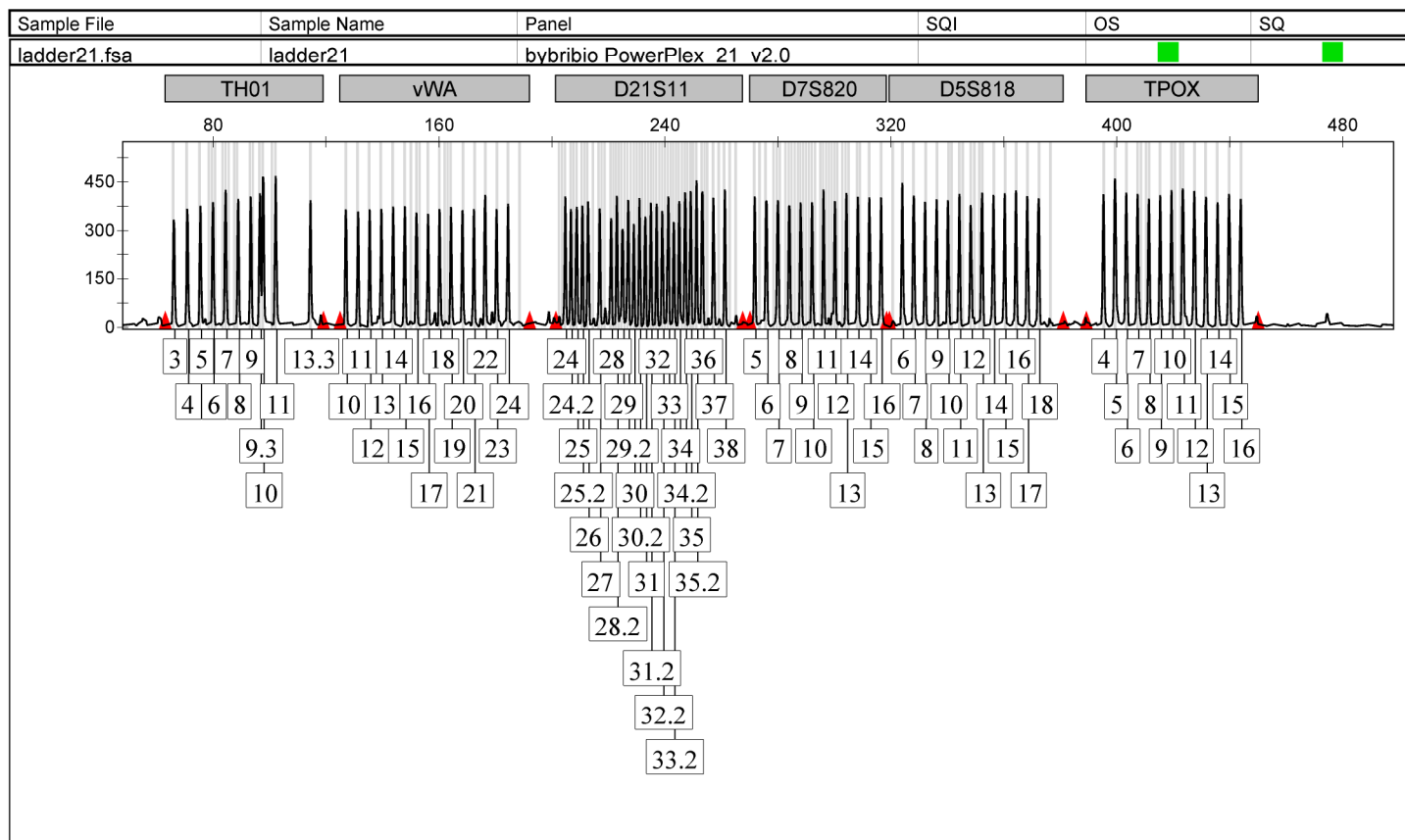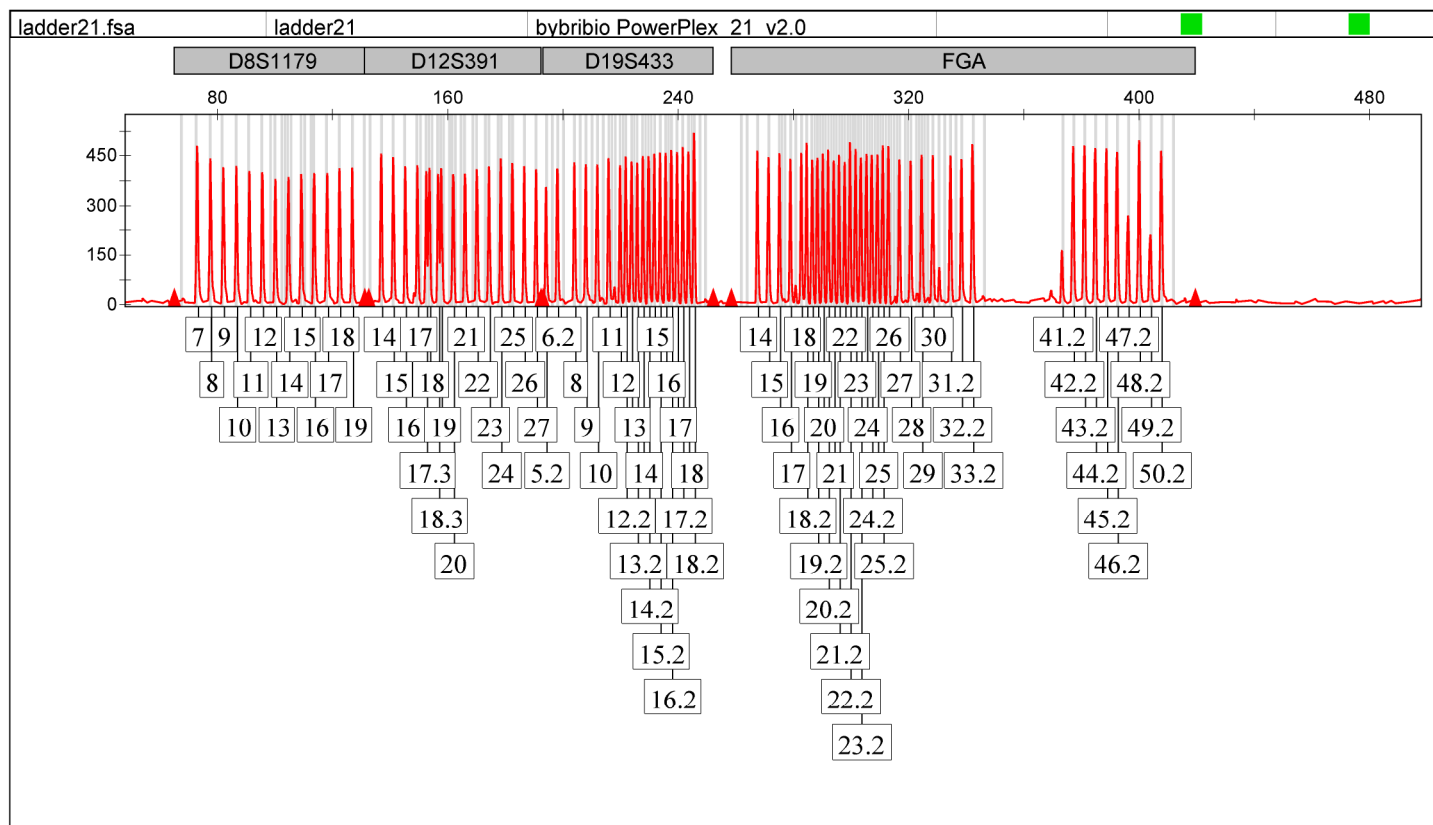

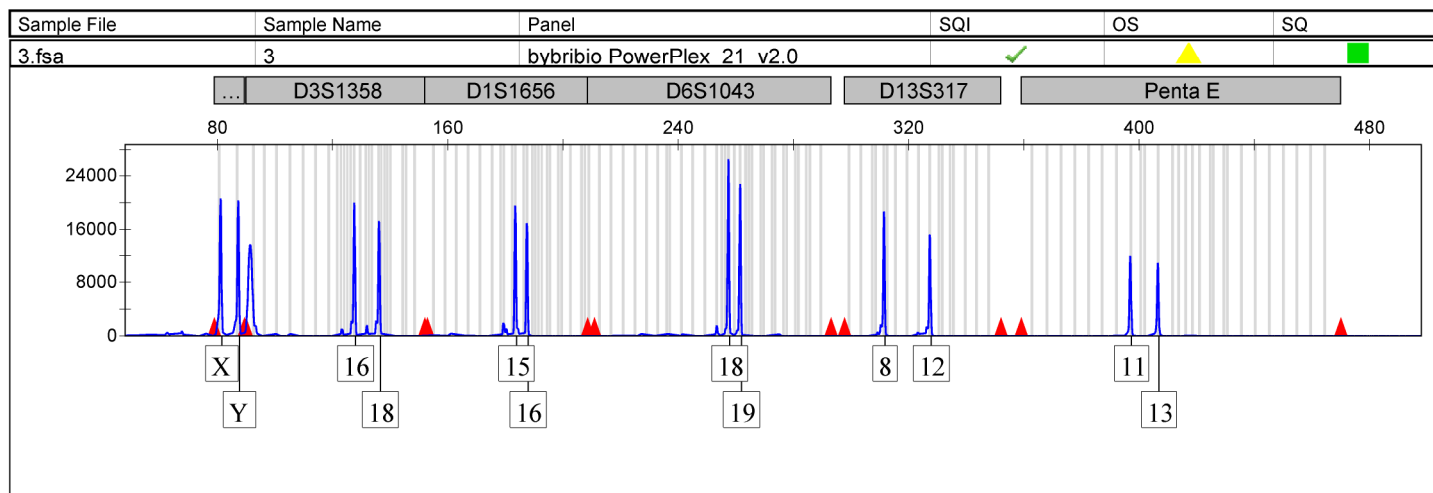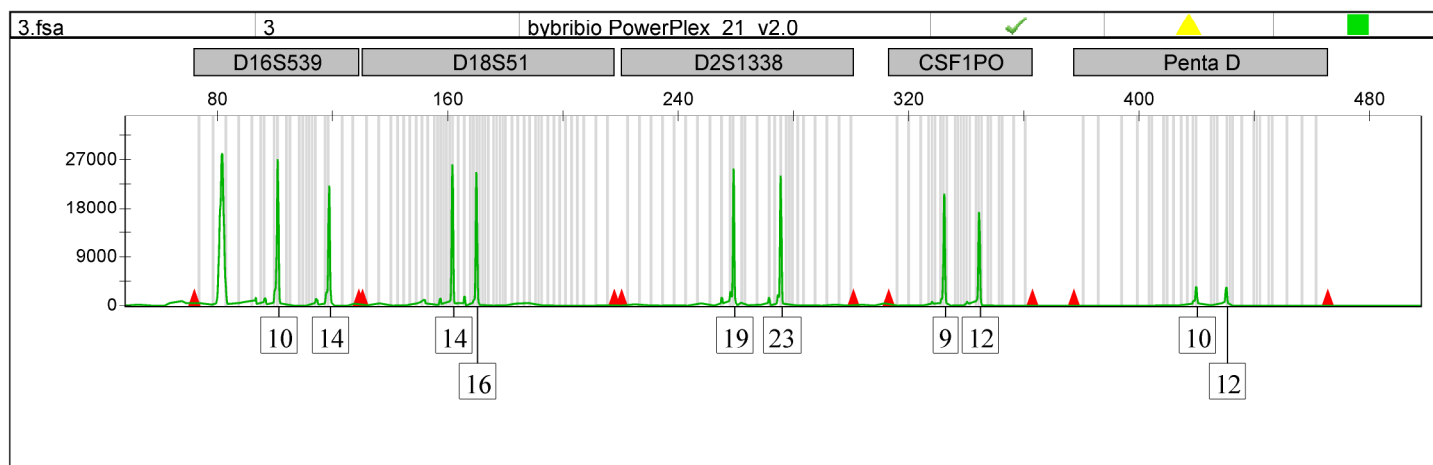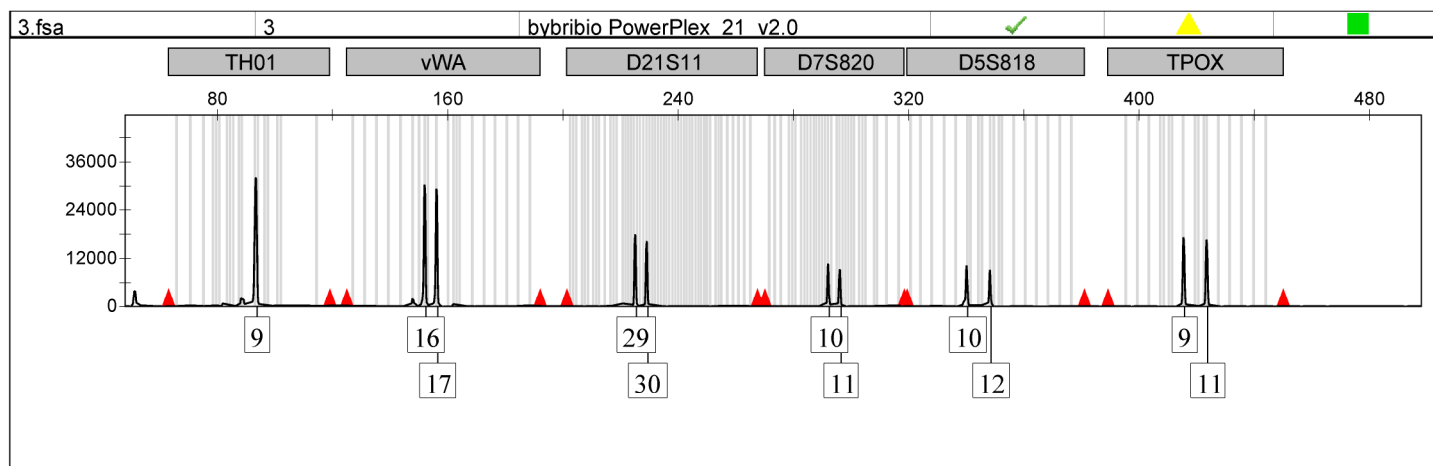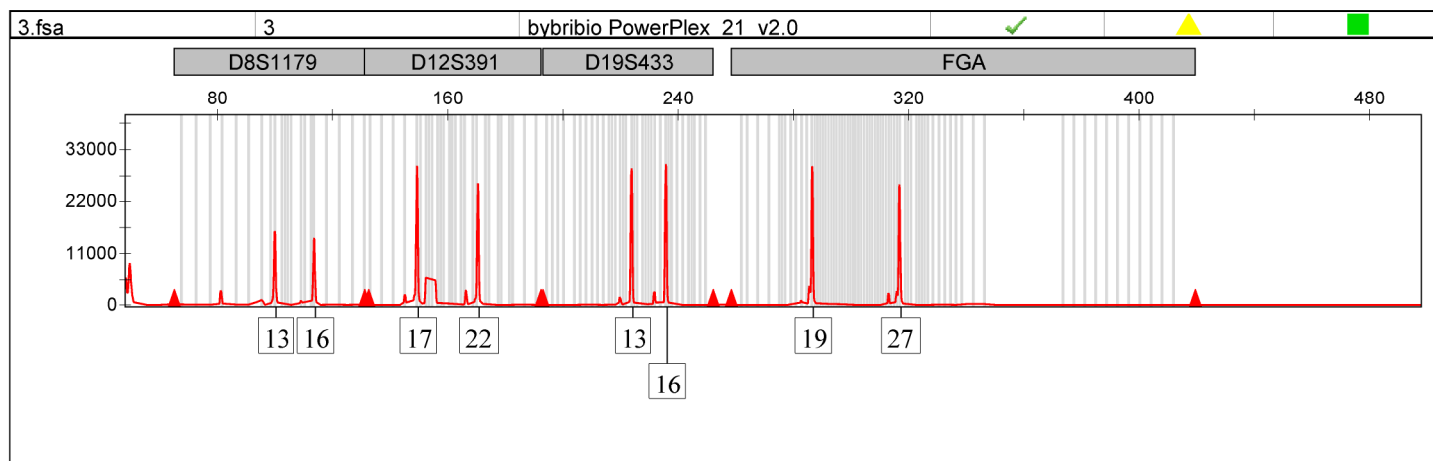

Supplement: Supplementary file 4 — Source Data [file 41467_2022_33759_MOESM4_ESM.zip › Source data/Supplementary fig 32-40/Supplementary Fig 33b.pdf]

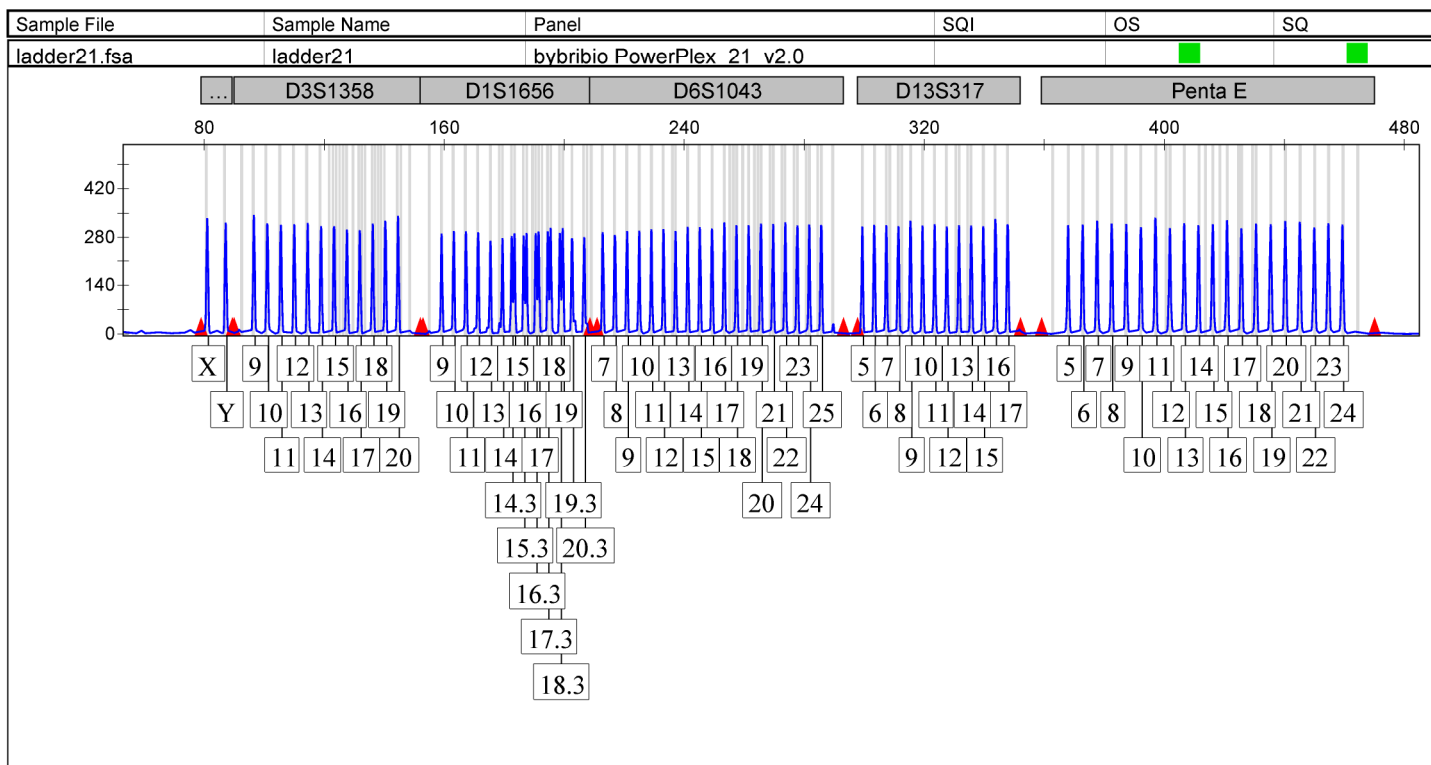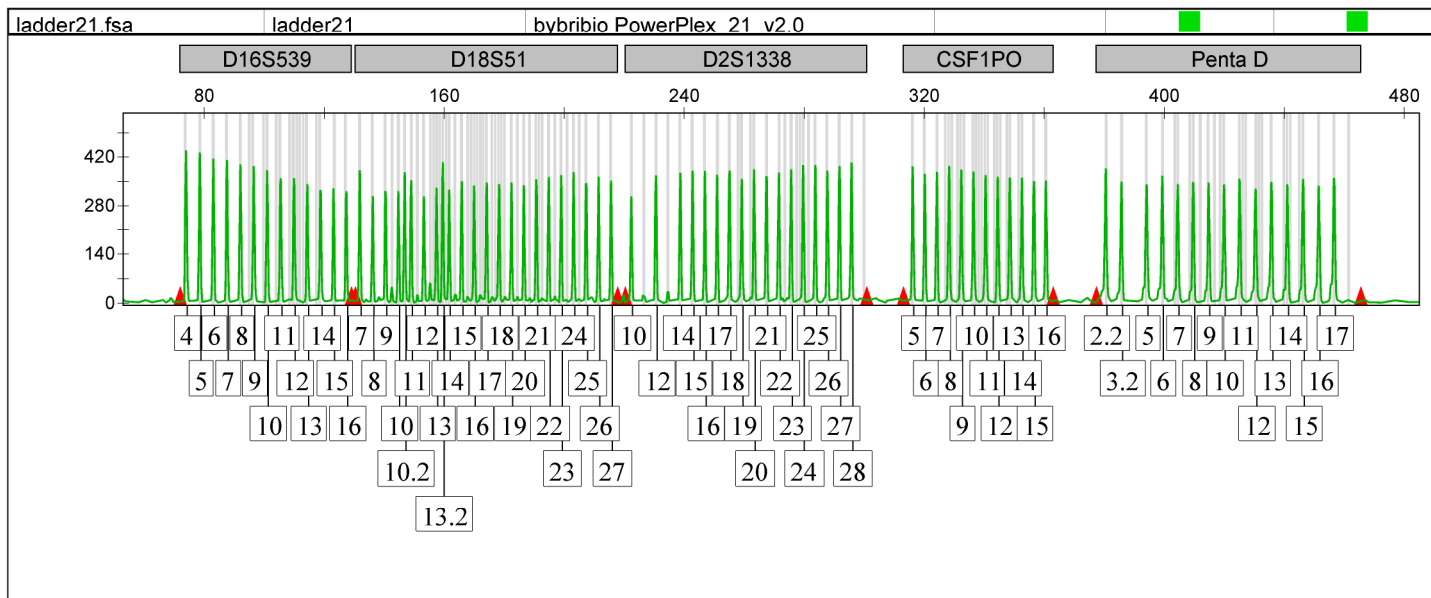

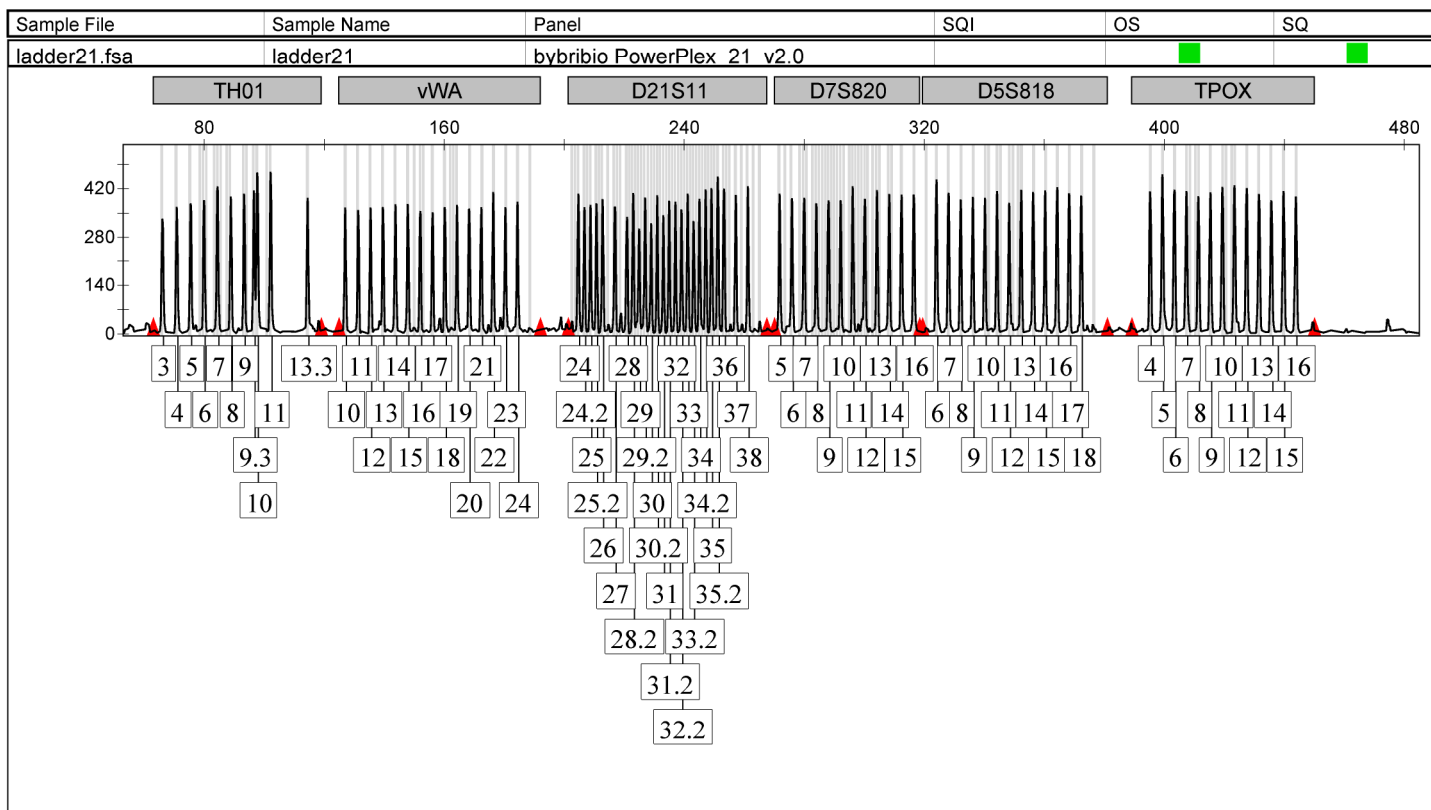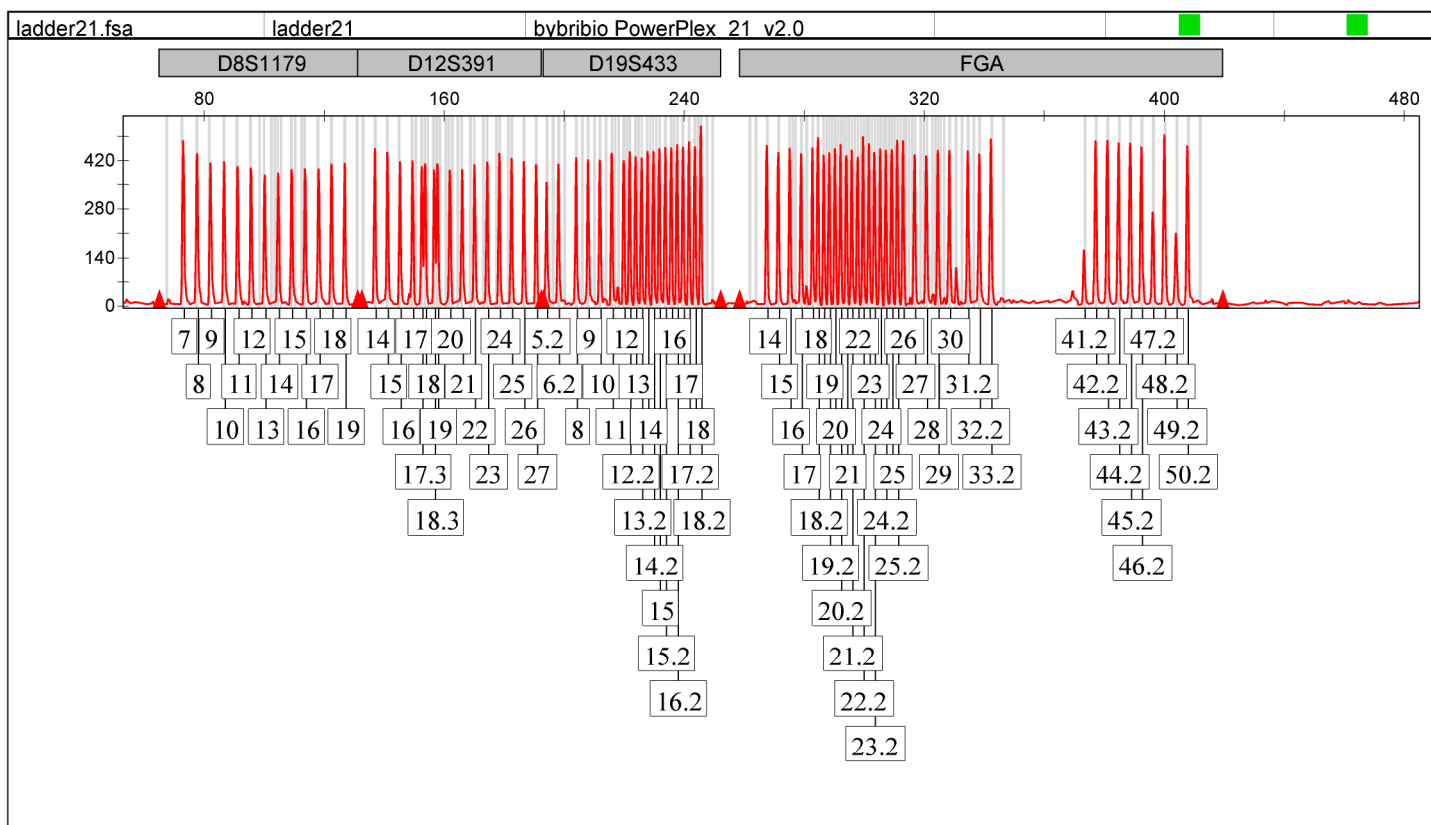

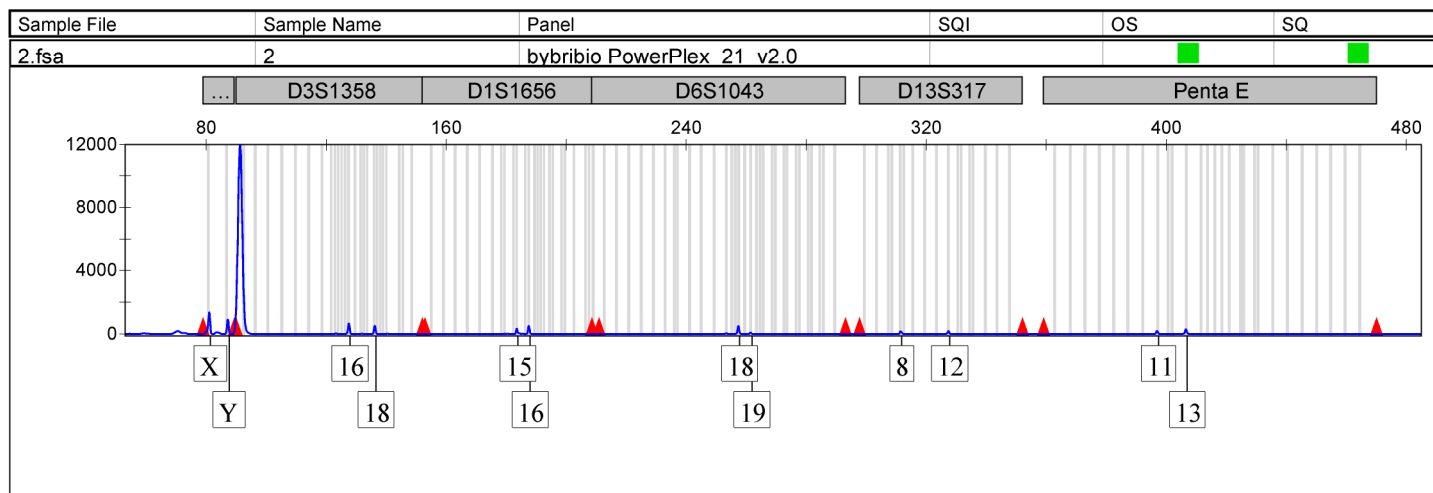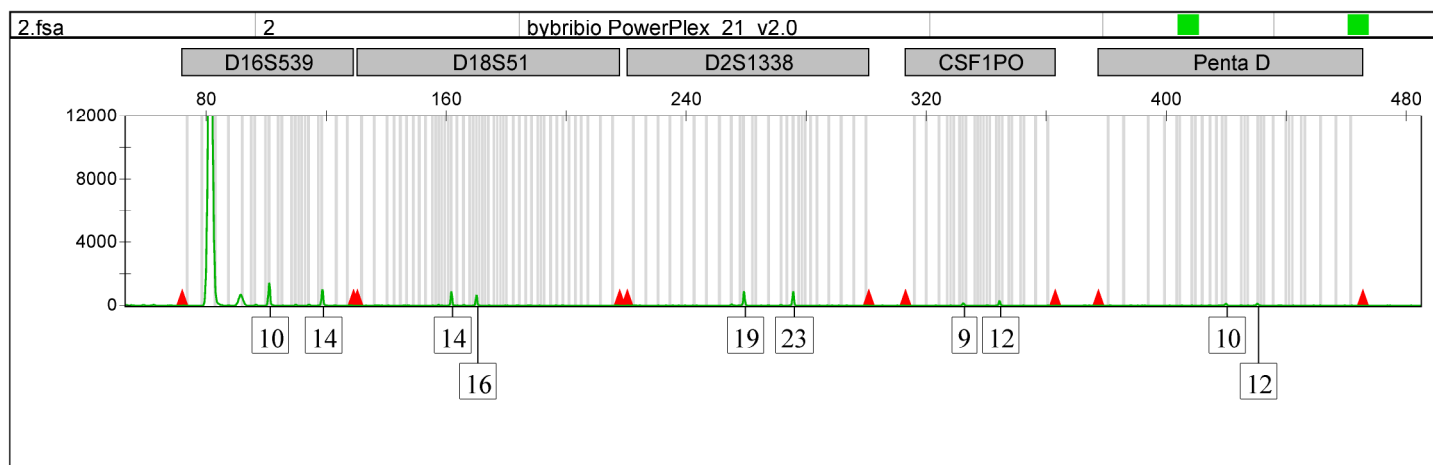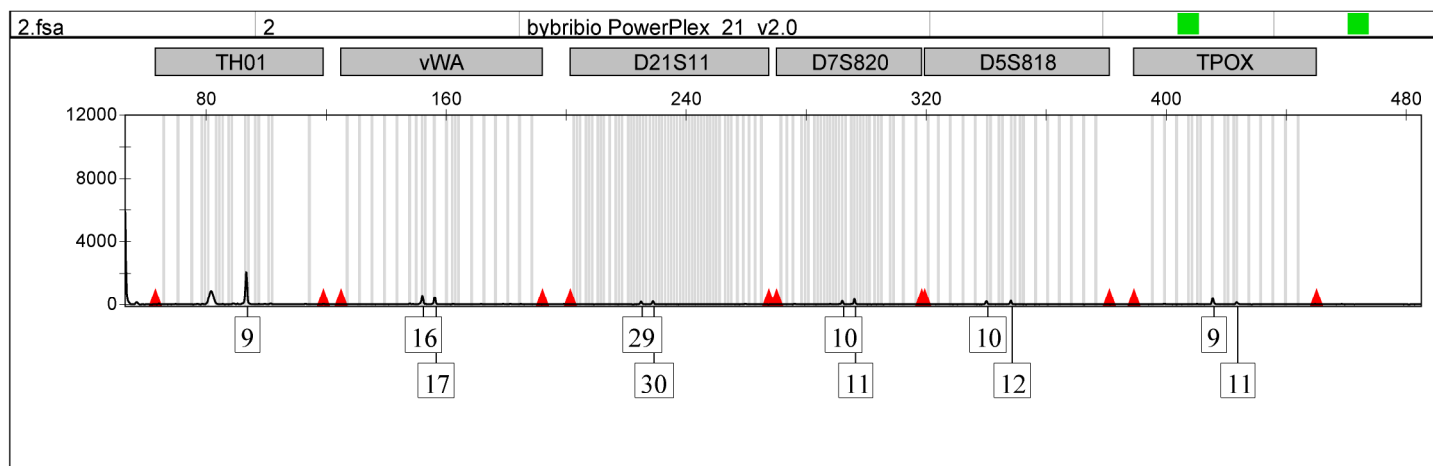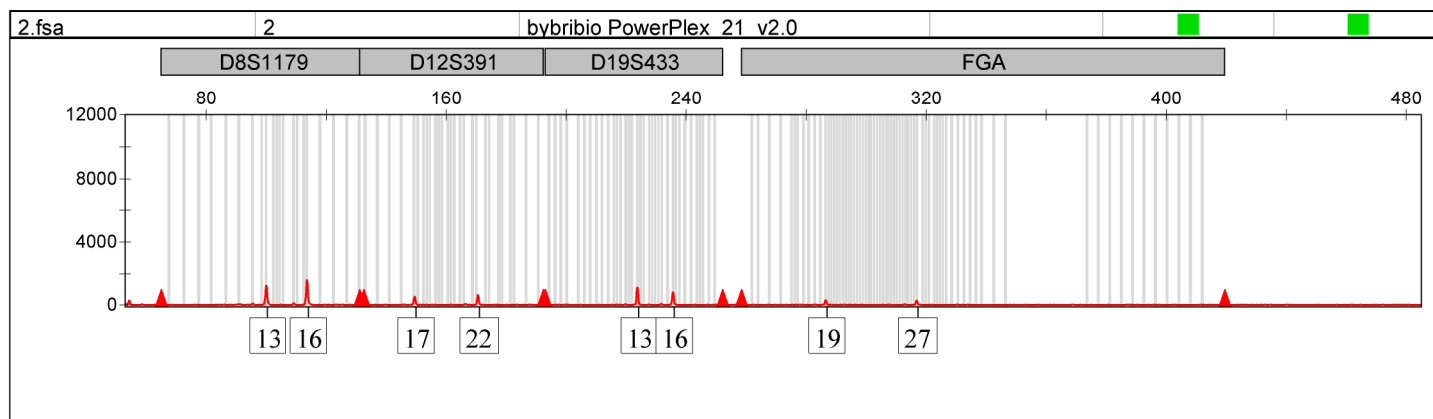

Supplement: Supplementary file 4 — Source Data [file 41467_2022_33759_MOESM4_ESM.zip › Source data/Supplementary fig 32-40/Supplementary Fig 34a.pdf]

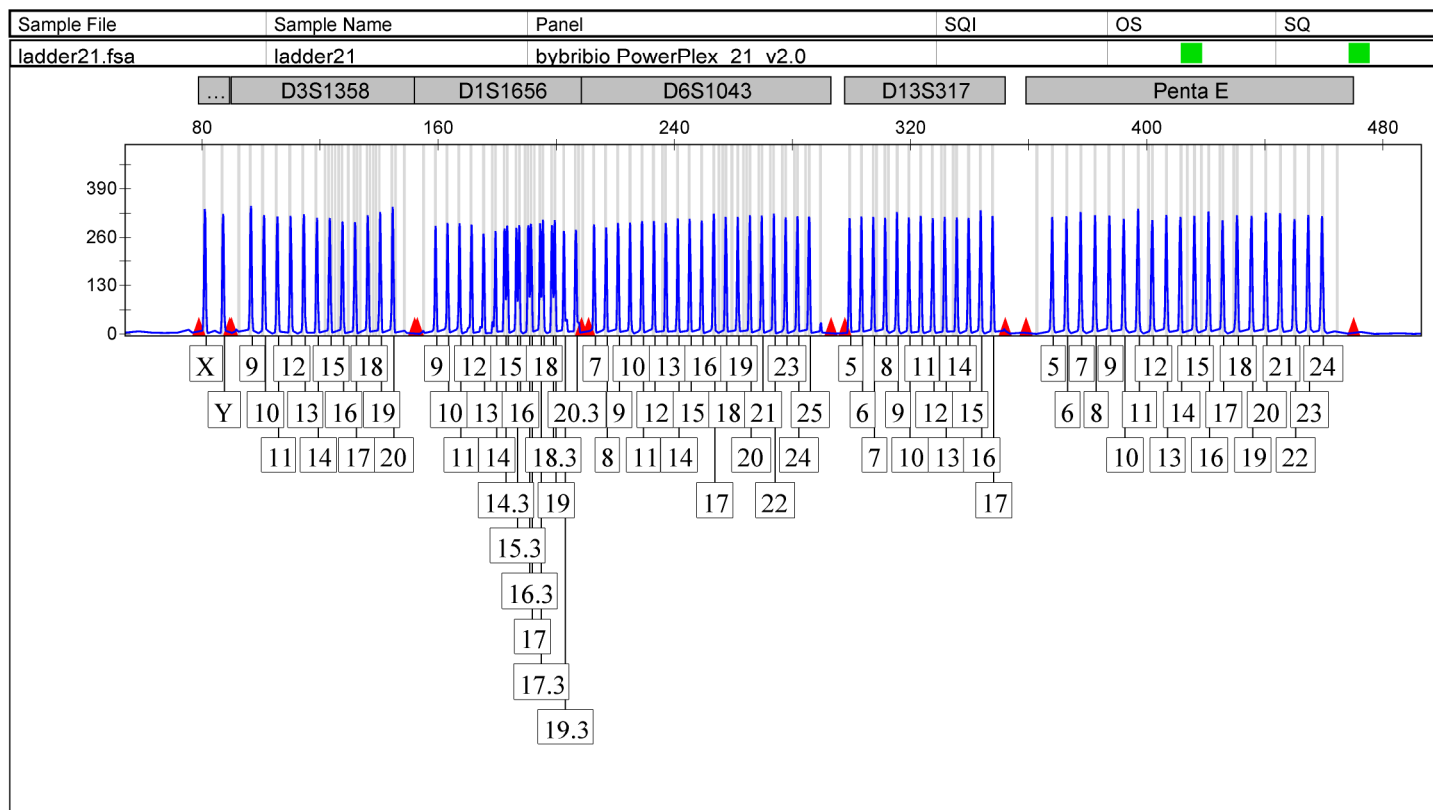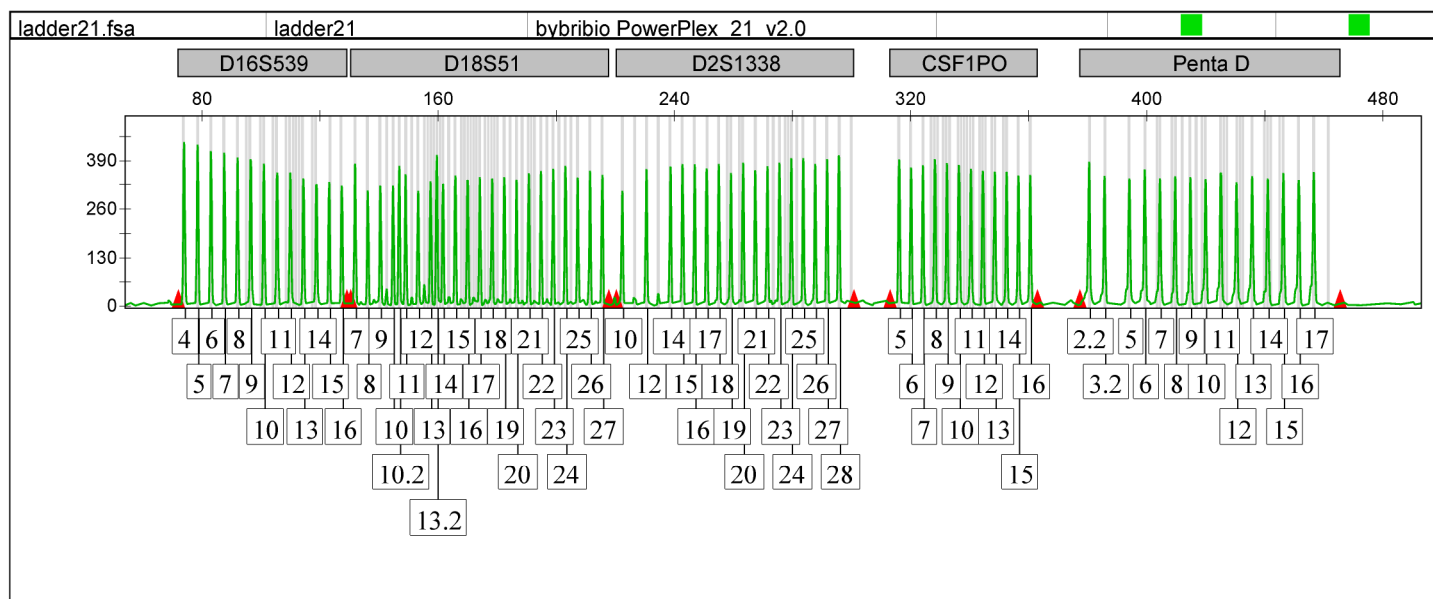

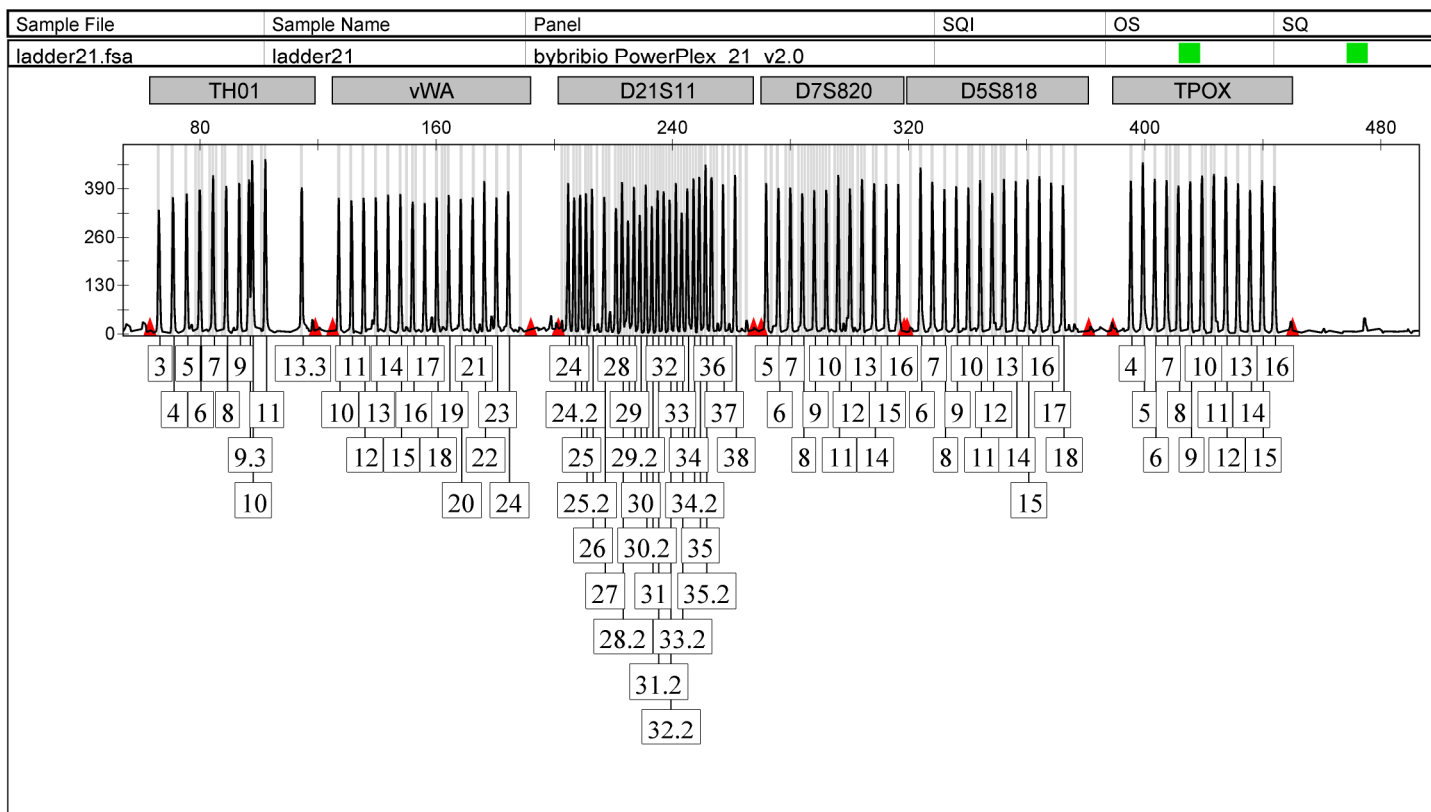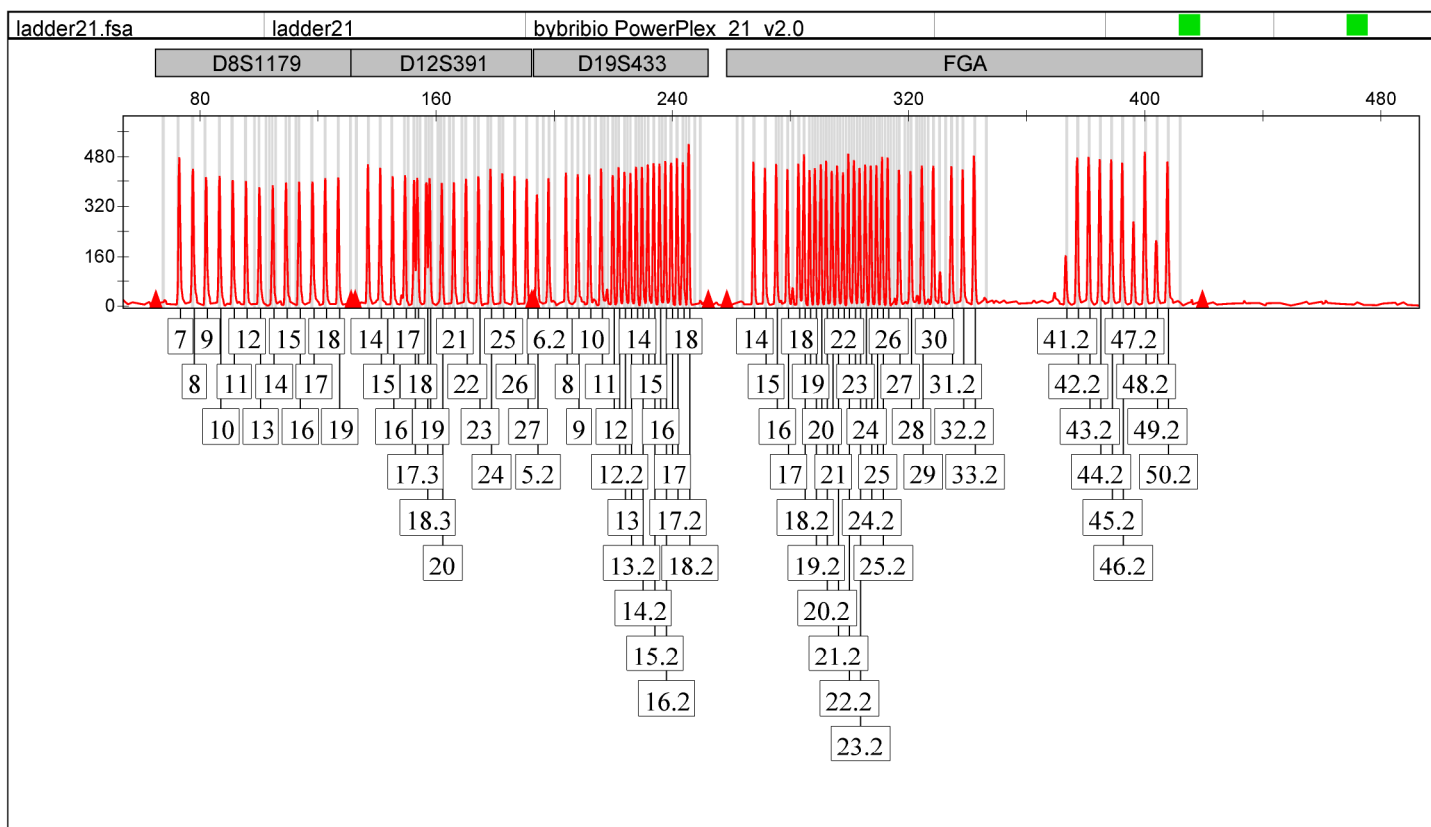

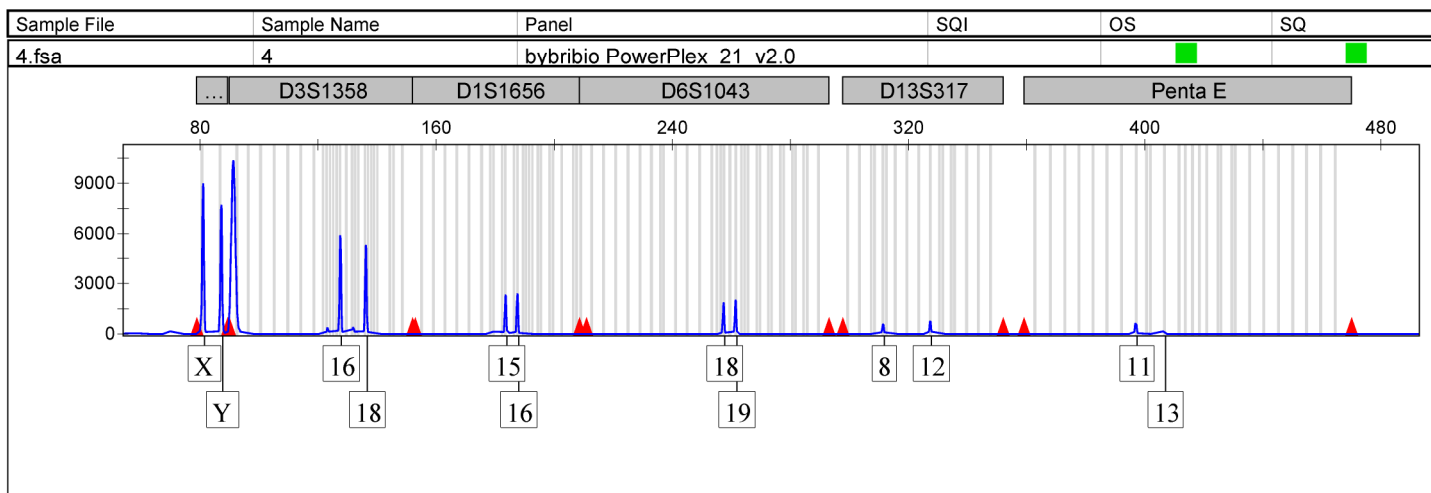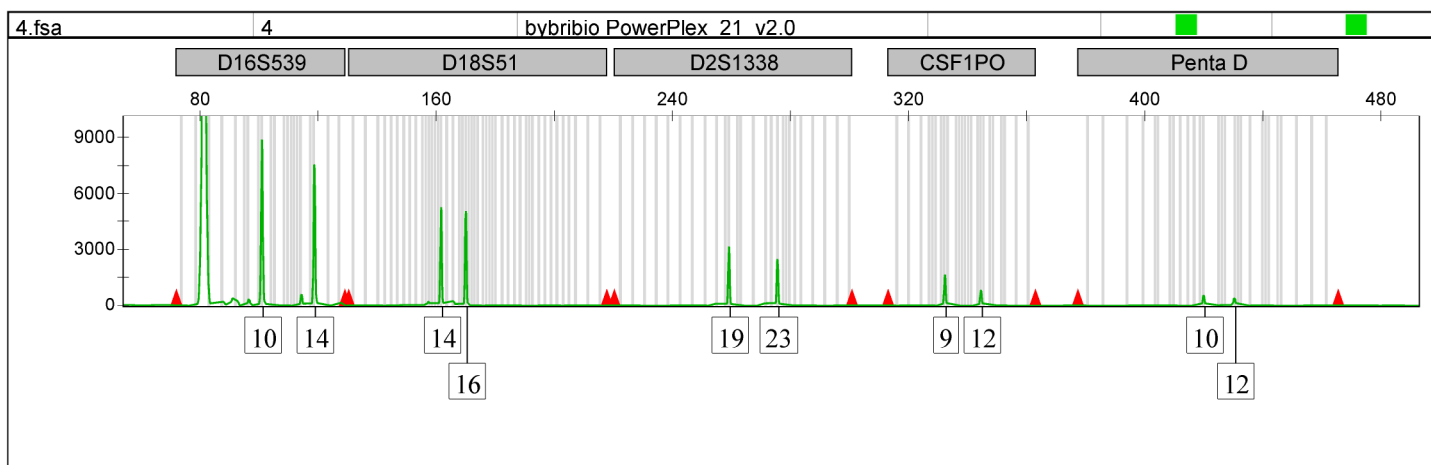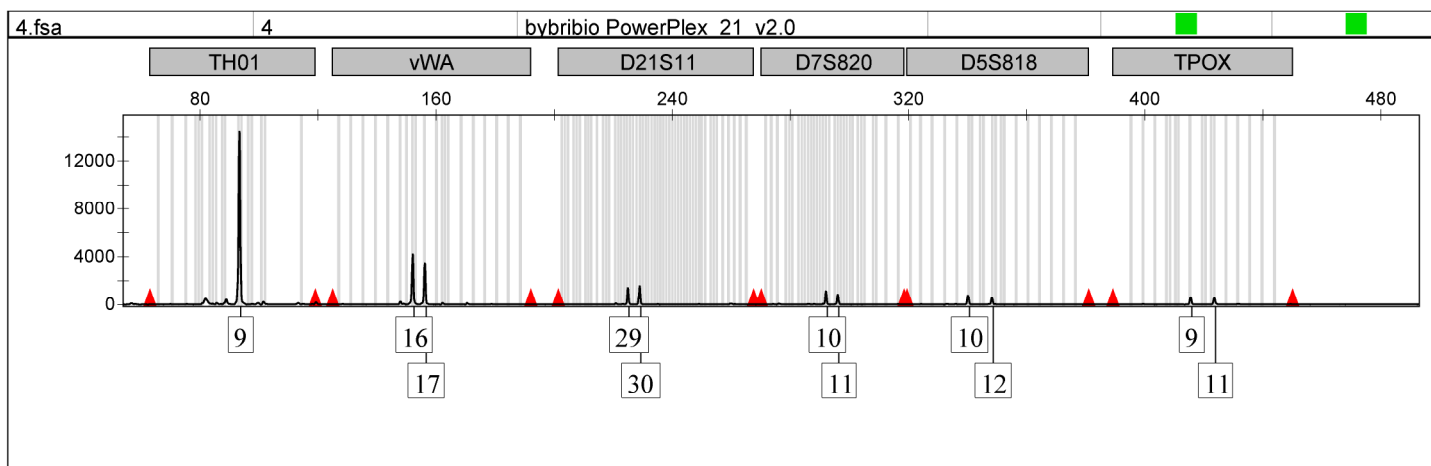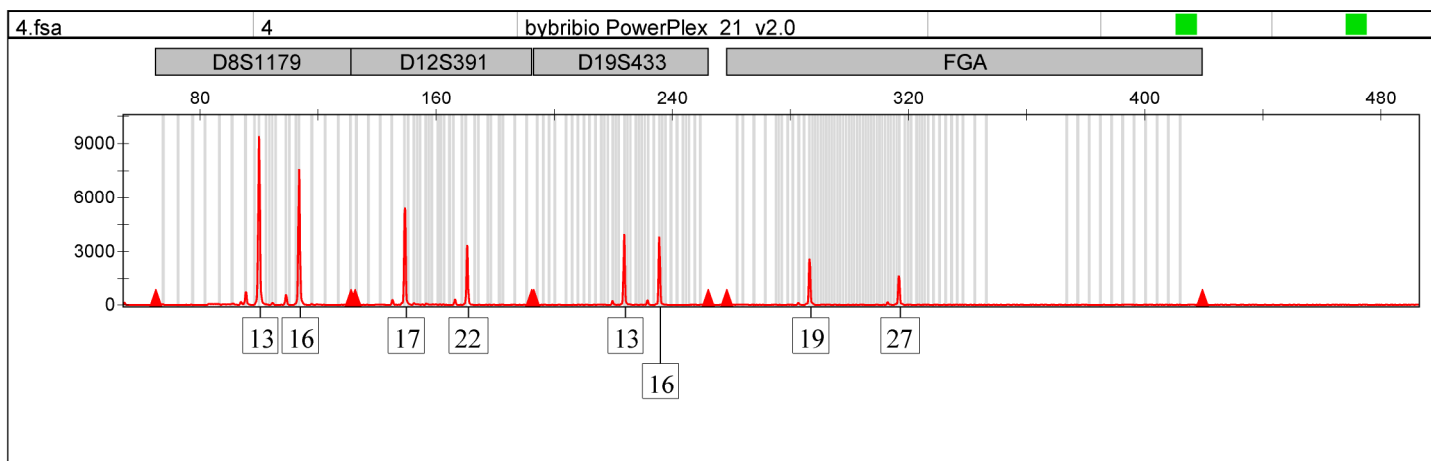

Supplement: Supplementary file 4 — Source Data [file 41467_2022_33759_MOESM4_ESM.zip › Source data/Supplementary fig 32-40/Supplementary Fig 34b.pdf]

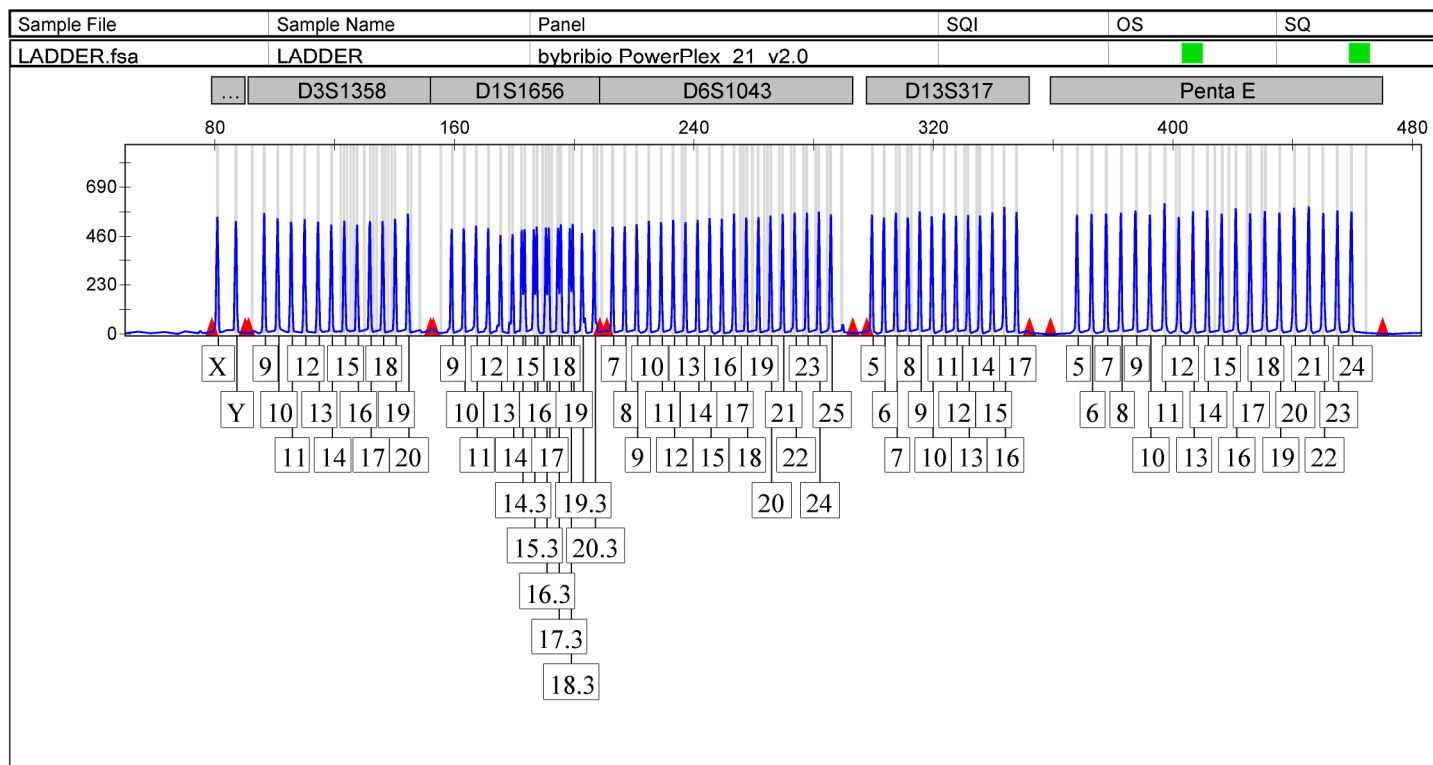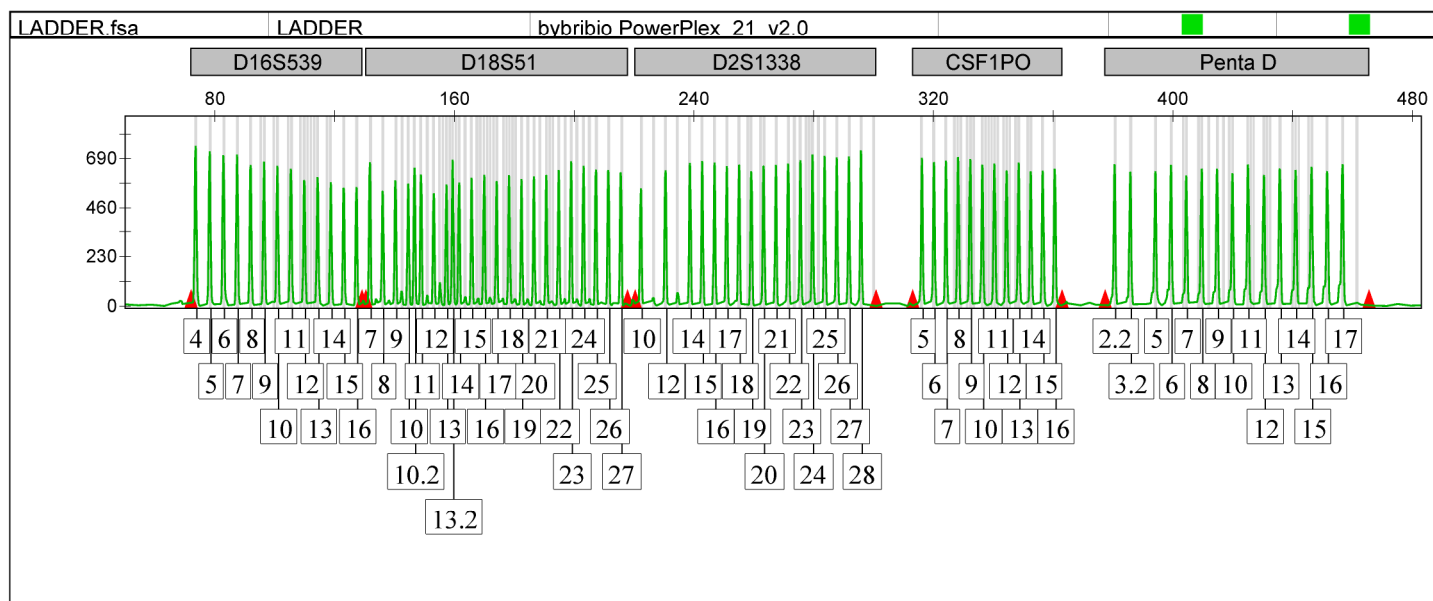

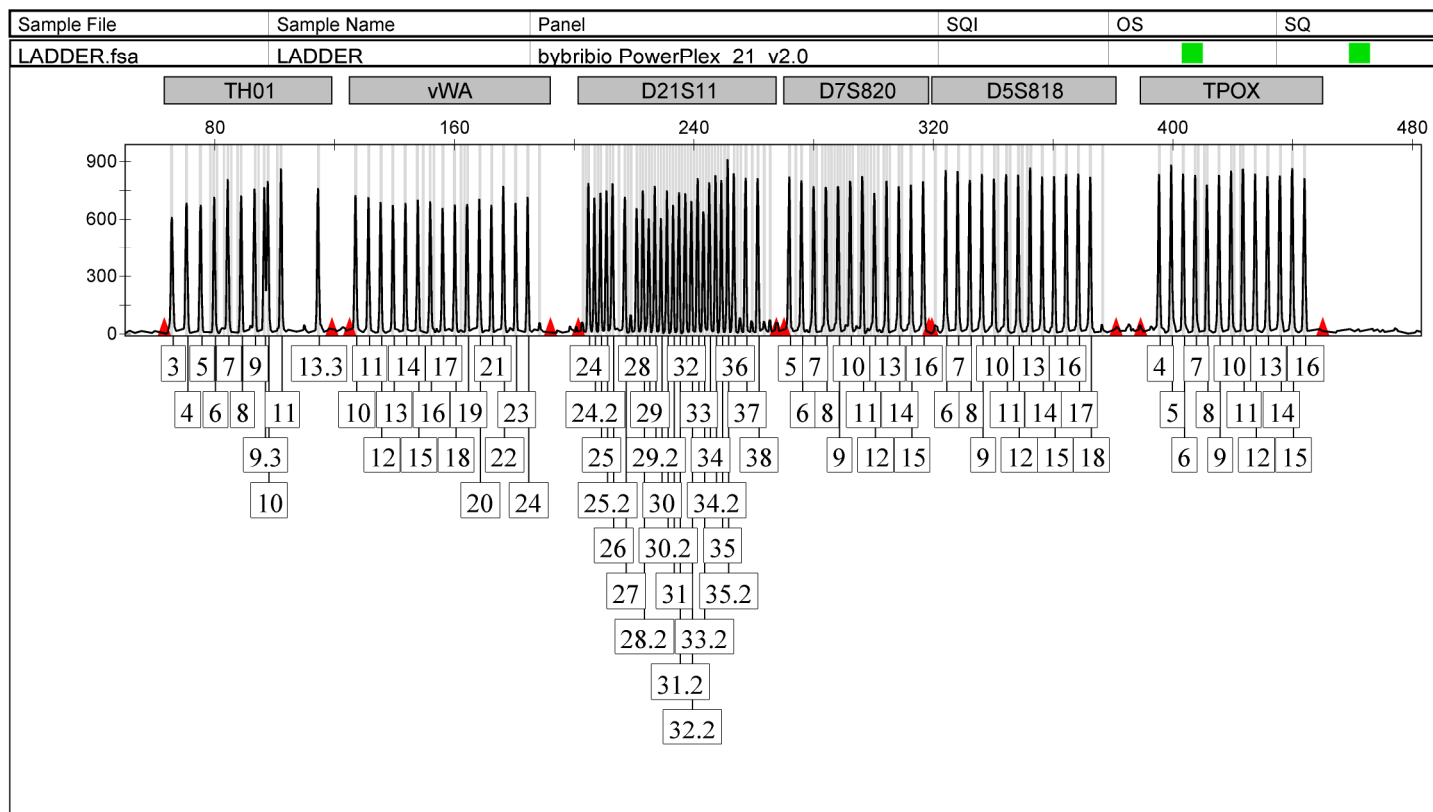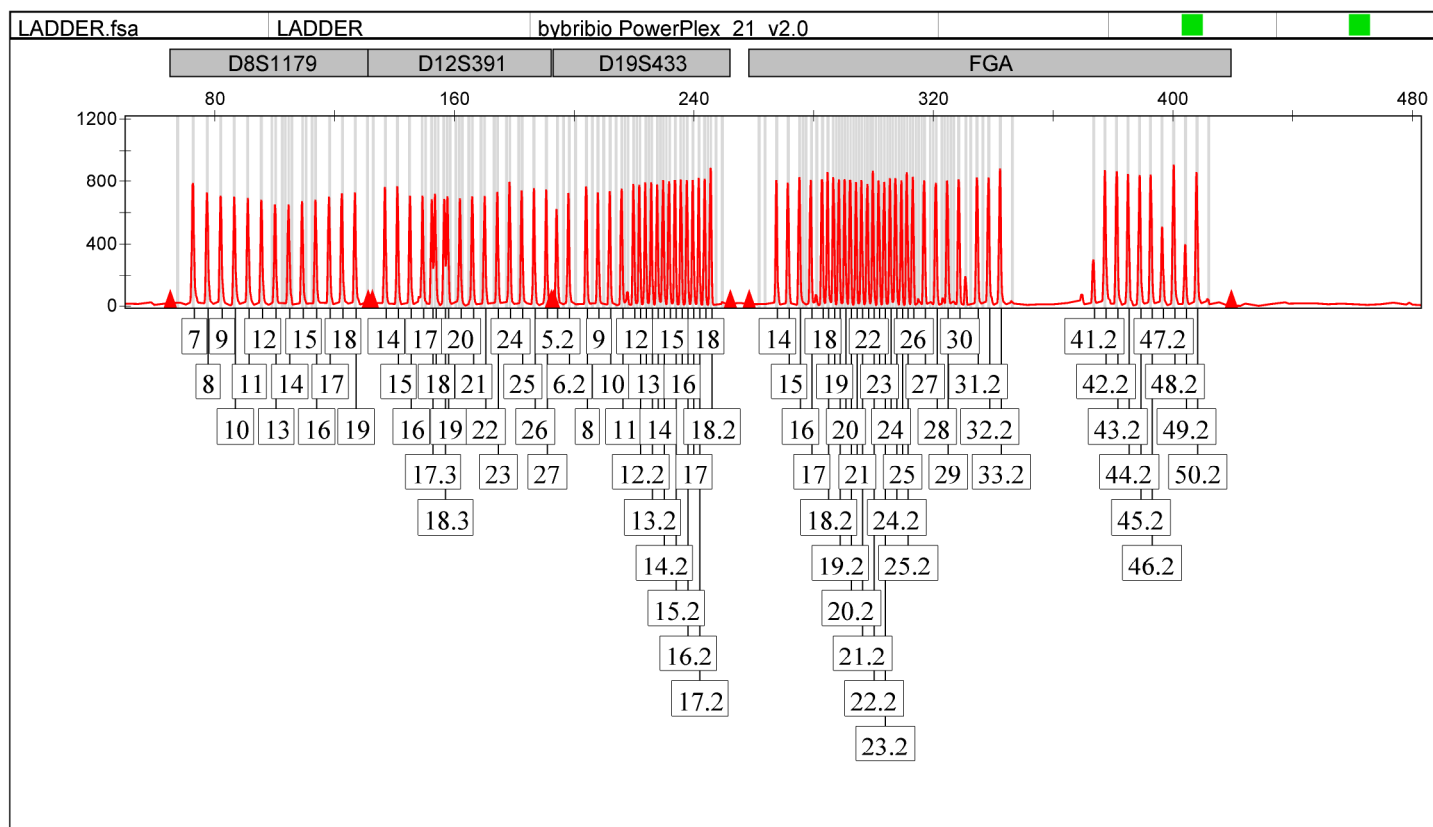

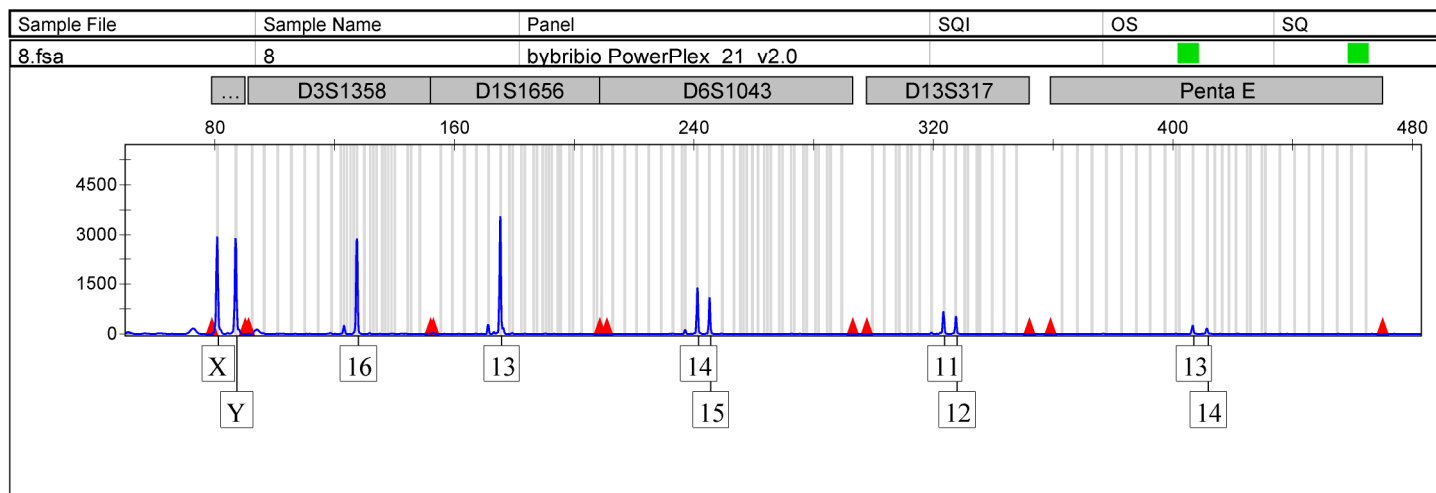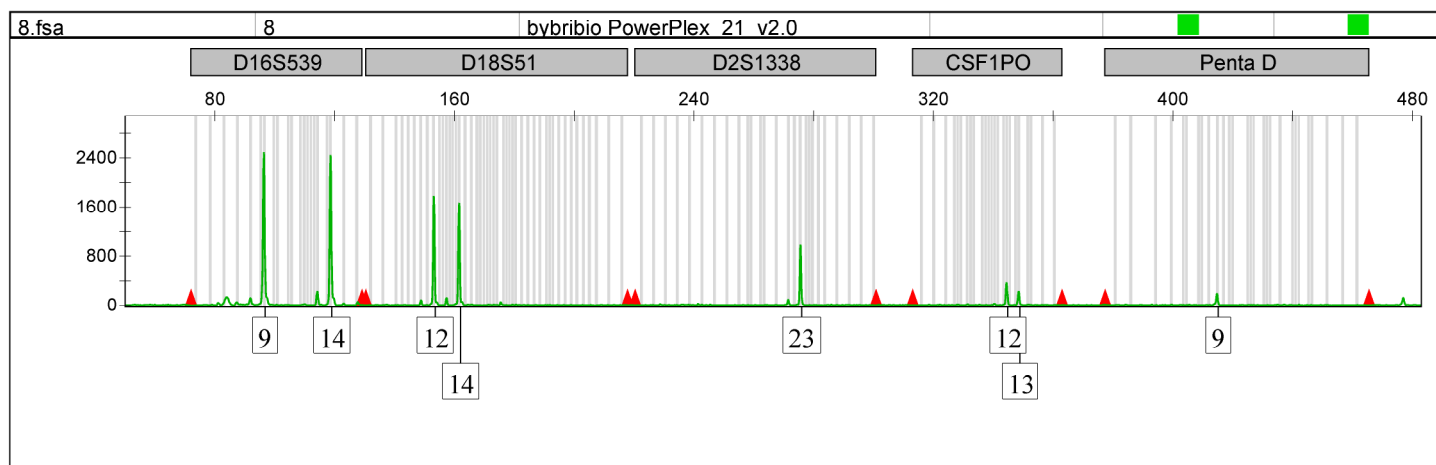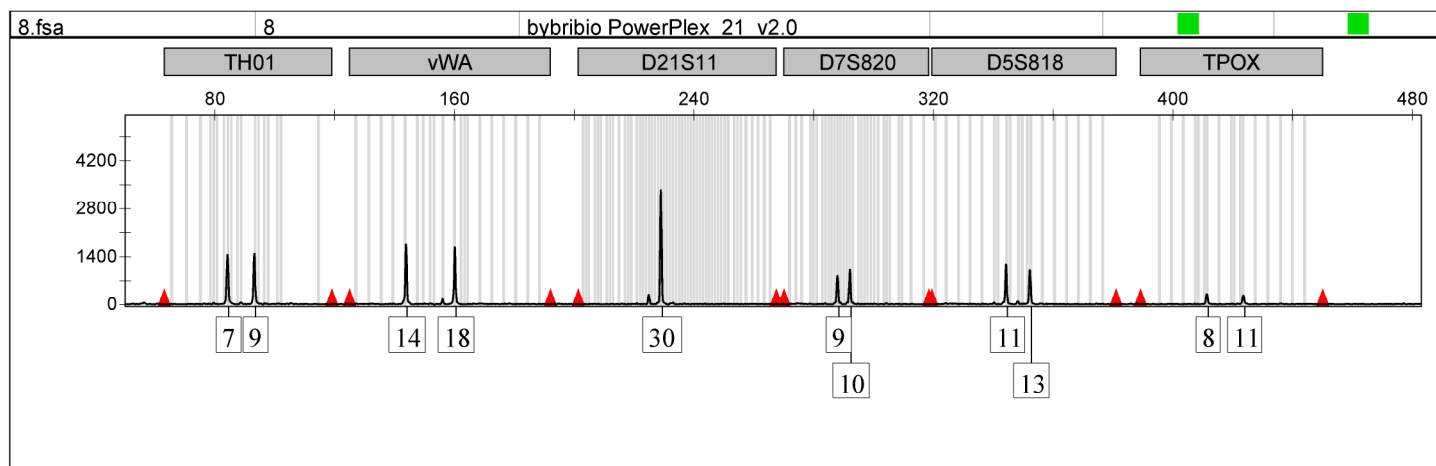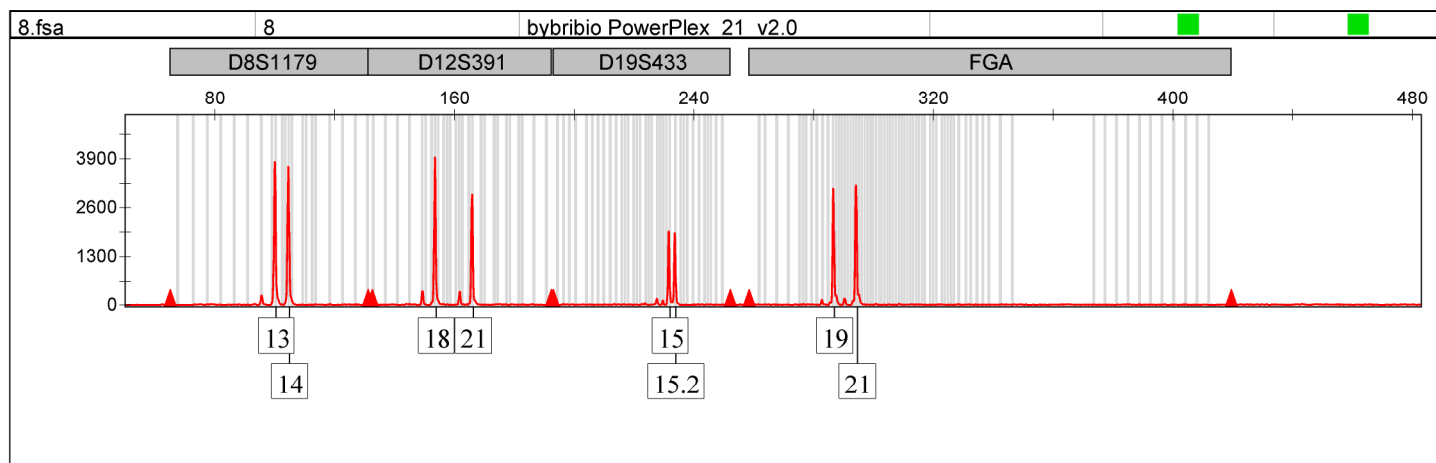

Supplement: Supplementary file 4 — Source Data [file 41467_2022_33759_MOESM4_ESM.zip › Source data/Supplementary fig 32-40/Supplementary Fig 36b.pdf]

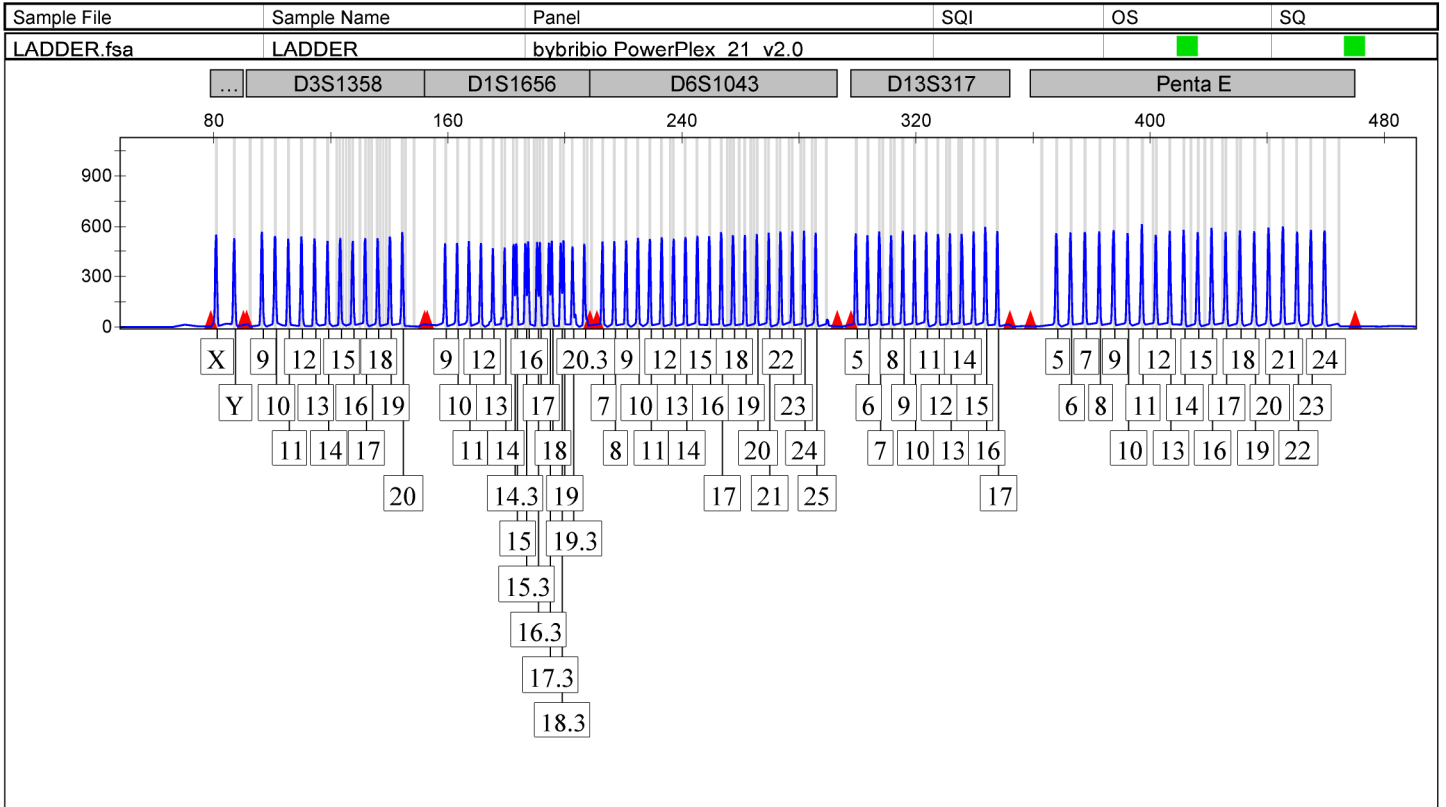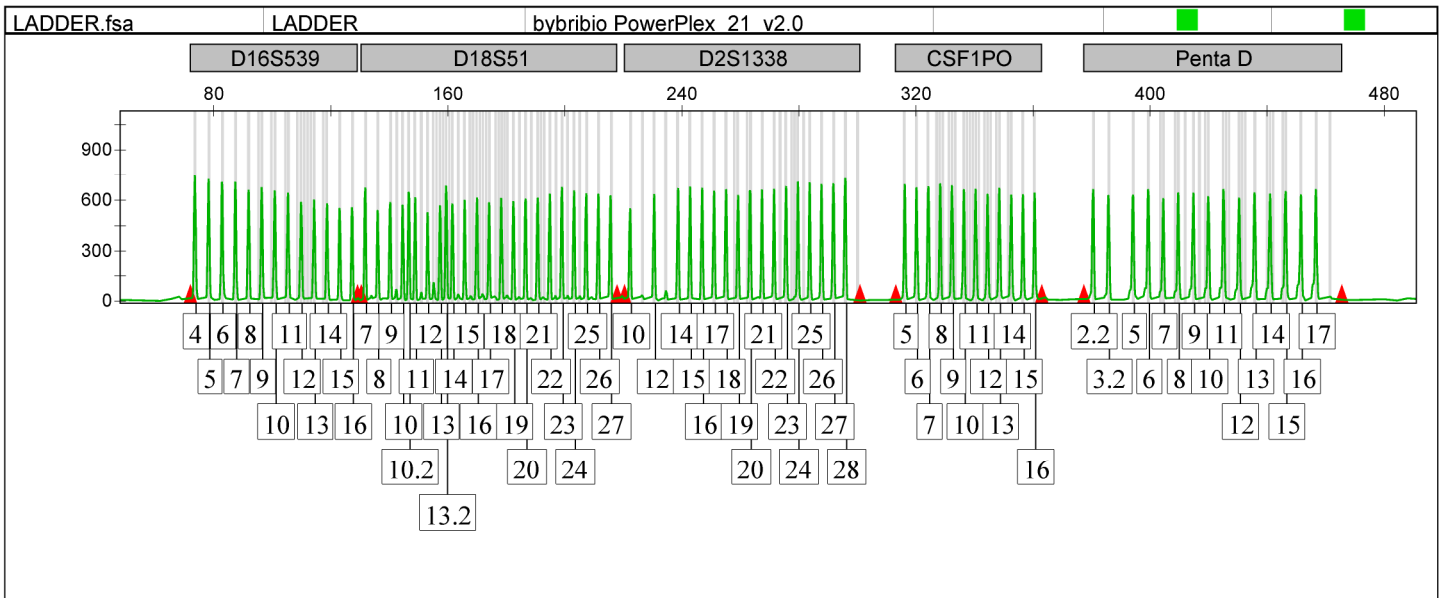

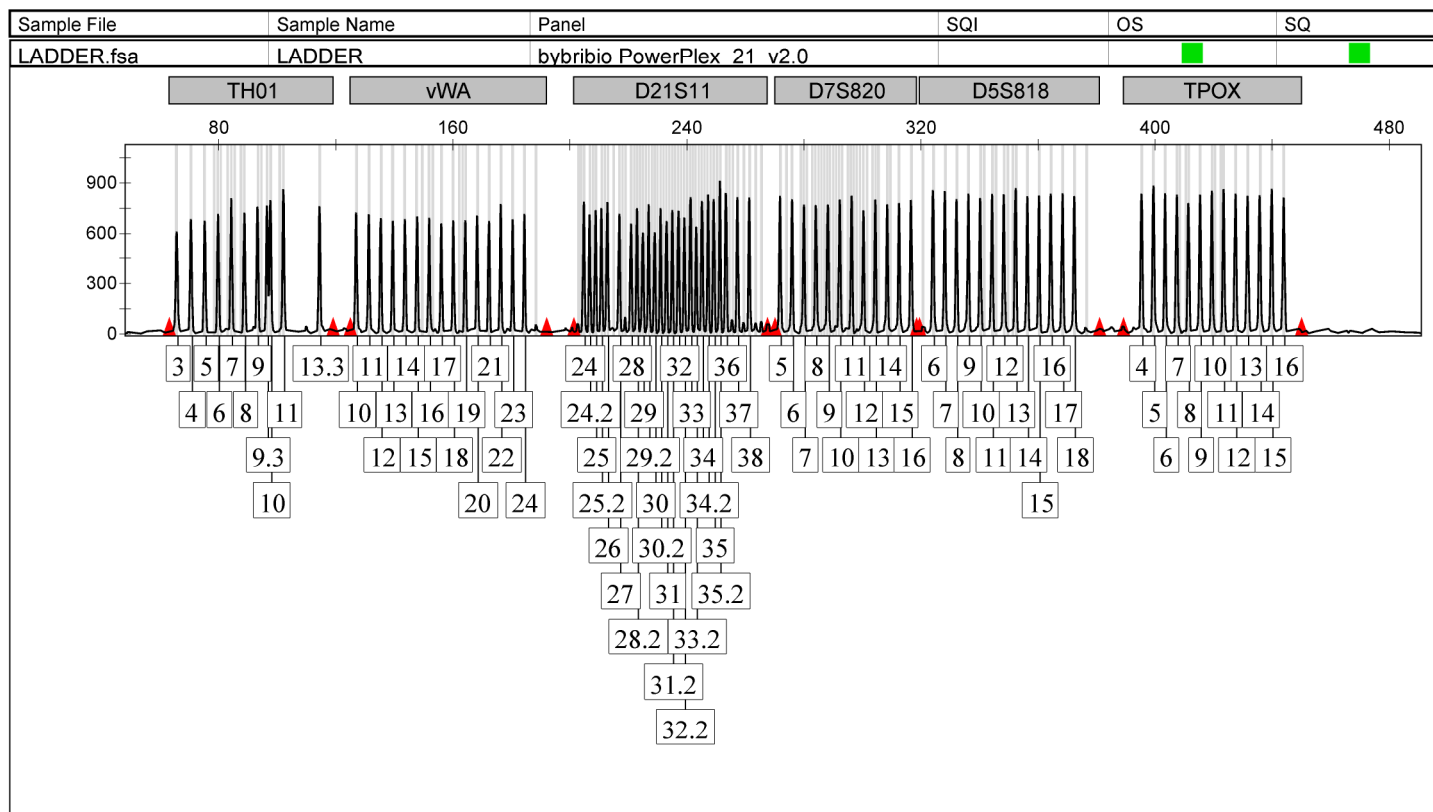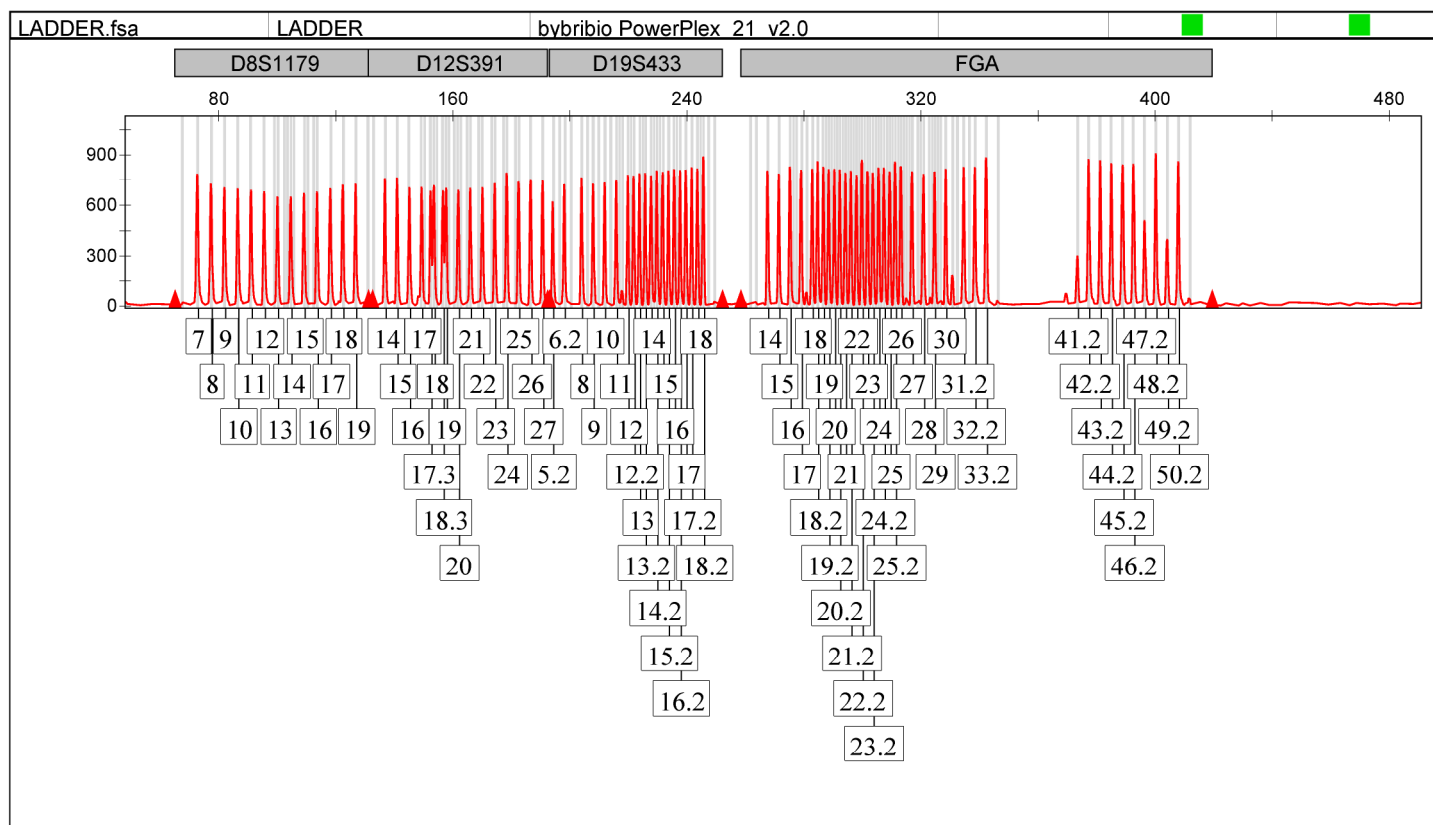

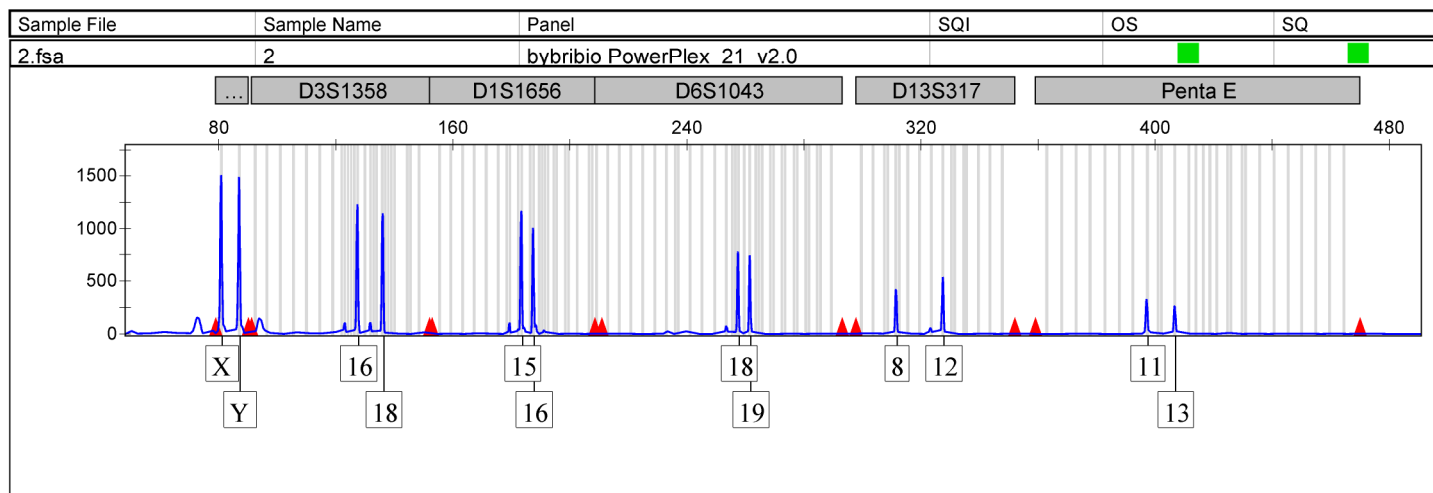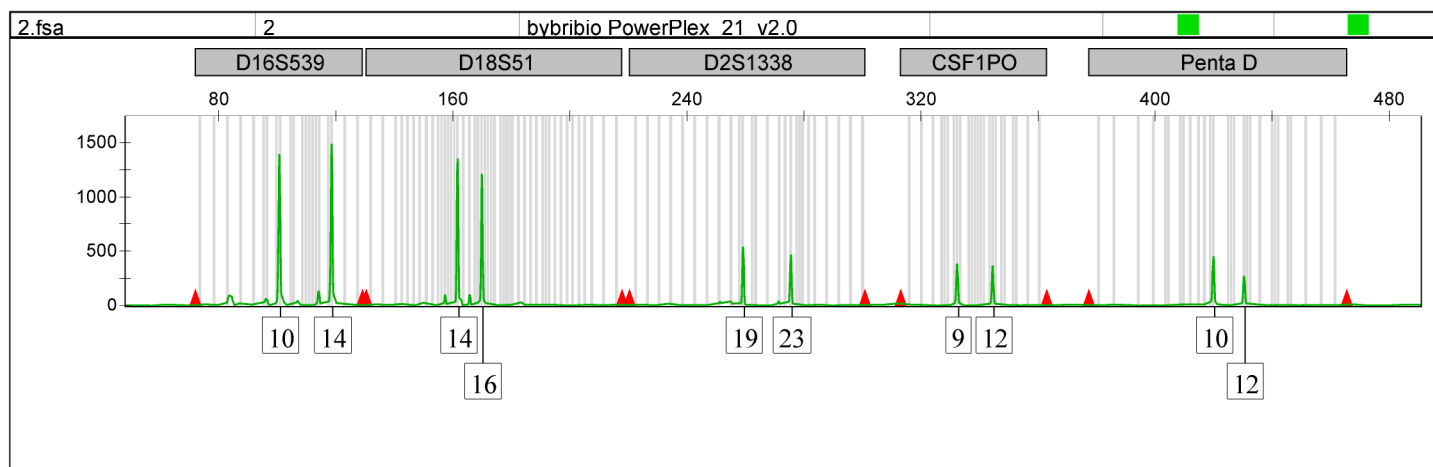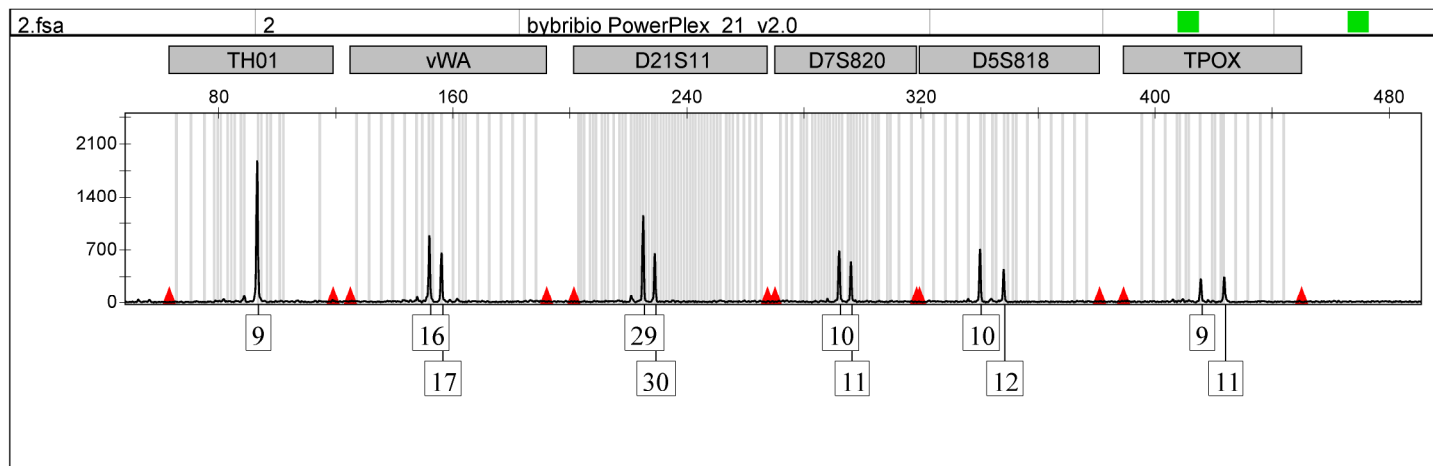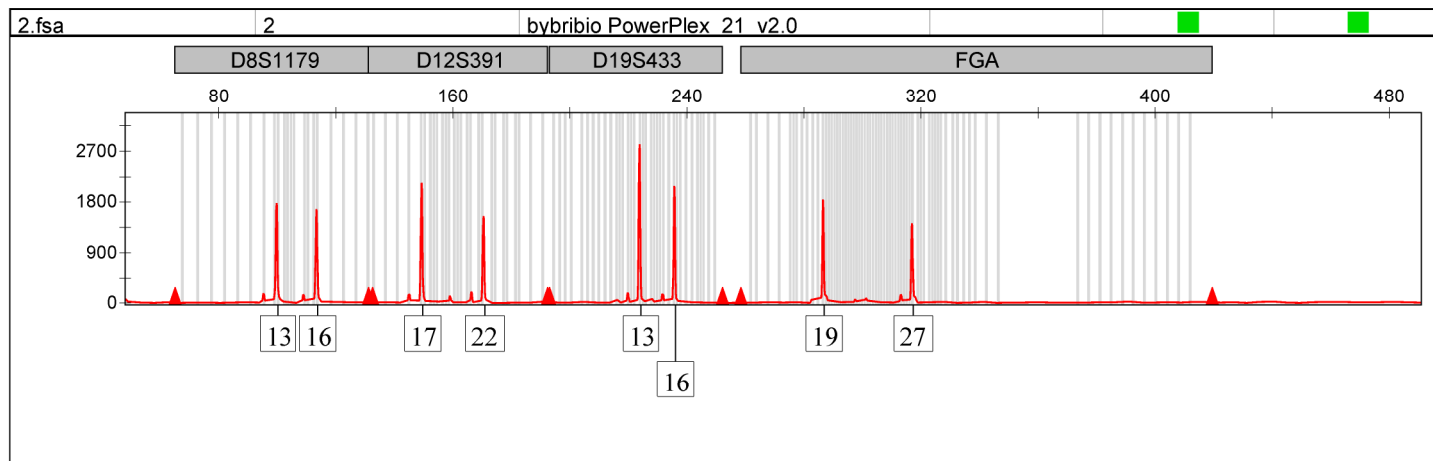

Supplement: Supplementary file 4 — Source Data [file 41467_2022_33759_MOESM4_ESM.zip › Source data/Supplementary fig 32-40/Supplementary Fig 37b.pdf]

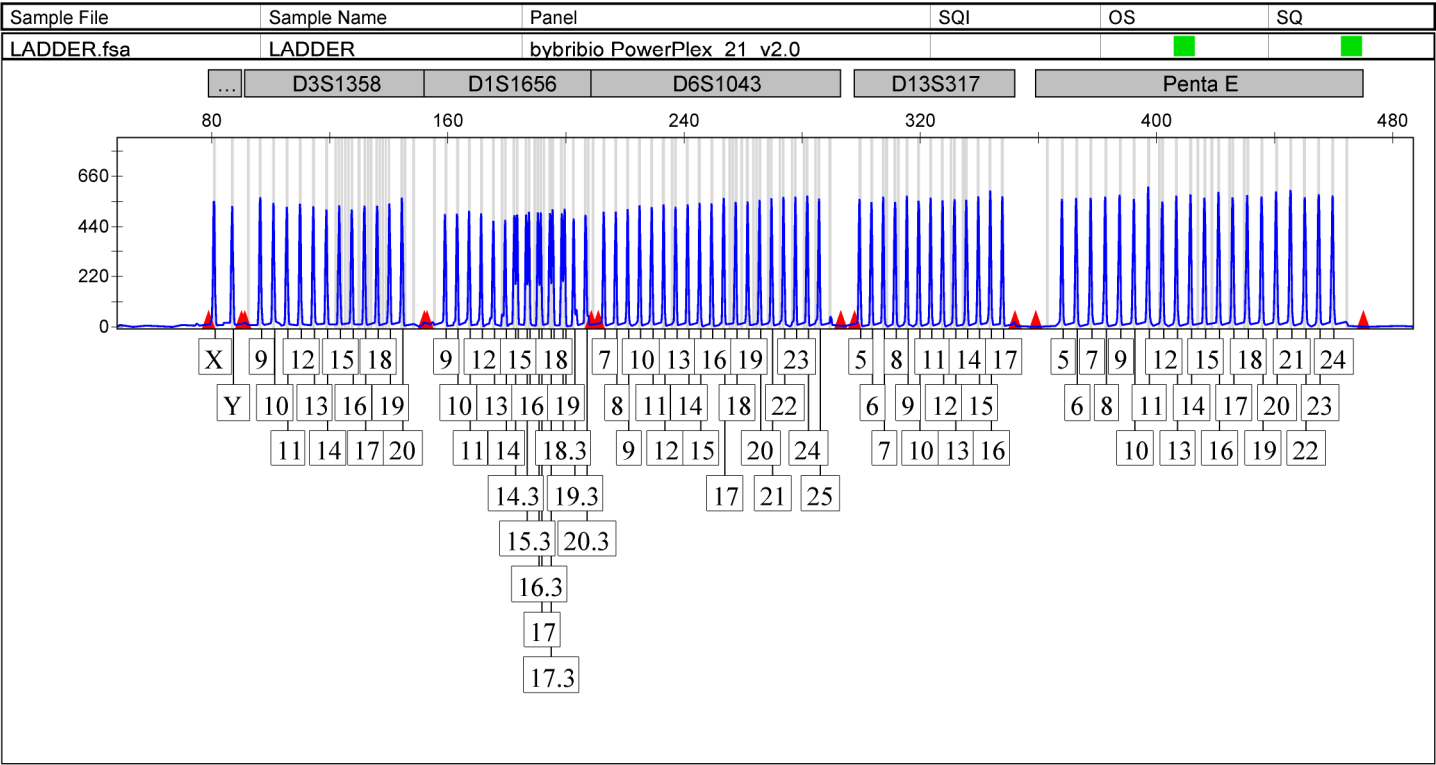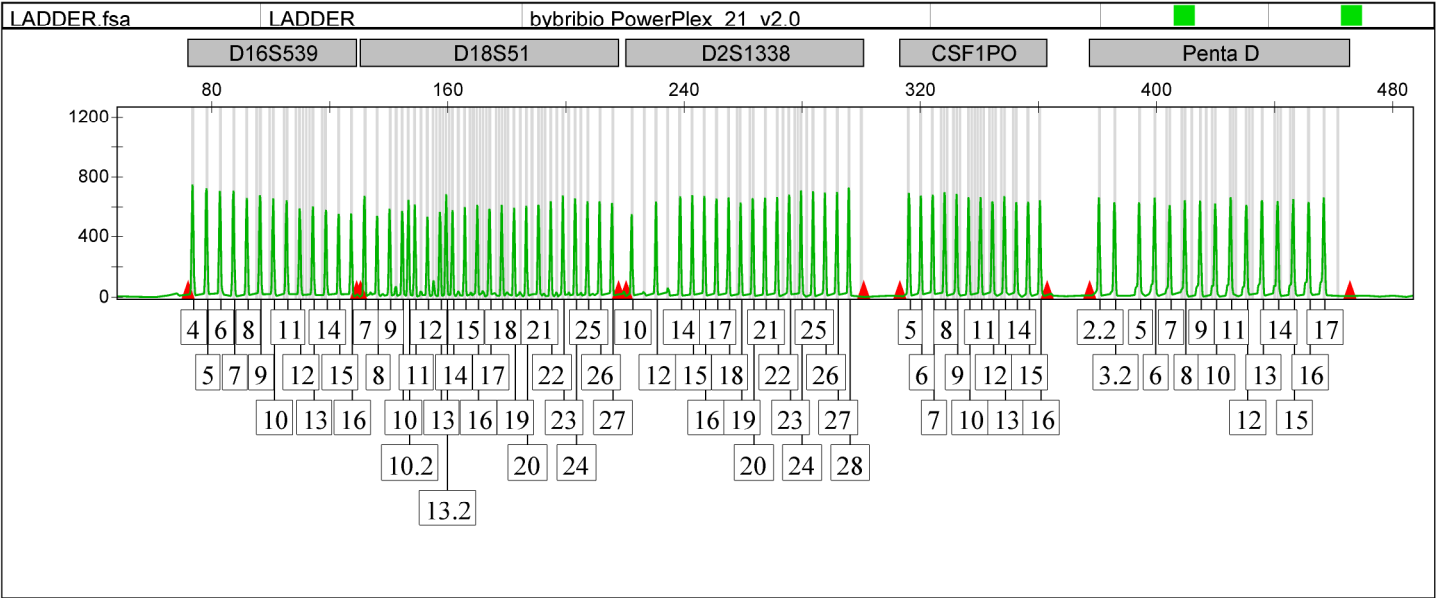

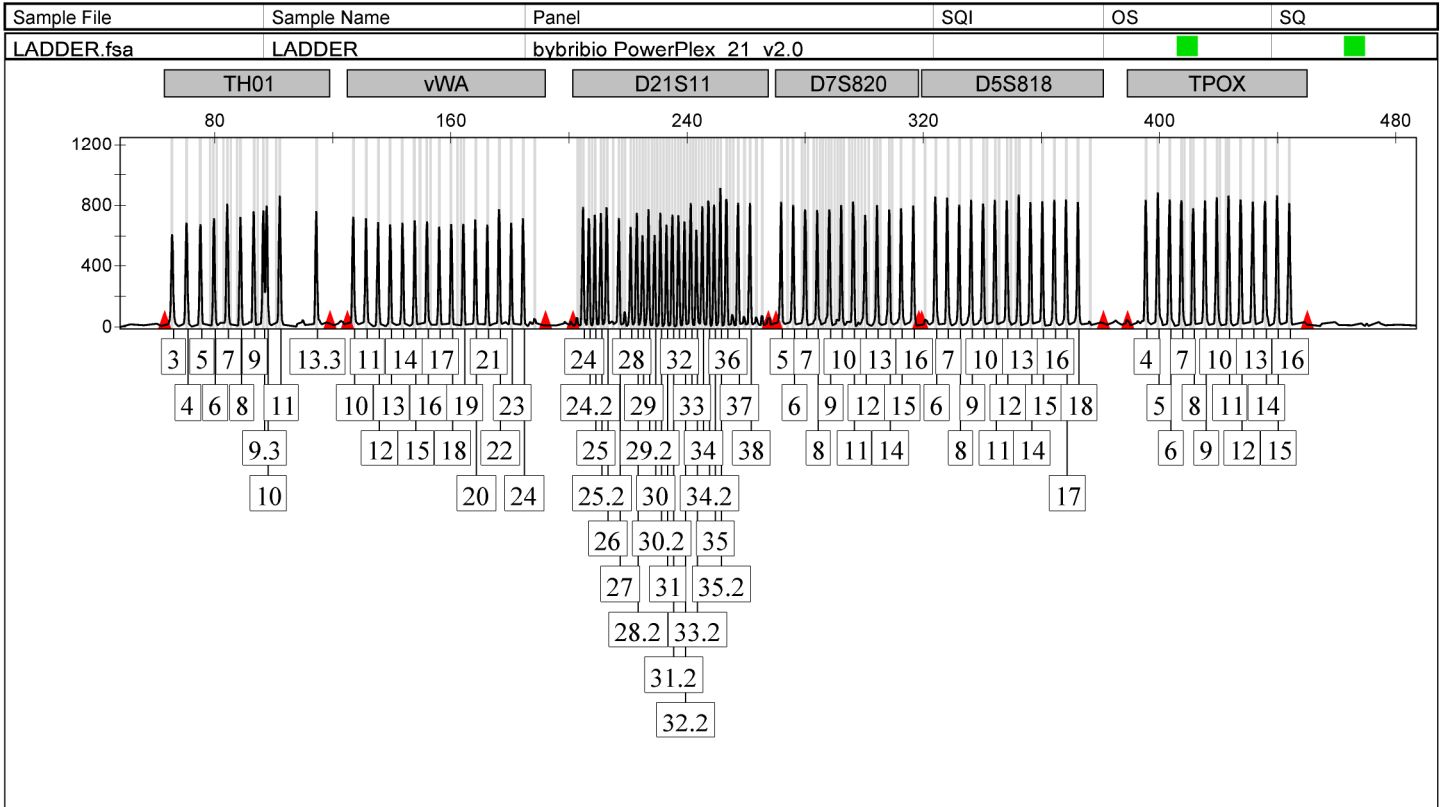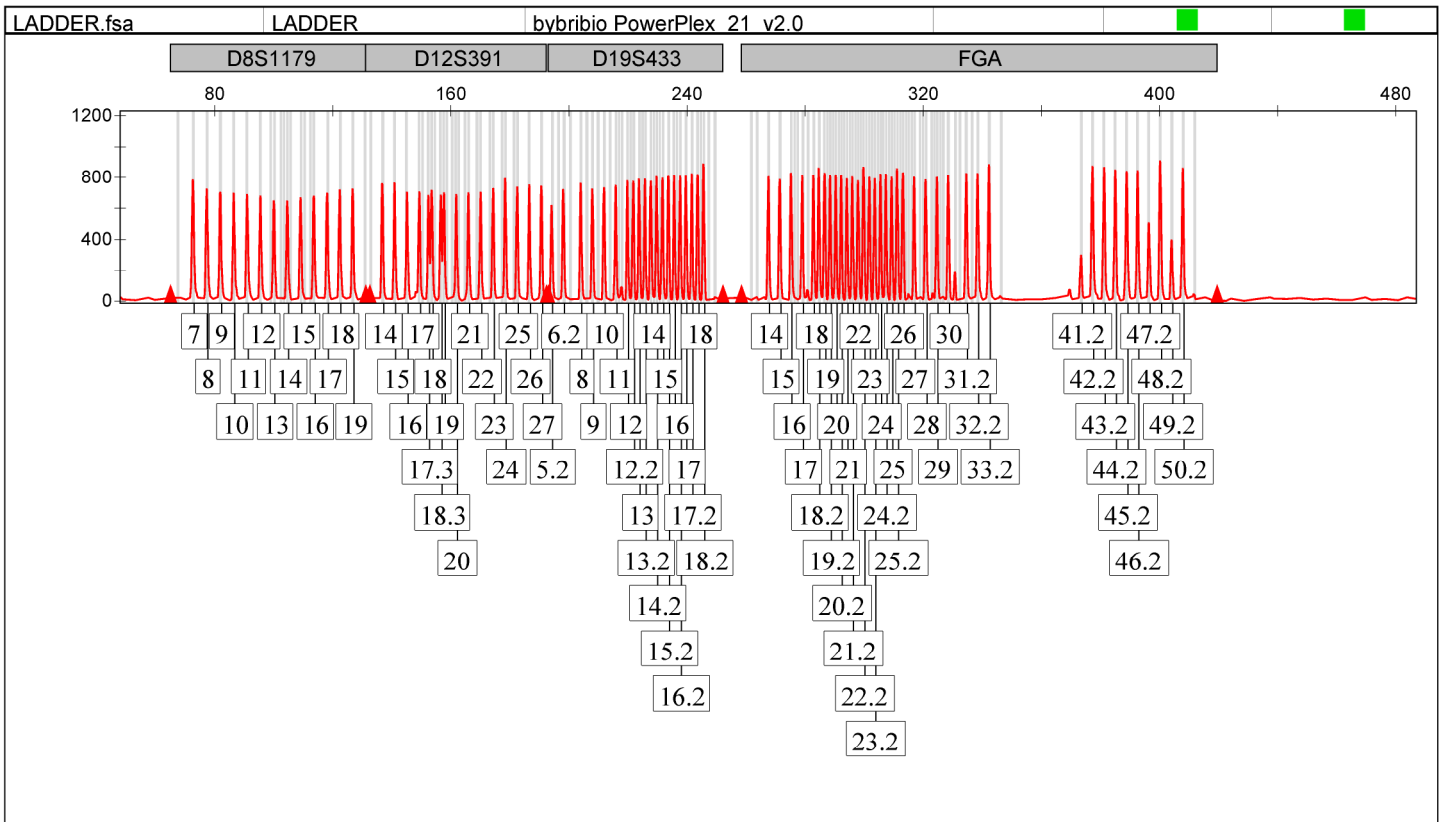

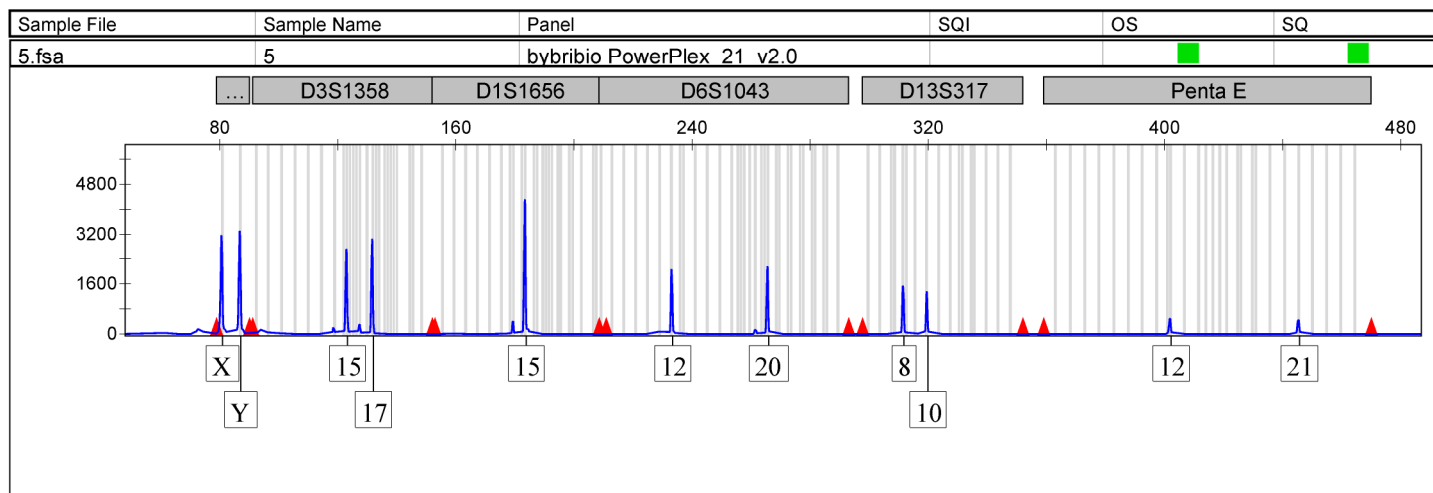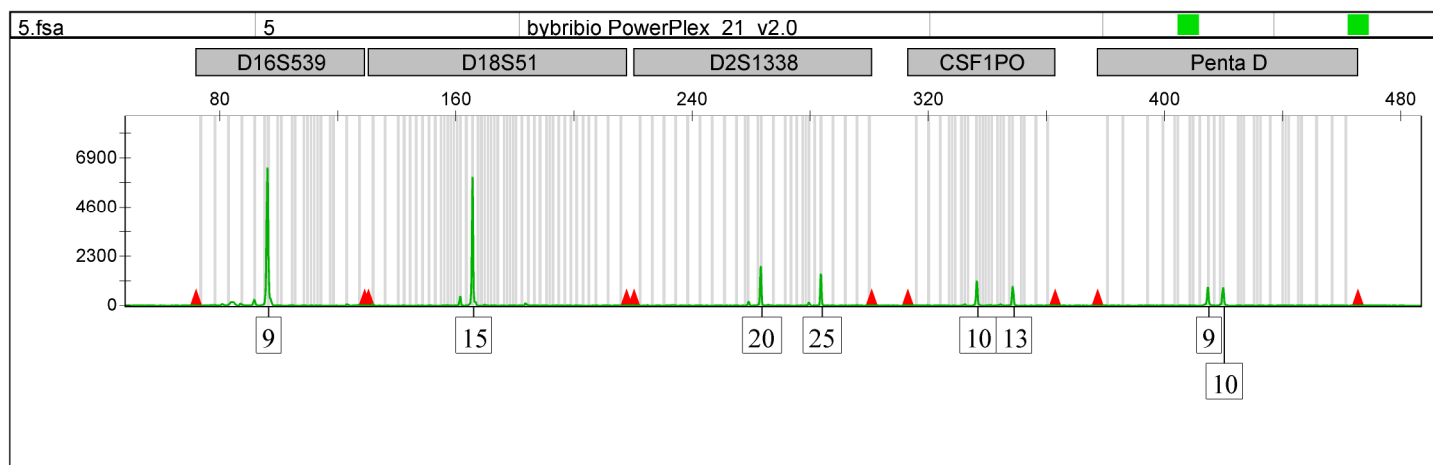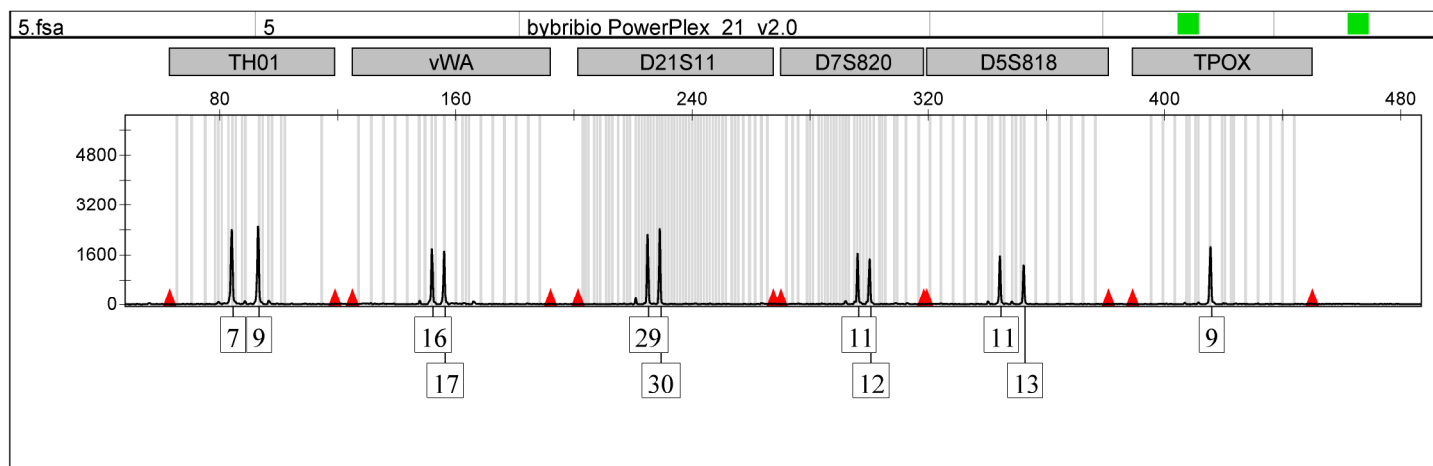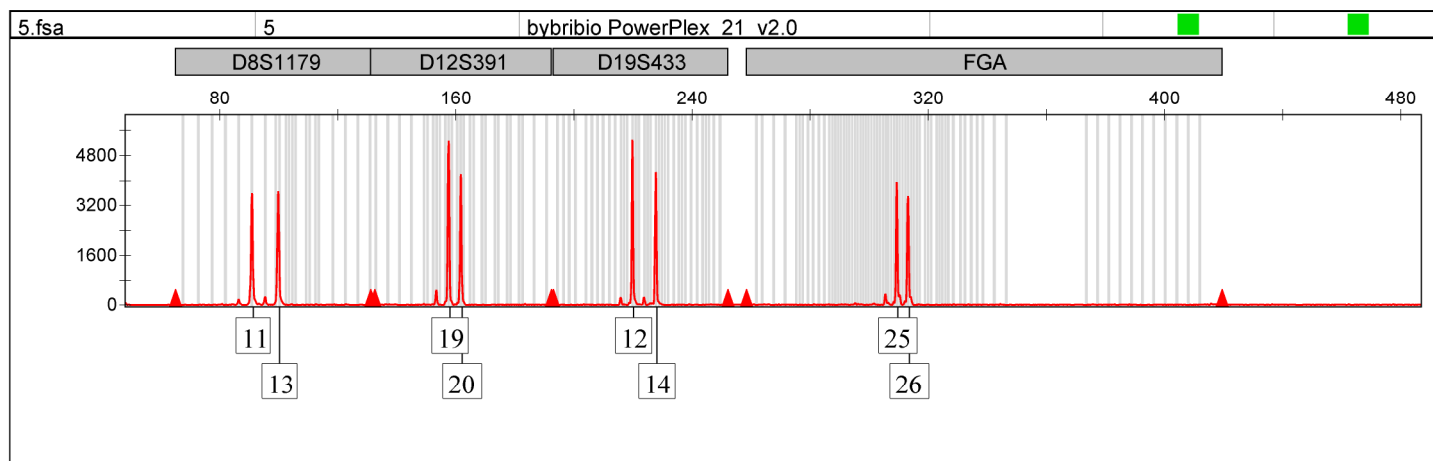

Supplement: Supplementary file 4 — Source Data [file 41467_2022_33759_MOESM4_ESM.zip › Source data/Supplementary fig 32-40/Supplementary Fig 38b.pdf]
